# Climate damage projections beyond annual temperature

**DOI:** 10.1038/s41558-024-01990-8

**Published:** 2024-04-17

**Authors:** Paul Waidelich, Fulden Batibeniz, James Rising, Jarmo S. Kikstra, Sonia I. Seneviratne

**Affiliations:** 1 Climate Finance and Policy Group, ETH Zurich, Zurich, Switzerland; 2 Institute for Atmospheric and Climate Science, ETH Zurich, Zurich, Switzerland; 3 Oeschger Center for Climate Change Research, University of Bern, Bern, Switzerland; 4 Climate and Environmental Physics, Physics Institute, University of Bern, Bern, Switzerland; 5 School of Marine Science & Policy, University of Delaware, Newark, DE US; 6 Centre for Environmental Policy, Imperial College London, London, UK; 7 Grantham Institute for Climate Change and the Environment, Imperial College London, London, UK; 8 Energy, Climate and Environment (ECE) Program, International Institute for Applied Systems Analysis, Laxenburg, Austria

**Keywords:** Economics, Climate-change impacts, Projection and prediction, Environmental economics

## Abstract

Estimates of global economic damage from climate change assess the effect of annual temperature changes. However, the roles of precipitation, temperature variability and extreme events are not yet known. Here, by combining projections of climate models with empirical dose–response functions translating shifts in temperature means and variability, rainfall patterns and extreme precipitation into economic damage, we show that at +3 ^°^C global average losses reach 10% of gross domestic product, with worst effects (up to 17%) in poorer, low-latitude countries. Relative to annual temperature damage, the additional impacts of projecting variability and extremes are smaller and dominated by interannual variability, especially at lower latitudes. However, accounting for variability and extremes when estimating the temperature dose–response function raises global economic losses by nearly two percentage points and exacerbates economic tail risks. These results call for region-specific risk assessments and the integration of other climate variables for a better understanding of climate change impacts.

## Abstract (web summary)

Existing global economic damage assessments only focus on the impacts induced by annual temperature changes. Including variability and extremes of temperature and precipitation in climate damage projections raises global gross domestic product losses and exacerbates global disparities of economic damage.

## Main

Projections of economic damage from climate change are key for evaluating climate mitigation benefits, identifying effects on vulnerable communities and informing discussions around adaptation needs, as well as loss and damage financing. On a global or country level, such assessments have focused on how projected changes in annual mean temperatures affect gross domestic product (GDP)^1–4^. However, the widespread losses in recent years driven by flooding and drought suggest that precipitation variability and extremes are similarly important^5,6^. Anthropogenic forcing is increasing the frequency and intensity of precipitation extremes and variability on multiple scales, altering daily temperature patterns and driving an overall increase in precipitation over land^7,8^. Continued global warming is expected to exacerbate these trends, potentially with uneven impacts across regions^5,9,10^. Therefore, including precipitation, variability and extremes can improve the precision, comprehensiveness and interpretability of climate change damage estimations^11^.

Economic damage from climate change can be assessed either bottom-up by quantifying, valuating and aggregating specific impacts (for example, crop failures or labour supply changes) or top-down by identifying the statistical relationship between observed climatic shifts and economic growth. While both approaches have advantages and limitations, top-down approaches usually neglect climatic shifts beyond annual temperature changes^12^. To address this shortcoming, recent studies have estimated the relationship between macrolevel income and a wider range of climatic indicators, such as total precipitation^13–15^, temperature variability^16,17^ or temperature and precipitation extremes and anomalies^14,18,19^. However, these studies do not investigate how much the inclusion of these climate indicators alters previous economic assessments of climate change, which is highly relevant for policy-making and future adaptation. A notable exception is ref. ^15^, which projects the effects of annual precipitation and temperature shifts on inequality. A comprehensive assessment of the projected economic impacts of intense periods of precipitation and temperature anomalies, however, is still missing.

In this study, we draw upon recent advances in estimating dose–response functions, which relate shifts in various climate indicators (total precipitation, temperature variability, precipitation anomalies and extremes) to GDP changes^14^. Combining these functions with projections from 33 models of Coupled Model Intercomparison Project Phase 6 (CMIP6), including two large ensembles, we investigate how including these indicators affects the understanding of future economic impacts at different global warming levels. Variability and extremes introduce substantial climatic and associated economic uncertainties and we conduct a variance decomposition to determine the main uncertainty drivers. Furthermore, we explore how including variability and extremes in empirical regressions alters the dose–response function for annual mean temperature, which the extant literature has estimated controlling only for annual precipitation^1,2,4,20,21^.

## Projecting GDP impacts for six climate indicators

Compared to annual temperature, future changes in precipitation and temperature variability under climate change are subject to high uncertainties^8,22,23^. To capture these uncertainties, we use projections from various CMIP6 models^10^ to analyse four climate indicators besides annual mean temperature and annual precipitation: (1) day-to-day temperature variability (how much daily temperatures deviate from monthly means); (2) extreme precipitation (the annual sum of precipitation on days with exceptionally high precipitation exceeding the historical 99.9th percentile); (3) monthly precipitation deviation (how much monthly precipitation deviates from historical means throughout the year); and (4) the number of ‘wet days’ with precipitation above 1 mm d^−1^. These indicators have been linked to forcing from GHGs^24,25^ as well as to income growth using a global sample^14,16^. Considering all indicators in one coherent estimation framework is crucial because variability and extremes correlate strongly with annual temperature, total precipitation and each other (Supplementary Fig. 3). Therefore, combining dose–response functions from different estimations risks double-counting impacts. Notably, our coherent estimation framework^14^ does not model damage from droughts and heat waves. Therefore, we include heat in a complementary analysis, whereas we find no significant statistical link to economic growth for droughts, potentially due to limited spatial and temporal resolution and impact heterogeneity across regions (Supplementary Appendix F).

**Fig. 1:**
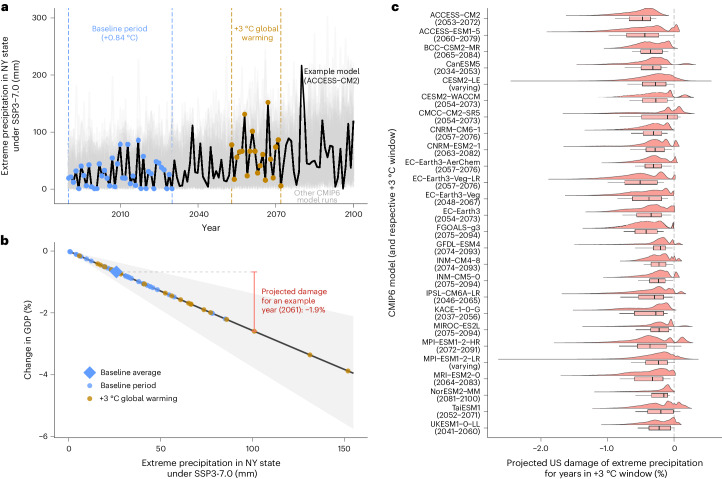
Illustrative example of GDP impact projections for one example indicator (extreme precipitation) and region (NY state) at +3 °C global warming. **a**, Projected extreme precipitation under SSP3-7.0 for an example model run (ACCESS-CM2, black) and other CMIP6 model runs (grey). Vertical lines denote the baseline period (blue) and the +3 °C global warming window (brown). **b**, Dose–response function for extreme precipitation (black line) and 95% confidence interval (grey area). Coloured dots and the blue diamond represent extreme precipitation levels from **a** and the baseline period average. The red error bar illustrates the difference between the dose–response function for an example year (2061) and the baseline average, which equals the projected damage for this year. Dose–response function values are transformed from the original logarithmic changes to percentage of GDP by exponentiating and subtracting one (Methods). **c**, Distribution of projected extreme precipitation damage at +3 °C by CMIP6 model. Boxplot centre, hinges and whiskers denote median, upper/lower quartiles and upper/lower deciles, respectively. For the CESM2-LE and MPI-ESM1-2-LR large ensembles, the +3 °C global warming-level window varies across ensemble members. Source data

We illustrate our approach for the example of extreme precipitation impacts on economic output for New York State at +3°C of global warming (Fig. 1). On the basis of how a given CMIP6 model and scenario project the respective climate indicator (Fig. 1a), we compare the GDP impacts in a given year against the average impacts if the climate were to remain constant at levels of a recent baseline period (Fig. 1b)^2,15^. For each model and scenario, the baseline period is the 41-year period during which global warming equals the historical warming between 1979 and 2019 (+0.84 °C), which is the climatic baseline used for estimating the dose–response functions used here (Methods)^14^. We then repeat this procedure for different CMIP6 models and potential damage parameter estimates based on statistical uncertainty and aggregate results to the national level. This yields a distribution of GDP impacts for each country featuring all years in each model and scenario associated with the same global warming level (Fig. 1c). Therefore, the main sources of uncertainty in our GDP impact distribution for a given global warming level and territory are (1) internal variability for the same CMIP6 model because the magnitude of extremes can vary strongly from year to year and, for large ensembles, across model runs, (2) statistical uncertainty in the dose–response functions and (3) projection differences between CMIP6 models.

## Global results

**Fig. 2:**
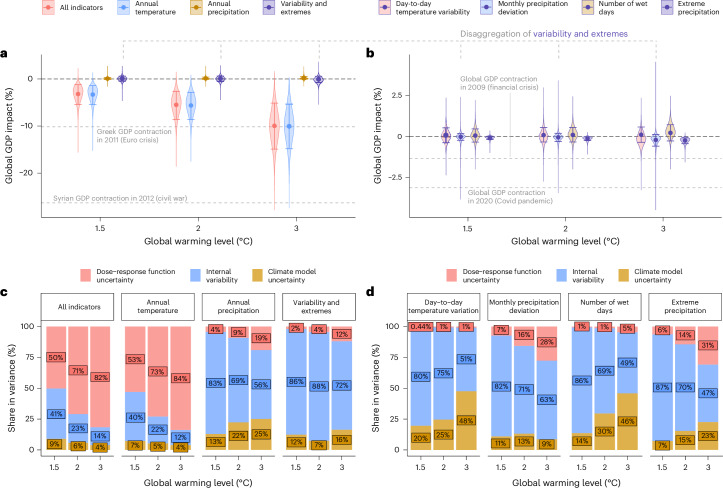
Distribution and variance decomposition of global GDP impacts. **a**, Points and the error bars centred around them denote the mean and upper-to-lower-decile range, respectively. ‘Variability and extremes’ comprises the four indicators in **b**. Labelled grey horizontal lines denote example growth rates in real GDP^28^. **b**, Same as **a**, with ‘variability and extremes’ impacts disaggregated by indicator. **c**, Variance decomposition for the combined GDP impacts of all climate indicators and for indicator-specific impacts. Indicator-specific decompositions are feasible because impacts in the underlying regression model are additive and hence can be projected separately. For absolute variances and coefficients of variation, see Supplementary Figs. 8 and 9. **d**, Same as **c**, with ‘variability and extremes’ disaggregated by indicator. Source data

We examine the impact on global GDP for all indicators combined, as well as the separate impacts from annual temperature, annual precipitation and the four variability and extremes indicators (Fig. 2a). Global GDP is estimated to be 3.2% lower (lower/upper decile: 1.2–5.4%) at +1.5 °C of global warming, compared to a world with no further climate change beyond recent levels. At +3 °C, global GDP decreases by 10.0% (5.1–14.9%). When disaggregated by climate indicator, global impacts are strongly determined by the annual mean temperature changes, which account for a GDP reduction of 10.0% at +3 °C. This estimate is consistent with recent top-down studies focusing exclusively on damage from annual temperature changes and projecting impacts of 7–14% of GDP per capita loss by the end of the century at global warming levels of more than +4 °C (refs. ^4,8,20^); with other top-down studies estimating damage even higher^2^ as a result of their assumption that temperature changes impact growth trajectories persistently^12,26,27^. For context, a 10% reduction exceeds the GDP loss of the COVID-19 pandemic when global growth plummeted from +2.6% in 2019 to −3.1% in 2020 or the effect of the global financial crisis in 2009 when global output shrunk by −1.3% (ref. ^28^). Importantly, recent research suggests that damage attributed to annual temperature covers heat wave impacts at least partially^18^. Indeed, when disentangling the two, we find that almost half of annual temperature damage can be attributed to heat extremes (Supplementary Appendix F).

By contrast, increases in annual precipitation in many areas have a small positive impact on global GDP (0.2% at +3 ^°^C warming) and the combined impact of the variability and extremes indicators remains centred around zero. While this suggests a lack of signal, disaggregating projections by individual indicators reveals otherwise (Fig. 2b). At +3 °C, extreme precipitation reduces global GDP by 0.2% (0.1–0.4%), with 99% of our impact distribution indicating economic losses as extreme precipitation increases around the globe (Supplementary Fig. 5). Notably, these impacts are much lower than annual temperature damage. This is somewhat expected because extremes have a lower temporal and spatial correlation. Therefore, aggregation from daily, location-specific events to annual indicators and country-level projections reduces signals more strongly compared to annual mean temperature^13,14^. However, a 0.2% GDP loss due to extreme precipitation globally for an average year still represents a tenth of the damage caused by the catastrophic 2022 floods in Pakistan, estimated at 2.2% of GDP^29^. Global GDP losses from extreme precipitation are compensated, on average, by temperature variability reductions in higher latitudes (+0.1% of global GDP at +3 °C)^24,30^ and fewer wet days (+0.2%). However, only 63% and 74% of the impact distribution imply global economic gains for these indicators, respectively. Monthly precipitation deviations, on average, add to global GDP losses (0.2% at +3 °C), but uncertainty ranges remain centred around zero.

To explore uncertainty drivers, we decompose the variance in GDP impacts from each climate indicator into statistical dose–response function uncertainty, climate model uncertainty and internal variability (Fig. 2c). For annual temperature damage, uncertainty is primarily driven by the dose–response function, particularly at higher global warming levels. By contrast, impact uncertainty for annual precipitation and variability and extremes is dominated by internal variability. Additional analyses focusing on the two large ensembles in our sample suggest that this stems primarily from interannual variation within model runs rather than differences across ensemble members (Supplementary Figs. 15 and 16). Moreover, disagreement between CMIP6 models plays either a comparable or a more substantial role than dose–response function uncertainty for these additional indicators (except for monthly precipitation deviation) and is particularly pronounced for day-to-day temperature variability and the number of wet days (Fig. 2d). Notably, the share of climate model uncertainty decreases in global warming for annual temperature impacts but not for variability and extremes because even for a stronger global warming signal, GDP impact projections do not converge between models.

## Country-level results

**Fig. 3:**
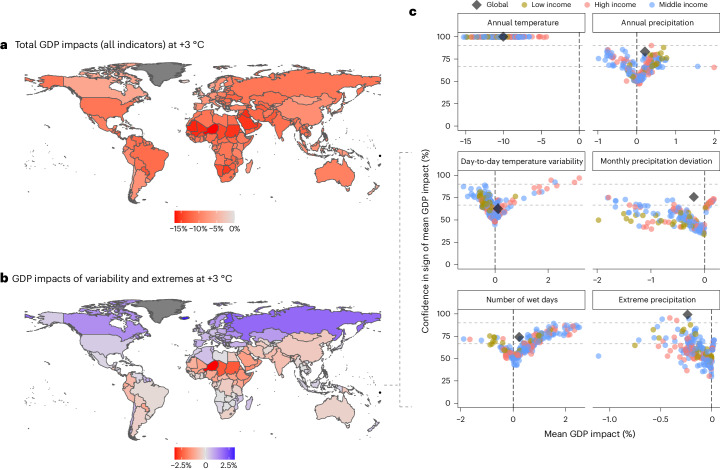
Country-level GDP impacts and uncertainty at +3 °C of global warming. **a**, Mean GDP impact at +3 °C of global warming for sovereign countries (other territories in dark grey) considering all six indicators in **c**, using shapefiles from ref. ^42^. **b**, Same as **a** but only considering the bottom four ‘variability and extremes’ indicators in **c**. **c**, Mean GDP impact (*x* axis) and share of the impact distribution agreeing with the sign of the mean (*y* axis) for sovereign countries by World Bank income group (colour) and the global economy (grey diamond) at +3 °C. Middle income comprises lower- and upper-middle-income countries for conciseness. Dashed horizontal lines denote thresholds for 66% and 90% likelihood following IPCC terminology^8^. Source data

Because global aggregates risk masking heterogeneities across regions, we further investigate the combined country-level GDP impacts from all six climate indicators at +3 ^°^C of warming (Fig. 3a). All countries face GDP losses, in line with recent evidence that climate change might not benefit cooler countries economically^20^. Impacts are more severe in the Global South and highest in Africa and the Middle East, where higher initial temperatures make countries particularly vulnerable to additional warming. Considering the combined GDP impact of all four variability and extremes indicators reveals a clear North–South divide (Fig. 3b). While for higher latitudes, the decrease in temperature variability and, for some countries, wet days (Supplementary Fig. 5) mitigates overall GDP damage, variability and extremes exacerbate GDP losses in most parts of the Global South. However, these effects vary substantially across the full distribution of projected impacts for each country.

Annual temperature is the only indicator where negative impacts arise for at least 90% of our impact distribution for all countries (upper dashed line in Fig. 3c), including substantial impacts in several colder countries partially because the temperature dose-response function deployed here implicitly features damages from higher inter-annual temperature variability as well (Supplementary Appendix C). Annual precipitation increases benefit most countries on average, but for many countries, less than two-thirds of the distribution supports the sign of expected impacts (lower dashed line). For day-to-day temperature variability, we find a clear divide between relatively certain gains for a few high-income countries and less certain, smaller losses for many lower-income countries as a result of variability increases in lower latitudes^24^. While extreme precipitation increases in most regions, projected damages are highest and least uncertain for middle- and high-income countries in higher latitudes such as China and the United States^31^. In contrast, low-income countries are more likely to face losses from shifts in precipitation deviation and wet days, but high uncertainties limit the conclusions that can be drawn.

## Overall impact of including variability and extremes

**Fig. 4:**
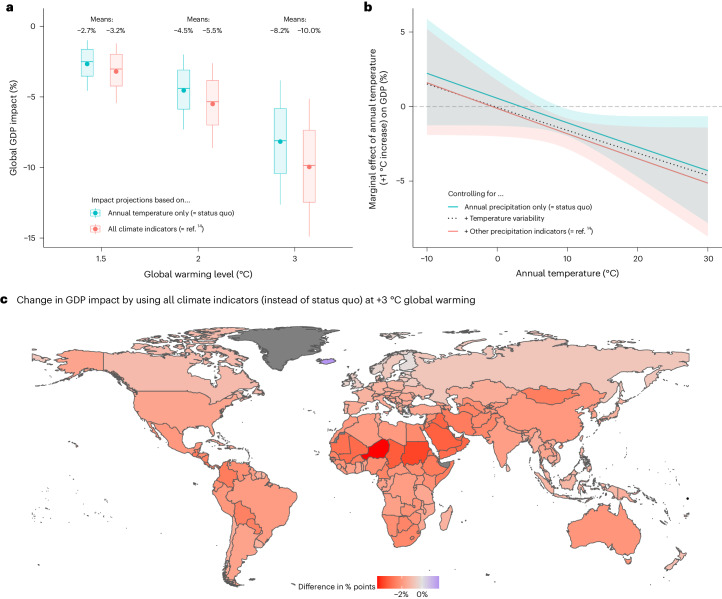
Comparison with status quo impact projections based on annual temperature only. **a**, Dots represent mean values of the GDP impact distribution, while boxplot centre, hinges and whiskers denote median, upper/lower quartiles and upper/lower deciles, respectively. **b**, GDP impact of a +1 °C increase in the annual temperature of a territory for different initial temperature levels (*x* axis) using mean marginal effects (lines) with 95% confidence intervals around them (shaded area). Estimated using Supplementary equation (22) and the regression models in Supplementary Table 7 (columns 1, 2 and 5). No confidence interval shown for ‘+ Temperature variability’ for visual conciseness. **c**, Difference in mean GDP impacts between our main approach and the status quo approach at +3 °C for sovereign countries (other territories marked in dark grey), using shapefiles from ref. ^42^. Source data

The results in the previous sections seemingly suggest that including variability and extremes in GDP impact projections exacerbates disparities between higher- and lower-income countries (Fig. 3) but does not substantially alter the implications of climate change for global GDP (Fig. 2), particularly since annual temperature damages capture heat wave impacts to some extent already. However, providing an apples-to-apples comparison with the recent climate economics literature requires calculating damage on the basis of the current ‘status quo’ approach, which (1) projects only damage from annual temperature changes and (2) estimates the relationship between income growth and annual temperature controlling only for annual precipitation^1,2,4,20,21^. When comparing the resulting global GDP impacts following this status quo methodology to our approach, which (1) projects damage for all six indicators and (2) controls for all our climate indicators when estimating the temperature dose–response function (Fig. 4a), we find that including variability and extremes increases global damage at +3 °C of global warming by 1.8 percentage points (10.0% instead of 8.2%).

The main reason for this increase is that controlling for variability and extremes, instead of only for annual precipitation, increases the estimated effect of mean temperature changes (Fig. 4b). The marginal GDP impact of a +1 °C rise in annual temperature increases by more than 0.5 percentage points irrespective of the initial temperature level when all climate indicators are included as control variables (red line). Most of this effect is driven by including temperature variability (dotted line), which leads to higher impacts, particularly for colder regions. Therefore, the positive impacts of temperature variability in Fig. 3 obscure that, in fact, including this parameter leads to higher global damage since it disentangles potential benefits of reduced variability from the negative effects of temperature increases. As a result, including all climate indicators exacerbates GDP impacts across the globe (Fig. 4c).

## Exposure to tail risks

**Fig. 5:**
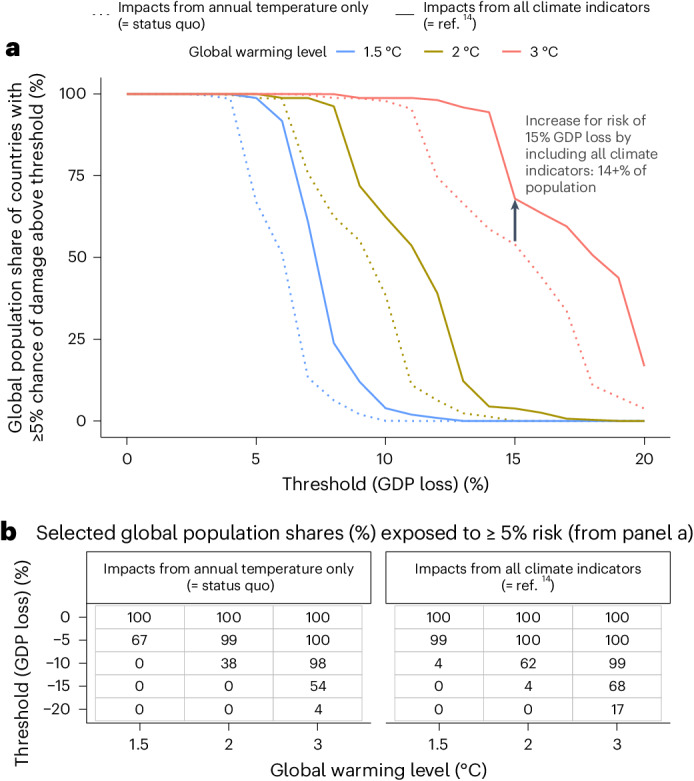
Exposure of global population to tail risks of GDP losses. **a**, Share of the current global population living in countries whose projected GDP impacts for a given warming level (colour) exceeds the respective threshold (*x* axis) for at least 5% of the GDP impact distribution, based on the status quo approach (dotted lines) and our main approach using all climate indicators (solid lines), respectively. The grey arrow and text annotation provide a reading example. **b**, Selected values from **a**. Source data

Aside from average impacts and the uncertainty around them, prudent risk management by policy-makers also requires information about tail risks. Therefore, we examine the percentage of the present global population living in countries that have a non-negligible chance (at least 5%) of suffering from damage exceeding different thresholds at different global warming levels (Fig. 5a), both for our main approach (solid line) and the status quo approach (dotted line). Even at +1.5 °C, tail risks are substantial, with 99% of the global population living in countries with a non-negligible risk of suffering GDP damage of 5% or higher if all climate indicators are included. Notably, including variability and extremes increases tail risks considerably (Fig. 5b). While under the status quo, 54% of the global population is projected to face damage of at least 15% with a likelihood of at least 5% at +3 ^°^C of warming, this increases to 68% of the population when variability and extremes are included. The share of the global population facing catastrophic impacts of 20% or higher with a 5% chance rises from 4% to 17%. However, disaggregating these results by individual climate indicators (Supplementary Fig. 6) highlights that the increase in global exposure to catastrophic climate change damage is primarily driven by higher temperature damage if underlying regression models control for more climate indicators than just annual precipitation and less by the direct impacts of these indicators on global GDP.

## Discussion

Taken together, our results illustrate that projecting top-down damage of variability and extremes exacerbates global disparities further. Aggregate GDP loss projections, however, remain primarily driven by the impacts of mean temperature changes, which seem to cover economic losses due to heat waves at least partially^18^—an important finding for climate–economy modelling that complementary assessments of economic damage should corroborate to disentangle different impact channels better. As a result, overall uncertainty in GDP losses is dominated by the temperature dose–response function. However, substantial climatic uncertainties still limit the understanding of direct impacts by variability and extremes, particularly for low-income countries, which are expected to suffer the most but exhibit the largest uncertainties.

For scholars studying the economic effects of climate change, our results suggest a potential downward bias in temperature damage estimates by not disentangling the impacts of changes in temperature means and temperature variability. Future studies estimating temperature dose–response functions should test how including variability and extremes indicators linked to economic growth alters their findings. Notably, such biases could also be caused by other climate indicators not explicitly considered here and their direction and magnitude are likely to vary by location^32^. Furthermore, since the signal clarity is highest for extreme precipitation, this indicator seems most suitable to be included in climate–economy calculations, such as the social cost of carbon.

While our results rest on strong empirical foundations and a wide range of state-of-the-art climate models, there are several reasons why actual GDP impacts may exceed our projections. First, while the temperature dose–response function seems to include heat impacts at least partially, the dose–response functions used here do not explicitly cover important climate extremes, most notably droughts^33^. Second, to be conservative, we abstract from the possibility that climatic shifts do not only change GDP growth in a given year but alter a country’s long-run growth trajectory persistently. While such persistence in GDP losses remains empirically debated^1,2,14,21,34^, it would increase damage estimates substantially^26,27^. Third, aggregation across time and space is more likely to reduce signals in precipitation patterns because of their lower spatial and temporal correlation compared to annual mean temperature^13,14^. For these reasons, our results should be seen as an important first step, but they certainly do not exclude the possibility of larger GDP losses. Furthermore, econometric-based dose–response functions such as the ones used here have several limitations; for example, the risk of conflating weather impacts with climatic shifts or the extrapolation of impacts to warming levels that go far beyond historical observations, with the implicit assumption that adaptation remains at historically observed levels^35,36^. In addition, specification questions can further exacerbate socioeconomic uncertainties^21^ and uniform dose–response functions for aggregate GDP can mask heterogeneities between countries, sectors and income segments^15^. Moreover, considering impacts in percentage of GDP implicitly assigns lower weights to poorer regions within countries that are disproportionately exposed to climate change risks^37^. Lastly, the distributions presented here might underestimate true climatic uncertainties for at least three reasons: (1) measurement imperfections in the reanalysis data underlying the dose–response functions, particularly for precipitation and lower-income regions^32,38^; (2) using single runs for most CMIP6 models may underestimate tail risk events (Supplementary Appendix E); and (3) not all CMIP6 models, despite representing the current frontier of global climatic projections, fully capture future changes in temperature variability and precipitation^24,25,39–41^.

Nevertheless, our study represents a key improvement in top-down damage projections, highlights the risks of omitting climate indicators beyond annual temperature, either as impact channels or control variables, and identifies the most promising fields for additional research. Building on our work, researchers could integrate further climate indicators, such as droughts, into a comprehensive assessment of climate change impacts. Aside from improvements in climate modelling, particularly for developing countries, this would also require more empirical studies to robustly identify the link between economic growth and different climatic extremes, ideally combined with an improved temporal or spatial resolution^17^. In addition, future research should assess the impact of controlling for these extremes on temperature dose–response functions and enhance the understanding of potential adaptation mechanisms and the persistence of GDP losses, ideally by consistently estimating and implementing persistence for each climate indicator under consideration.

## Methods

### Climatic data

Daily temperature and precipitation projections are taken from 33 CMIP6 models under two low-emission scenarios (Shared Socioeconomic Pathways SSP1-1.9 and SSP1-2.6) and one high-emission scenario (SSP3-7.0) to calculate bias-corrected, annual climate indicators for the 1850–2100 period. Owing to computational constraints, we use only one realization for most model–scenario pairs. However, to explore the role of intra-ensemble variation, we include 30 realizations of MPI-ESM1-2-LR and all 100 realizations of the CESM2-LE large ensemble under SSP3-7.0, which provides us with a total of 199 model-realization–scenario pairings (Supplementary Tables 1–3). Time series switch between historical scenarios and the respective Representative Concentration Pathway (RCP)–SSP pair in 2015 and are regridded onto a common 2.5° × 2.5° longitude–latitude grid using conservative remapping^43^.

Consistent with our source of empirically calibrated dose–response functions, which relies on ERA5 data^14^, we calculate annual average temperature *T*, annual total precipitation RA as well as four climate indicators using the equations listed below before downscaling and regridding the annual indicators from 2.5° to 0.25° (the grid resolution of ERA5). Notably, the indicators used here have been motivated and subjected to various robustness checks by previous studies^14,16^.

Day-to-day temperature variability:

1 $${\tilde{T}}_{\rm{x,t}}=\frac{1}{12}\mathop{\sum }\limits_{\rm{a=1}}^{12}{\left(\frac{1}{{D}_{\rm{a}}}\mathop{\sum }\limits_{\rm{d = 1}}^{{D}_{\rm{a}}}{({T}_{\rm{x,d,a,t}}-{\bar{T}}_{\rm{x,a,t}})}^{2}\right)}^{0.5}$$

where *T*_*x*,d,a,t_ is the temperature for grid cell *x* of day *d* of month *a* in year *t* and *D*_a_ ∈ {28, 30, 31} is the number of days in the respective month *a*. ${\bar{T}}_{\rm{x,a,t}}$ denotes the mean temperature in month *a* of year *t* for the respective grid cell.

Extreme precipitation:

2 $${\hat{\rm{RD}}}_{\rm{x,t}}=\mathop{\sum }\limits_{d=1}^{365}{R}_{\rm{x,d,t}}\times I({R}_{\rm{x,d,t}} > {R}_{\rm{x},\rm{99p9,base}})$$

where *R*_x,d,t_ is the precipitation of grid cell *x* on day *d* of year *t*, *I*() is an indicator function and *R*_x,99p9,base_ denotes the 99.9th percentile of daily precipitation in grid cell *x* over a historical baseline period.

Number of wet days with precipitation exceeding 1 mm d^−1^:

3 $${\rm{RD}}_{\rm{x,t}}=\mathop{\sum }\limits_{d=1}^{365}I({R}_{\rm{x,d,t}} > 1\,\rm{mm}\,{d}^{-1})$$

Grid-cell-level annual climate indicators are then aggregated to the subnational region level (ADM1) using the geospatial data from the Database of Global Administrative Areas (GADM, v.3.6) and area weighting.

Monthly precipitation deviation, which we calculate only at the ADM1 level and not at the grid-cell level, consistent with ref. ^14^:

4 $${\rm{RM}}_{\rm{i,t}}=\mathop{\sum }\limits_{\rm{a}=1}^{12}\frac{{R}_{\rm{i,a,t}}-{\bar{R}}_{\rm{i,a},{\rm{base}}}}{{\sigma }_{\rm{i,a},{\rm{base}}}}\times \frac{{\bar{R}}_{\rm{i,a},{\rm{base}}}}{{\bar{RA}}_{\rm{i},{\rm{base}}}}$$

where *R*_i,*a*,t_ denotes precipitation totals in month *a* of year *t* for a given ADM1-level region *i*. Variables denoted by a bar represent averages across the baseline period, either for the full year or for a specific month, while *σ*_i,a,base_ denotes the month-specific standard deviation across the baseline period for region *i*. As for all other climate indicators, region-level monthly precipitation *R*_i,a,t_ is derived from grid-cell-level values based on area weighting.

For the baseline-dependent climate indicators $\hat{\rm{RD}}$ and RM, our source of dose–response functions^14^ uses 1979–2019 as the historical baseline period, during which global warming relative to pre-industrial levels over 1850–1900 averaged +0.84 °C according to Berkeley Earth data (the best estimate for the observed warming and, in a previous version, used in the IPCC AR6; ref. ^8^). However, the 1979–2019 time period can differ climatically across CMIP6 models, which warm at very different paces^44^. To maintain consistency and ensure that all climate indicators are based on the same baseline in terms of global warming, we, therefore, identify the corresponding 41-year window during which global warming relative to pre-industrial levels over 1850–1900 averages +0.84 °C for each climate model-realization and scenario. Then, we use the +0.84 °C window to calculate all values with a ‘base’ subscript in equations (2) and (4). Warming-level windows for each model-realization–scenario pairing are calculated following the approach by ref. ^10^ and shown in Supplementary Tables 1–3. However, percentile-based indicators, such as our extreme precipitation measure, can exhibit artificial jumps at the end of the baseline period, causing potential frequency increases and discontinuities outside this period^10,45,46^. To correct this, we use the bootstrap resampling procedure developed by ref. ^46^, estimating the percentile applied to each year in the baseline period on the basis of the remaining 40 years in the baseline period and then using the average across these percentiles for all years outside the baseline period. Mathematical expressions for the bootstrap resampling procedure and the calculation of global warming levels, as well as more information on the suitability of CMIP6 and ERA5 data to assess variability and extremes, are provided in Supplementary Appendix A.

### Bias correction

We bias-correct annual climate indicators using the change factor method^47^, which adds model-projected changes compared to a reference period to the corresponding reference period average of an observational dataset. For any climate indicator *C* out of the six indicators considered here, bias-corrected values are obtained as follows:

5 $${{\bar{C}}_{\rm{x},{\rm{ref}}}^{\rm{ERA5}}}+\left({C}_{\rm{x,t,m,r,s}}-{\bar{C}}_{\rm{x},{\rm{ref}},\rm{m,r,s}}\right)$$

where *C*_x,t,m,r,s_ represents the raw climate indicator output of climate model *m*’s realization *r* under scenario *s* in year *t* for grid cell *x*. ${\bar{C}}_{\rm{x},{\rm{ref}}}^{\rm{ERA5}}$ and ${\bar{C}}_{\rm{x},{\rm{ref}},m,r,s}$ represent the reference period average in ERA5 and for the climate model run, respectively. As a reference period, we use 1950–1990, during which global warming averaged +0.38 °C according to Berkeley Earth. Therefore, ${\bar{C}}_{\rm{x},{\rm{ref}},\rm{m,r,s}}$ is calculated for the 41-year global warming-level window corresponding to +0.38 °C (for the specific global warming-level windows, see Supplementary Tables 1–3). We bias-correct each annual indicator separately and, for the monthly precipitation deviation, apply the change factor method to the underlying monthly precipitation amounts. As a reference period, we use 1950–1990 because of its low influence of anthropogenic forcing and, to avoid impossible values, further impose zero lower bounds for all non-negative climate indicators and an upper bound of 365 for the number of wet days. In addition, the bias-corrected monthly precipitation deviation in some selected cases yields values that are one or two orders of magnitude above the maximum in our raw CMIP6 data. To address these outliers, we cap bias-corrected monthly precipitation deviation on the basis of the highest values observed for the raw CMIP6 data for up to +3 °C of global warming (~11.6; Supplementary Table 4), which affects only observations beyond the 99.993th percentile of our distribution.

Bias-correcting annual climate indicators ensures the highest consistency for each indicator with the ERA5 data used to estimate dose–response functions by ref. ^14^ (Supplementary Figs. 1–3). However, it can lead to inconsistencies between the different climate indicators derived from the same daily temperature or precipitation and, as outlined above, to outlier values in a few cases. As a robustness check, we apply the change factor method to the underlying daily temperature and precipitation values of an example model run instead, which increases the computational burden of bias correction considerably but leaves our conclusions unchanged (Supplementary Fig. 4).

### GDP impacts

Dose–response functions for subnational economic growth are taken from ref. ^14^, which jointly estimates the impact of all six indicators on income per capita growth. The resulting dose–response functions for each climate indicator are shown in Supplementary Fig. 7 and the underlying regression is reproduced in Supplementary Table 7, column 5. Importantly, this regression model has been subjected to comprehensive robustness checks, such as using alternative datasets and variable definitions, controlling for region-specific time trends or assessing seasonal heterogeneities^14^. Mathematically, the specification of the regression can be summarized as

6 $${g}_{\rm{i,t}}=\mathop{\sum}\limits_{C}{h}^{\rm{C}}({C}_{\rm{i,t}})+{\alpha }_{\rm{i}}+{\delta }_{\rm{t}}+{\epsilon }_{\rm{i,t}}$$

while the full model is written out in Supplementary equation (5). Here, *g*_i,t_ denotes the economic growth of ADM1-level region *i* in year *t*, measured as the first difference of the log-transformed gross regional product per capita^48^. *α*_i_, *δ*_t_ and *ϵ*_i,t_ denote fixed effects and the error term and *h*^C^ is the estimated dose–response function specific to climate indicator *C*_i,t_ where $C\in \{T,{\rm{RA}},\widetilde{T},\hat{{\rm{RD}}},{\rm{RD}},{\rm{RM}}\}$. For instance, for annual precipitation RA, the relationship with economic growth is estimated as a quadratic relationship such that

7 $${h}^{{\rm{RA}}}({\rm{RA}}_{\rm{i,t}})={\beta }_{1}^{{\rm{RA}}}{\rm{RA}}_{\rm{i,t}}+{\beta }_{2}^{{\rm{RA}}}{\rm{RA}}_{\rm{i,t}}^{2}$$

where ${\beta }_{1}^{{\rm{RA}}}$ and ${\beta }_{2}^{{\rm{RA}}}$ are the respective regression coefficients.

To calculate the impacts of climate change, we compare annual economic impacts against the average impact during the historical baseline period for the same model–realization–scenario pairing, such that our impacts represent changes from a hypothetical scenario in which climate remains constant, following previous studies^2,15^. As a baseline period for GDP impacts, we again use the +0.84 ^°^C global warming-level window for a given realization *r* of climate model *m* and RCP–SSP pair *s* for consistency with the calculation of our climate indicators. Therefore, annual impacts, in percentage of GDP, of all climate indicators combined due to shifts relative to the baseline period are calculated as follows

8 $${\delta }_{\rm{i,t}}={\rm{exp}}\left(\mathop{\sum}\limits_{C}{h}^{\rm{C}}({C}_{\rm{i,t}})-\frac{1}{41}\mathop{\sum}\limits_{k\in K}\mathop{\sum}\limits_{C}{h}^{C}({C}_{\rm{i,k}})\right)-1$$

where *K* is the 41-year model–realization–scenario-specific baseline period corresponding to +0.84 °C of global warming. Note that we exponentiate and subtract one to convert logarithmic changes to percentage of GDP, but impacts of different indicators and years are added and averaged in log scale. Individual GDP impacts of each climate indicator are obtained by using only the respective individual dose–response function in equation (8), instead of the sum across dose–response functions ∑_C_*h*^C^(*C*_i,k_). Similarly, the combined GDP impacts of variability and extremes are calculated by summing only the dose–response functions for $\widetilde{T}$, $\hat{{\rm{RD}}}$, RD and RM in equation (8). More detailed mathematical expressions for all steps in equation (8) are provided in Supplementary Appendix C.

Importantly, the model specification by ref. ^14^ features annual temperature in first-differences compared to previous years and not in absolute levels:

9 $$\begin{array}{l}{g}_{{\rm{i}},{\rm{t}}}=\ldots +{\beta}_{1}^{\rm{T}}({T}_{{\rm{i}},{\rm{t}}}-{T}_{{\rm{i}},{\rm{t}}-1})+{\beta }_{2}^{\rm{T}}({T}_{{\rm{i}},{\rm{t}}-1}-{T}_{{\rm{i}},{\rm{t}}-2})\\\qquad+{\beta }_{3}^{\rm{T}}({T}_{{\rm{i}},{\rm{t}}}-{T}_{{\rm{i}},{\rm{t}}-1}){T}_{{\rm{i}},{\rm{t}}}+{\beta }_{4}^{\rm{T}}({T}_{{\rm{i}},{\rm{t}}-1}-{T}_{{\rm{i}},{\rm{t}}-2}){T}_{{\rm{i}},{\rm{t}}-1}+\ldots\end{array}$$

To translate these regression coefficients into impact projections, we calculate cumulative impacts following ref. ^4^, such that the dose–response function for annual temperature used in equation (8) reads as follows:

10 $$\begin{array}{l}{h}^{\rm{T}}\left({T}_{{\rm{i}},{\rm{t}}},\ldots \right)=\mathop{\sum }\limits_{{\rm{j}}={\rm{k}}_{0}}^{t}\left({\beta }_{1}^{\rm{T}}({T}_{{\rm{i}},\;{\rm{j}}}-{T}_{{\rm{i}},\;{\rm{j}}-1})+{\beta }_{2}^{\rm{T}}({T}_{{\rm{i}},\;{\rm{j}}-1}-{T}_{{\rm{i}},\;{\rm{j}}-2})\right.\\\qquad\qquad\qquad\left.+{\beta }_{3}^{\rm{T}}({T}_{{\rm{i}},\;{\rm{j}}}-{T}_{{\rm{i}},\;{\rm{j}}-1}){T}_{{\rm{i}},\;{\rm{j}}}+{\beta }_{4}^{\rm{T}}({T}_{{\rm{i}},\;{\rm{j}}-1}-{T}_{{\rm{i}},\;{\rm{j}}-2}){T}_{{\rm{i}},\;{\rm{j}}-1}\right)\end{array}$$

where *k*_0_ denotes the first year in the baseline period *K*. As we discuss in Supplementary Appendix C, this procedure implicitly includes impacts of inter-annual temperature variability that persist over time.

For extreme precipitation $\hat{{\rm{RD}}}$, the dose–response function estimated by ref. ^14^ interacts extreme rainfall with the annual mean temperature *T* because the marginal impact of extreme precipitation is lower in warmer climates. Projecting this out under climate change, however, would make the strong assumption that global warming increases the resilience of countries to extreme precipitation worldwide. Because there is no evidence supporting such a positive feedback of warming and because the heterogeneity of extreme rainfall effects in ref. ^14^ is equally well-explained by the latitude of a country (see R2 and Adjusted R2 in Supplementary Table 4 of ref. ^14^), which is time-constant, we hold temperature in the interaction constant at the average level during the baseline period such that

11 $${h}^{\hat{{\rm{RD}}}}({\hat{{\rm{RD}}}}_{\rm{i,t}})={\beta }_{1}^{\hat{{\rm{RD}}}}{\hat{{\rm{RD}}}}_{\rm{i,t}}+{\beta }_{2}^{\hat{{\rm{RD}}}}{\hat{{\rm{RD}}}}_{\rm{i,t}}\frac{1}{41}\mathop{\sum}\limits_{k\in K}{T}_{\rm{i,k}}$$

When projecting damage of climate change, a core methodological choice is whether to assume that impacts affect GDP levels, such that the economy bounces back in the following year or whether to assume that a part of the damage persists and alters the long-run growth trajectory. Assuming persistence has a substantial impact on damage projections and the associated uncertainty^21,26,27^. Recent empirical analyses differ in methods and outcomes, with no consensus yet^1,2,12,21,34^. To be conservative, here we assume no persistence in implementing *δ*_i,t_, aside from any persistence implicit in the methodology by ref. ^4^, and provide further mathematical expressions and discussions of damage persistence in Supplementary Appendix C. Furthermore, by holding historical dose–response functions and baseline periods for climate indicators constant, our approach rests on the common implicit assumption that future adaptation outcomes mirror historical ones^2,4,35^, in line with the mixed evidence on macro-economic adaptation so far^2,20,21^. In addition, we follow ref. ^27^ in equating relative GDP per capita impacts with relative GDP impacts (that is, assuming that any decrease in GDP per capita is caused by a climate change-induced reduction in economic output and not by an increase in population).

### Spatial aggregation of GDP impacts

We aggregate the GDP impacts calculated via equation (8) from the subnational detail (ADM1) to the country level (ADM0) using GDP weighting. For GDP weights, we use 2010 GDP data downscaled to a 0.5° grid by ref. ^49^. To deal with 105 outlier grid cells with raw GDP data exceeding US$10^20^, we apply a ceiling at $10^10^, which is the next highest grid-cell GDP value in the dataset. Note that we hold this intracountry income distribution constant across all years and SSPs. This simplification stems from the SSPs not directly informing spatial intracountry GDP per capita distributions and also prevents our results from being driven by changes in the weighting scheme over time rather than climatic changes, which is standard practice in the literature^2^. To calculate GDP impacts at the global level, we weigh each country *i* with its share in global GDP in year *t* as per the respective SSP. Since the SSP database does not provide GDP growth trajectories for a few sovereign countries, namely Andorra, Liechtenstein, Kosovo, Nauru, North Korea, San Marino, South Sudan and Tuvalu, these economies are not represented in our damage projections for the global economy.

### GDP impact distribution

To capture dose–response function uncertainty, we draw 1,000 estimates for the dose–response function parameters ${\beta }_{1}^{{\rm{RA}}},{\beta }_{2}^{{\rm{RA}}},{\beta }_{1}^{\rm{T}},\ldots$ jointly from the multivariate Gaussian distribution estimated by ref. ^14^ (main specification, standard errors clustered at the country level). Applying each Monte Carlo draw to each of the 199 model–realization–scenario pairings provides us with 199,000 different impact projection pathways for each territory. For each model-realization–scenario pairing, we then identify the 20-year window corresponding to a global warming level of +1 °C, +1.5 °C, +2 °C, +3 °C and +4 °C, respectively, following the approach by ref. ^10^ (for the specific global warming-level windows, see Supplementary Tables 1–3. This provides us with a conditional distribution of GDP impacts for a given territory and warming level. Aside from reducing the importance of individual RCP–SSP scenarios, conditioning results on global warming levels also reduces the need to omit or down-weight ‘hot models’ in CMIP6, which project too much warming^44^. Since not all models reach all warming levels and to prevent the two large ensembles from dominating our results, we weight models inversely such that each CMIP6 model has the same sampling probability for each warming level following ref. ^18^. All summary statistics of the distribution (means, percentiles, variances and so on) are calculated using these CMIP6 model weights.

### Variance decomposition

To decompose the observed variance in our conditional global GDP impact distribution for a given warming level, we adapt the approach by ref. ^50^ based on the partitioning method by ref. ^51^. First, we carry out projections using point estimates for all dose–response function parameters (abstracting from dose–response function uncertainty) and calculate internal variability as the average across CMIP6 models of each model’s variance of global GDP impacts in a given global warming-level window. For CMIP6 models with only a single run in our analysis, this within-model variance stems from climatic differences between different scenario–years, whereas for the two large ensembles, it also includes differences between ensemble members. Correspondingly, we calculate climate model uncertainty as the variance between the mean global GDP impact of CMIP6 models for a given global warming level. Lastly, we calculate dose–response function uncertainty as the variance across dose–response function Monte Carlo draws of the mean GDP impact for each Monte Carlo draw (that is, an average across all CMIP6 models and scenario–years in the respective global warming-level window). Mathematical expressions for each variance component are provided in Supplementary Appendix D. Notably, this approach rests on the commonly made assumption that variance drivers are orthogonal, thus abstracting from interaction terms^52^. As a robustness check, we use an alternative approach by ref. ^3^ (Supplementary Appendix D), which does not affect our conclusions.

### Reporting summary

Further information on research design is available in the Nature Portfolio Reporting Summary linked to this article.

## Online content

Any methods, additional references, Nature Portfolio reporting summaries, source data, extended data, supplementary information, acknowledgements, peer review information; details of author contributions and competing interests; and statements of data and code availability are available at 10.1038/s41558-024-01990-8.

## Supplementary information

Supplementary Information Supplementary Discussion (Appendices A–F), Figs. 1–19 and Tables 1–10.

Reporting Summary

## Source data

Source Data Fig. 1 Statistical source data.

Source Data Fig. 2 Statistical source data.

Source Data Fig. 3 Statistical source data.

Source Data Fig. 4 Statistical source data.

Source Data Fig. 5 Statistical source data.

## Data Availability

CMIP6 temperature and precipitation indicators are available on the ETH Zurich CMIP6 repository^53^. CESM2 large ensemble outputs are available at https://www.earthsystemgrid.org/dataset/ucar.cgd.cesm2le.output.html. Tx5d and PDSI values from ref. ^18^ (used in Supplementary Appendix F) are available at https://github.com/ccallahan45/CallahanMankin_ExtremeHeatEconomics_2022 (ref. ^54^). The historical climate data and the economic growth data to estimate the dose–response functions from ref. ^14^ are available from ref. ^55^. ERA5 reanalysis data are available at https://www.ecmwf.int/en/forecasts/datasets/reanalysis-datasets/era5. Source data are provided with this paper. All additional data are publicly available from ref. ^56^.

## Code availability

All scripts used to conduct the analysis and create the figures are publicly available from ref. ^56^.
