# Supplementary material for: Climate damage projections beyond annual temperature
Source: Nat Clim Chang. 2024 Apr 17;14(6):592–9. doi: 10.1038/s41558-024-01990-8 (PMC11446829; doi:10.1038/s41558-024-01990-8)
Supplement: Supplementary file 1 — Supplementary Discussion (Appendices A–F), Figs. 1–19 and Tables 1–10. [file 41558_2024_1990_MOESM1_ESM.pdf]

nature climate change

Article https://doi.org/10.1038/s41558-024-01990-8

Climate damage projections beyond annual temperature

In the format provided by the authors and unedited

Contents

Appendix A - Additional information on climatic projections from CMIP6 models 2
Appendix B - Additional results and information on GDP impact projections 12
Appendix C - Additional results and information on the dose-response functions 13
Appendix D - Additional results and information on variance decompositions 22
Appendix E - Additional results on the inclusion of large ensembles 30
Appendix F - Indicative results on the inclusion of heat and drought measures 34

Appendix A - Additional information on climatic projections from CMIP6 models

Calculation of global warming level windows by climate model realization and scenario

We identify the global warming level windows for each climate model realization and RCP-SSP pair following Batibeniz et al. (2023)[1]. First, we calculate global mean surface temperature $T_{global,t}$ in a given year t for each $t \in [1850, 2100]$. Then, for each year, we calculate the centered moving average of length l:

$$\bar{T}_{global,t,l} = \begin{cases} \frac{1}{l} \sum_{j \in [t-\frac{l-1}{2}, t+\frac{l-1}{2}]} T_{global,t} & \text{if } l \text{ is odd} \\ \frac{1}{l} \sum_{j \in [t-\frac{l}{2}, t+\frac{l}{2}-1]} T_{global,t} & \text{if } l \text{ is even} \end{cases} \tag{S1}$$

Finally, the global warming level window of length l corresponding to a given temperature T_{GWL} is determined via the lowest value of t for which $\bar{T}_{global,t,l} \geq T_{GWL}$.

Since our source of dose-response functions[2] uses historical 1979–2019 data as the baseline period for climate indicators, we use $l = 41$ for the baseline global warming level window (+0.84°C, corresponding to the average of global warming between 1979–2019 according to Berkeley Earth) and the reference period for bias correction (+0.38°C, corresponding to the average of global warming between 1950-1990 global warming according to Berkeley Earth). This ensures consistency between our approach and the source of our dose-response functions, as well as between the baseline period for climate indicators and the reference period for bias correction. For the remaining global warming level windows, however, we use the standard length of $l = 20$ in line with the IPCC AR6[3].

Bootstrapping procedure for extreme precipitation

Climate indicators that use percentile-based thresholds can exhibit artificial jumps at the end of the baseline period for which the percentiles are calculated[4, 5]. To avoid this, we calculate percentile-based thresholds using the bootstrapping procedure developed by Zhang et al. (2005)[4]: Let the rainfall in grid cell x on day d in year k for a given climate model realization and scenario be denoted as $R_{x,d,k}$ and let the set of 41 years in the +0.84°C global warming level window for this specific model realization and scenario be denoted as K (for an overview of global warming level windows, see Tables S1–S3). Then for each $k \in K$, we carry out the following steps:

- We drop the year k from the sample of all 41 years in the baseline period K and replace it with another year randomly sampled from K, such that this year features twice in the resulting sample.
- We calculate the 99.9th percentile for this modified sample, which we denote here as $R_{x,99p9,K\setminus k}$.
- We then apply this threshold $R_{x,99p9,K\setminus k}$ to the excluded year k to calculate extreme precipitation for year k:

$$\hat{RD}_{x,k} = \sum_{d=1}^{365} R_{x,d,k} \times I(R_{x,d,k} > R_{x,99p9,K\setminus k}) \tag{S2}$$

Repeating this for each $k \in K$ provides 41 different percentile estimates $R_{x,99p9,K\setminus k}$. For any year $t \notin K$ outside the baseline period, we then use the average across these 41 percentiles as the threshold:

$$R_{x,99p9,base} = \frac{1}{41} \sum_{k \in K} R_{x,99p9,K\setminus k} \tag{S3}$$

such that extreme rainfall for all years $t \notin K$ outside the baseline period is calculated as follows:

$$\hat{RD}_{x,t} = \sum_{d=1}^{365} R_{x,d,t} \times I(R_{x,d,t} > R_{x,99p9,base}) \tag{S4}$$

For more details and context on this procedure, we refer the reader to Zhang et al. (2005)[4].

Suitability of CMIP6 and ERA5 data for projecting variability and extremes

Climate models participating in CMIP6 are widely regarded as one of the most comprehensive and reliable sources for information on future climatic shifts[3]. The chapter on weather and climatic extremes in the IPCC AR6 has assessed the capability of CMIP6 models to represent weather and climate extremes in comprehensive detail and concluded that "there is high confidence that climate models can reproduce the mean state and overall warming of temperature extremes observed globally and in most regions, although the magnitude of the trends may differ" (Seneviratne et al., 2021[6], p. 1552) and

"there is high confidence in the ability of models to capture the large-scale spatial distribution of precipitation extremes over land." (Seneviratne et al., 2021[6], p. 1562) Among key shortcomings, CMIP6 models exhibit a warm (cold) bias in hot (cold) temperature extremes in many regions, and some key land forcings on temperature extremes (i.e., deforestation and irrigation) need to be better represented. Regarding heavy precipitation events, the spatial resolution of these models can limit their representation of precipitation extremes in regions with heterogeneous topography, which can motivate the use of regional high-resolution models[7, 8, 9, 10], and region-specific biases with different signs and magnitudes remain[11, 12]. More generally, there are concerns around how accurately models represent certain physical processes and feedbacks[13, 14, 15, 16].

Nevertheless, CMIP6 model outputs, especially when bias-corrected, remain a state-of-the-art source for future shifts in temperature and precipitation patterns, and they have heavily informed the synthesis of region-specific projections of climatic extremes in the respective chapter of IPCC AR6[6]. As extreme events are difficult to study merely based on observational data due to their rare nature, outputs by global climate models have also been used extensively to analyze historical developments[1, 17, 18, 19, 20, 21]. Such historical analyses using CMIP6 data have covered various extremes, such as heat, drought, extreme precipitation, and compound events. Historical simulations from CMIP6 models have also informed backward-looking assessments of heat-related impacts on past economic growth[22]. In addition, temperature output from previous CMIP generations has been used extensively to project the economic effects of climate change[23, 24, 25, 26, 27].

Therefore, CMIP6 models remain the most comprehensive and established option available for creating consistent projections of temperature and precipitation patterns around the globe, as required for this study. For the six climate indicators under consideration here, we note that the +0.84°C global warming level window of the respective bias-corrected CMIP6 model runs accurately reproduce the historical 1979–2019 distribution of indicators in ERA5 at the subnational region level (see Figure S1), which is the geographic level used for projecting damages. Climate models produce somewhat higher amount of extreme precipitation and, for some regions, wet days. At the same time, they exhibit less pronounced tails for annual precipitation and, for most models, extreme precipitation (see Figure S2). Assessing the distribution separately for each CMIP6 model in our sample does not reveal any striking differences across models except for a very pronounced tail of extreme precipitation in KACE-1-0-G and HadGEM3-GC31-LL. In addition, correlational patterns between the climate indicators in our CMIP6 data are strikingly similar to the patterns found in historical ERA5 data (see Figure S3).

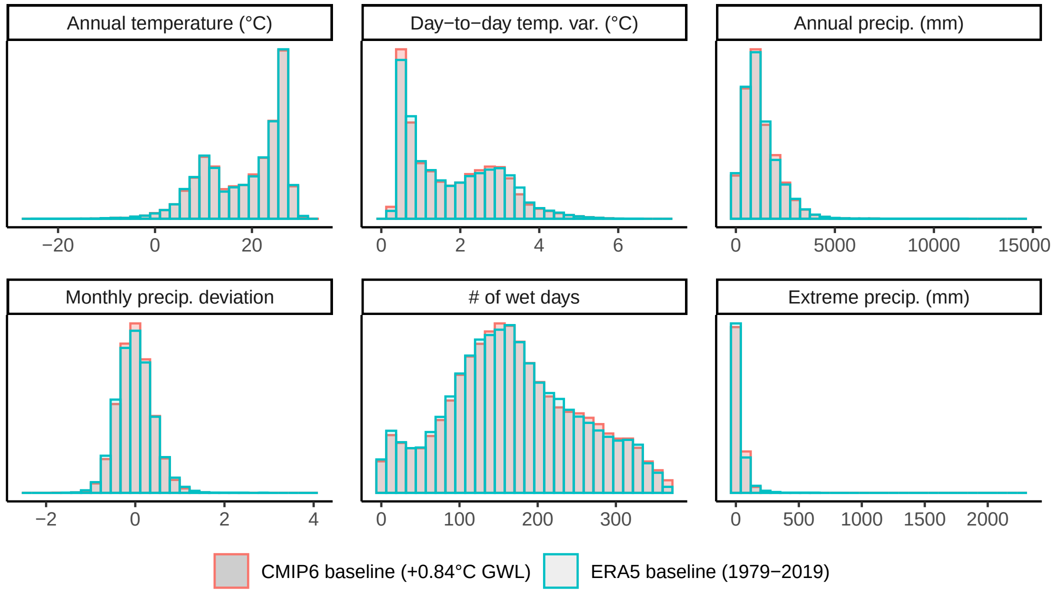

Figure S1. Distribution of ADM1-level climate indicators in the ERA5 1979–2019 baseline period and the corresponding +0.84°C global warming level window for bias-corrected CMIP6 models. For the two large ensembles, only the r1i1p1f1 model run is displayed to reduce computational requirements and prevent the large ensembles from dominating the distribution shown.

Regarding the suitability of ERA5 to capture the climate indicators considered here and inform dose-response function estimations, we refer the reader to Kotz et al. (2022)[2], Kotz et al. (2021)[28] and, for the heat measure used in Appendix F, Callahan and Mankin (2022)[22]. Importantly, these studies, which estimated the dose-response functions for the climate indicators considered here based on ERA5, have reproduced their findings using alternative datasets (i.e., EWEMBI and WFDEI for temperature and, for precipitation, MSWEP and the Princeton Global Meteorological Forcing, which involve gauge or bias correction). More generally, ERA5 has been found to capture extreme temperatures sufficiently well in region-specific

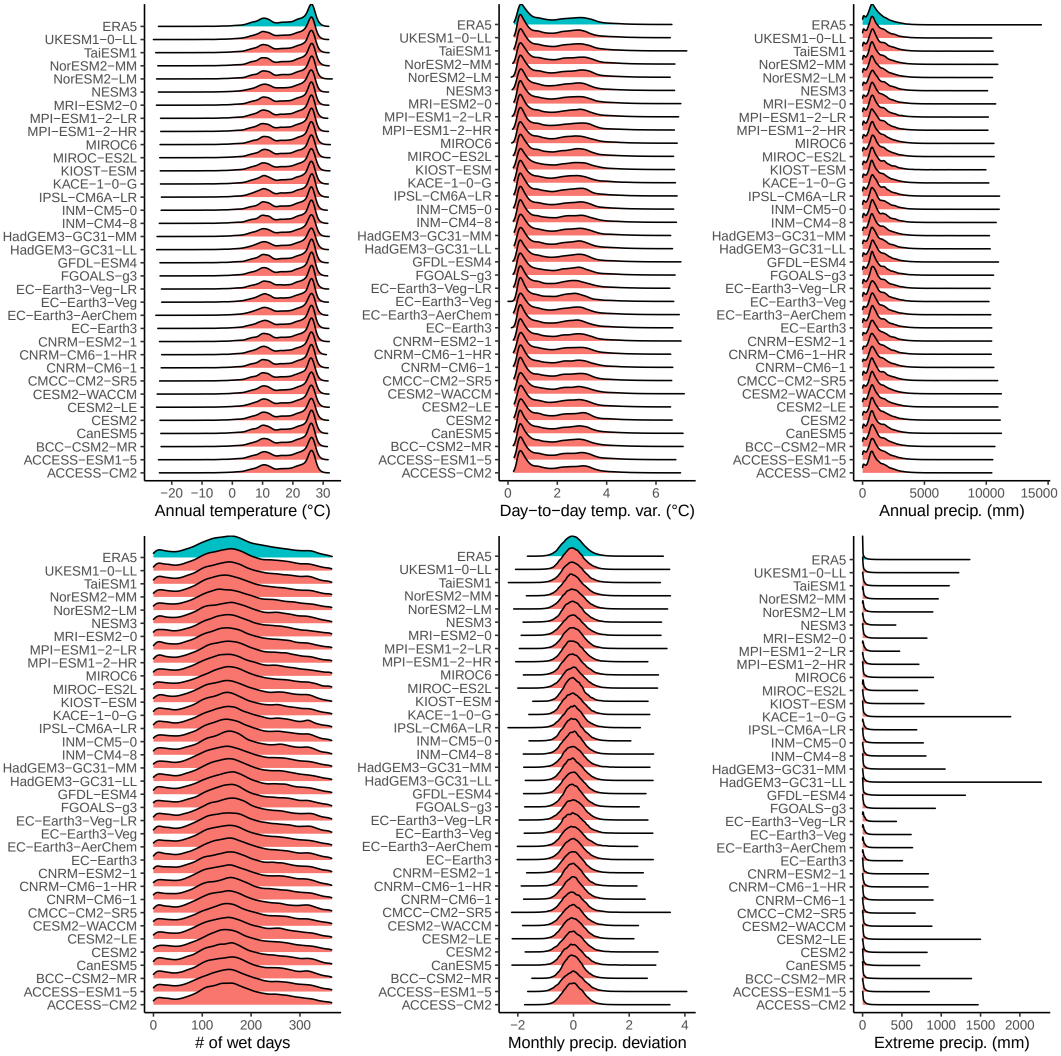

Figure S2. Distribution of ADM1-level climate indicators in the ERA5 1979–2019 baseline period and the corresponding +0.84°C global warming level window for bias-corrected CMIP6 models, displayed separately by model. For the two large ensembles, only the r1i1p1f1 model run is shown to reduce computational requirements.

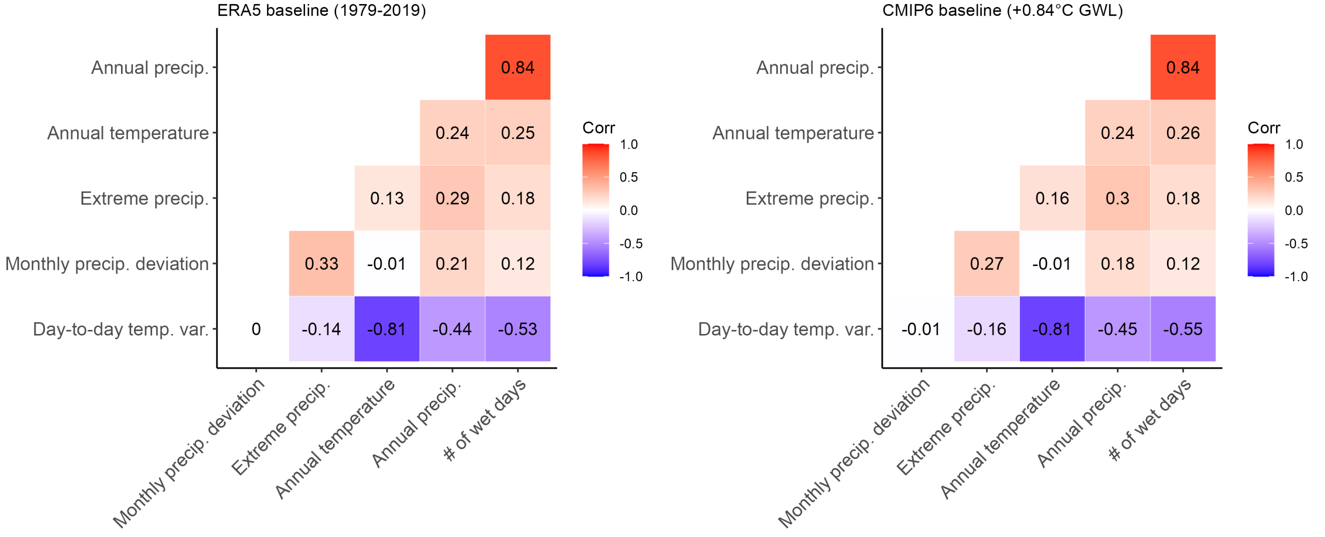

Figure S3. Pearson correlation coefficients of ADM1-level climate indicators within the ERA5 1979–2019 baseline period and within the corresponding +0.84°C global warming level window for bias-corrected CMIP6 models. For the two large ensembles, only the r1i1p1f1 model run is considered to reduce computational requirements and prevent the large ensembles from dominating the correlation coefficients calculated.

studies[29, 30] and to outperform other reanalysis products regarding (extreme) precipitation[31, 32]. Heavy precipitation (number of wet days) can be underestimated (overestimated) in certain regions[33, 34], although some of them (e.g., West Africa) are not represented or have a very limited presence in the sample used by Kotz et al. (2022)[2] to estimate the dose-response functions deployed here. Therefore, measurement errors in the reanalysis data could affect our damage projections through a general attenuation bias towards zero, particularly given the presence of high-resolution fixed effects[35], or away from zero for indicators with a more systematic bias in ERA5. However, Kotz et al. (2022)[2] report that estimating the same regression model using MSWEP—which performed best in a systematic evaluation of 26 precipitation datasets for the United States[32] and in an evaluation of 22 precipitation datasets using global gauge observations[36]— instead of ERA5 yields comparable estimates for the number of wet days and coefficients of higher magnitude for extreme precipitation (see their Table S9). Therefore, our dose-response function for extreme precipitation could be (too) conservative if MSWEP captures extreme precipitation consistently better than ERA5.

For more general information on ERA5 and its performance, we refer the reader to Hersbach et al. (2020)[37] and to the respective discussion of ERA5 and reanalysis data more generally in the IPCC AR6 Working Group I report[3].

	GCM	Scenario	Ensemble member	+0.38°C	+0.84°C	+1°C	+1.5°C	+2°C	+3°C	+4°C
1	ACCESS-CM2	ssp126	r1i1p1f1	1973-2013	1990-2030	2006-2025	2018-2037	2033-2052		
2	ACCESS-CM2	ssp370	r1i1p1f1	1973-2013	1990-2030	2006-2025	2018-2037	2030-2049	2053-2072	2073-2092
3	ACCESS-ESM1-5	ssp126	r1i1p1f1	1972-2012	1988-2028	2004-2023	2021-2040	2064-2083		
4	ACCESS-ESM1-5	ssp370	r1i1p1f1	1972-2012	1989-2029	2004-2023	2024-2043	2039-2058	2060-2079	
5	BCC-CSM2-MR	ssp126	r1i1p1f1	1971-2011	1992-2032	2009-2028	2032-2051			
6	BCC-CSM2-MR	ssp370	r1i1p1f1	1971-2011	1992-2032	2008-2027	2023-2042	2037-2056	2065-2084	
7	CanESM5	ssp119	r1i1p1f1	1950-1990	1973-2013	1990-2009	2003-2022	2015-2034		
8	CanESM5	ssp126	r1i1p1f1	1950-1990	1973-2013	1990-2009	2004-2023	2017-2036		
9	CanESM5	ssp370	r1i1p1f1	1950-1990	1973-2013	1990-2009	2004-2023	2014-2033	2034-2053	2050-2069
10	CESM2	ssp126	r1i1p1f1	1965-2005	1984-2024	2000-2019	2016-2035	2032-2051		
11	CESM2-LE	ssp370	Large ensemble	Varying	Varying	Varying	Varying	Varying	Varying	Varying
12	CESM2-WACCM	ssp370	r1i1p1f1	1962-2002	1983-2023	1997-2016	2019-2038	2032-2051	2054-2073	2076-2095
13	CMCC-CM2-SR5	ssp126	r1i1p1f1	1945-1985	1974-2014	1993-2012	2014-2033	2029-2048		
14	CMCC-CM2-SR5	ssp370	r1i1p1f1	1945-1985	1974-2014	1993-2012	2016-2035	2030-2049	2054-2073	2078-2097
15	CNRM-CM6-1	ssp126	r1i1p1f2	1961-2001	1984-2024	2003-2022	2018-2037	2050-2069		
16	CNRM-CM6-1	ssp370	r1i1p1f2	1961-2001	1985-2025	2004-2023	2023-2042	2036-2055	2057-2076	2074-2093
17	CNRM-CM6-1-HR	ssp126	r1i1p1f2	1917-1957	1974-2014	1994-2013	2008-2027	2021-2040		
18	CNRM-ESM2-1	ssp119	r1i1p1f2	1971-2011	1991-2031	2007-2026	2058-2077			
19	CNRM-ESM2-1	ssp126	r1i1p1f2	1971-2011	1991-2031	2006-2025	2038-2057			
20	CNRM-ESM2-1	ssp370	r1i1p1f2	1971-2011	1992-2032	2009-2028	2027-2046	2043-2062	2063-2082	2080-2099
21	EC-Earth3	ssp119	r4i1p1f1	1909-1949	1966-2006	1985-2004	2008-2027	2035-2054		
22	EC-Earth3	ssp126	r1i1p1f1	1972-2012	1985-2025	1999-2018	2013-2032	2034-2053		
23	EC-Earth3	ssp370	r1i1p1f1	1972-2012	1985-2025	1999-2018	2013-2032	2029-2048	2054-2073	2075-2094
24	EC-Earth3-AerChem	ssp370	r1i1p1f1	1980-2020	1993-2033	2009-2028	2023-2042	2038-2057	2057-2076	2074-2093
25	EC-Earth3-Veg	ssp119	r1i1p1f1	1909-1949	1965-2005	1984-2003	2002-2021	2020-2039		
26	EC-Earth3-Veg	ssp126	r1i1p1f1	1909-1949	1965-2005	1984-2003	2003-2022	2020-2039		
27	EC-Earth3-Veg	ssp370	r1i1p1f1	1909-1949	1965-2005	1984-2003	2002-2021	2023-2042	2048-2067	2067-2086
28	EC-Earth3-Veg-LR	ssp119	r1i1p1f1	1951-1991	1981-2021	1997-2016	2020-2039			
29	EC-Earth3-Veg-LR	ssp126	r1i1p1f1	1951-1991	1981-2021	1998-2017	2021-2040			
30	EC-Earth3-Veg-LR	ssp370	r1i1p1f1	1951-1991	1981-2021	1998-2017	2019-2038	2036-2055	2057-2076	2078-2097
31	FGOALS-g3	ssp119	r1i1p1f1	1956-1996	1982-2022	1997-2016				
32	FGOALS-g3	ssp126	r1i1p1f1	1956-1996	1982-2022	1996-2015				
33	FGOALS-g3	ssp370	r1i1p1f1	1956-1996	1982-2022	1997-2016	2018-2037	2037-2056	2075-2094	
34	GFDL-ESM4	ssp119	r1i1p1f1	1973-2013	1994-2034	2011-2030				
35	GFDL-ESM4	ssp126	r1i1p1f1	1973-2013	1994-2034	2011-2030				
36	GFDL-ESM4	ssp370	r1i1p1f1	1973-2013	1995-2035	2012-2031	2032-2051	2048-2067	2074-2093	
37	HadGEM3-GC31-LL	ssp126	r1i1p1f3	1974-2014	1987-2027	2000-2019	2012-2031	2028-2047		
38	HadGEM3-GC31-MM	ssp126	r1i1p1f3	1973-2013	1991-2031	2006-2025	2019-2038	2031-2050		
39	INM-CM4-8	ssp126	r1i1p1f1	1955-1995	1986-2026	2005-2024	2041-2060			
40	INM-CM4-8	ssp370	r1i1p1f1	1955-1995	1987-2027	2007-2026	2026-2045	2043-2062	2074-2093	
41	INM-CM5-0	ssp126	r1i1p1f1	1961-2001	1988-2028	2007-2026	2027-2046			
42	INM-CM5-0	ssp370	r1i1p1f1	1961-2001	1988-2028	2006-2025	2023-2042	2041-2060	2075-2094	
43	IPSL-CM6A-LR	ssp119	r1i1p1f1	1931-1971	1975-2015	1993-2012	2009-2028	2028-2047		
44	IPSL-CM6A-LR	ssp126	r1i1p1f1	1931-1971	1975-2015	1993-2012	2010-2029	2029-2048		
45	IPSL-CM6A-LR	ssp370	r1i1p1f1	1931-1971	1975-2015	1993-2012	2010-2029	2025-2044	2046-2065	2067-2086
46	KACE-1-0-G	ssp126	r1i1p1f1	1934-1974	1974-2014	1991-2010	2005-2024	2015-2034		
47	KACE-1-0-G	ssp370	r1i1p1f1	1934-1974	1974-2014	1991-2010	2005-2024	2015-2034	2037-2056	2063-2082
48	KIOST-ESM	ssp126	r1i1p1f1	1946-1986	1973-2013	1993-2012	2011-2030			
49	MIROC-ES2L	ssp119	r1i1p1f2	1972-2012	1994-2034	2012-2031				
50	MIROC-ES2L	ssp126	r1i1p1f2	1972-2012	1994-2034	2010-2029	2032-2051			
51	MIROC-ES2L	ssp370	r1i1p1f2	1972-2012	1994-2034	2010-2029	2030-2049	2046-2065	2075-2094	
52	MIROC6	ssp119	r1i1p1f1	1972-2012	1997-2037	2016-2035				
53	MIROC6	ssp126	r1i1p1f1	1972-2012	1997-2037	2016-2035	2054-2073			
54	MIROC6	ssp370	r1i1p1f1	1972-2012	1997-2037	2017-2036	2034-2053	2050-2069		
55	MPI-ESM1-2-HR	ssp126	r1i1p1f1	1950-1990	1984-2024	2002-2021	2032-2051			
56	MPI-ESM1-2-HR	ssp370	r1i1p1f1	1950-1990	1985-2025	2001-2020	2025-2044	2041-2060	2072-2091	
57	MPI-ESM1-2-LR	ssp126	r1i1p1f1	1957-1997	1983-2023	2002-2021	2033-2052			
58	MPI-ESM1-2-LR	ssp370	Large ensemble	Varying	Varying	Varying	Varying	Varying	Varying	
59	MRI-ESM2-0	ssp119	r1i1p1f1	1969-2009	1989-2029	2005-2024	2021-2040			
60	MRI-ESM2-0	ssp126	r1i1p1f1	1969-2009	1989-2029	2005-2024	2020-2039			
61	MRI-ESM2-0	ssp370	r1i1p1f1	1969-2009	1989-2029	2005-2024	2022-2041	2036-2055	2064-2083	
62	NESM3	ssp126	r1i1p1f1	1969-2009	1985-2025	2000-2019	2012-2031	2040-2059		
63	NorESM2-LM	ssp126	r1i1p1f1	1977-2017	1998-2038	2018-2037				
64	NorESM2-LM	ssp370	r1i1p1f1	1977-2017	2000-2040	2023-2042	2042-2061	2060-2079		
65	NorESM2-MM	ssp126	r1i1p1f1	1978-2018	2000-2040	2013-2032				
66	NorESM2-MM	ssp370	r1i1p1f1	1978-2018	2002-2042	2022-2041	2037-2056	2053-2072	2081-2100	
67	TaiESM1	ssp126	r1i1p1f1	1981-2021	1993-2033	2008-2027	2018-2037	2031-2050		
68	TaiESM1	ssp370	r1i1p1f1	1981-2021	1996-2036	2010-2029	2024-2043	2034-2053	2052-2071	2072-2091
69	UKESM1-0-LL	ssp119	r1i1p1f2	1977-2017	1988-2028	2004-2023	2015-2034	2030-2049		
70	UKESM1-0-LL	ssp126	r1i1p1f2	1977-2017	1988-2028	2003-2022	2014-2033	2027-2046		
71	UKESM1-0-LL	ssp370	r1i1p1f2	1977-2017	1988-2028	2004-2023	2013-2032	2022-2041	2041-2060	2060-2079

Table S1. Models, scenarios and ensemble members used for climatic projections with the respective global warming level windows. Details on the large ensemble members for CESM2-LE and MPI-ESM1-2-LR under SSP3-7.0 are provided in Table S2 and Table S3.

	GCM	Scenario	Ensemble member	+0.38°C	+0.84°C	+1°C	+1.5°C	+2°C	+3°C
1	MPI-ESM1-2-LR	ssp370	r1i1p1f1	1957-1997	1984-2024	2004-2023	2026-2045	2043-2062	2069-2088
2	MPI-ESM1-2-LR	ssp370	r2i1p1f1	1955-1995	1985-2025	2005-2024	2024-2043	2041-2060	2073-2092
3	MPI-ESM1-2-LR	ssp370	r3i1p1f1	1955-1995	1983-2023	2003-2022	2025-2044	2041-2060	2071-2090
4	MPI-ESM1-2-LR	ssp370	r4i1p1f1	1948-1988	1980-2020	1998-2017	2024-2043	2042-2061	2071-2090
5	MPI-ESM1-2-LR	ssp370	r5i1p1f1	1956-1996	1984-2024	2000-2019	2025-2044	2041-2060	2070-2089
6	MPI-ESM1-2-LR	ssp370	r6i1p1f1	1945-1985	1979-2019	1995-2014	2024-2043	2039-2058	2068-2087
7	MPI-ESM1-2-LR	ssp370	r7i1p1f1	1950-1990	1983-2023	2001-2020	2026-2045	2041-2060	2071-2090
8	MPI-ESM1-2-LR	ssp370	r8i1p1f1	1951-1991	1985-2025	2001-2020	2024-2043	2042-2061	2072-2091
9	MPI-ESM1-2-LR	ssp370	r9i1p1f1	1950-1990	1982-2022	1999-2018	2024-2043	2041-2060	2072-2091
10	MPI-ESM1-2-LR	ssp370	r10i1p1f1	1950-1990	1980-2020	1995-2014	2025-2044	2039-2058	2069-2088
11	MPI-ESM1-2-LR	ssp370	r11i1p1f1	1947-1987	1981-2021	1999-2018	2023-2042	2039-2058	2069-2088
12	MPI-ESM1-2-LR	ssp370	r12i1p1f1	1957-1997	1984-2024	2000-2019	2026-2045	2042-2061	2072-2091
13	MPI-ESM1-2-LR	ssp370	r13i1p1f1	1950-1990	1985-2025	2001-2020	2023-2042	2040-2059	2071-2090
14	MPI-ESM1-2-LR	ssp370	r14i1p1f1	1962-2002	1987-2027	2005-2024	2025-2044	2043-2062	2073-2092
15	MPI-ESM1-2-LR	ssp370	r15i1p1f1	1951-1991	1983-2023	2000-2019	2025-2044	2040-2059	2069-2088
16	MPI-ESM1-2-LR	ssp370	r16i1p1f1	1950-1990	1982-2022	2000-2019	2025-2044	2040-2059	2070-2089
17	MPI-ESM1-2-LR	ssp370	r17i1p1f1	1954-1994	1982-2022	1998-2017	2026-2045	2041-2060	2071-2090
18	MPI-ESM1-2-LR	ssp370	r18i1p1f1	1954-1994	1982-2022	1999-2018	2024-2043	2040-2059	2071-2090
19	MPI-ESM1-2-LR	ssp370	r19i1p1f1	1945-1985	1983-2023	2000-2019	2024-2043	2040-2059	2071-2090
20	MPI-ESM1-2-LR	ssp370	r20i1p1f1	1958-1998	1988-2028	2009-2028	2029-2048	2043-2062	2074-2093
21	MPI-ESM1-2-LR	ssp370	r21i1p1f1	1942-1982	1978-2018	1997-2016	2023-2042	2040-2059	2070-2089
22	MPI-ESM1-2-LR	ssp370	r22i1p1f1	1947-1987	1981-2021	1999-2018	2023-2042	2040-2059	2072-2091
23	MPI-ESM1-2-LR	ssp370	r23i1p1f1	1958-1998	1984-2024	2003-2022	2026-2045	2043-2062	2071-2090
24	MPI-ESM1-2-LR	ssp370	r24i1p1f1	1945-1985	1976-2016	1995-2014	2022-2041	2040-2059	2070-2089
25	MPI-ESM1-2-LR	ssp370	r25i1p1f1	1954-1994	1982-2022	1999-2018	2024-2043	2043-2062	2072-2091
26	MPI-ESM1-2-LR	ssp370	r26i1p1f1	1951-1991	1980-2020	2001-2020	2025-2044	2040-2059	2070-2089
27	MPI-ESM1-2-LR	ssp370	r27i1p1f1	1946-1986	1983-2023	2000-2019	2026-2045	2042-2061	2070-2089
28	MPI-ESM1-2-LR	ssp370	r28i1p1f1	1958-1998	1982-2022	1998-2017	2023-2042	2042-2061	2071-2090
29	MPI-ESM1-2-LR	ssp370	r29i1p1f1	1943-1983	1982-2022	2001-2020	2022-2041	2041-2060	2069-2088
30	MPI-ESM1-2-LR	ssp370	r30i1p1f1	1950-1990	1985-2025	2001-2020	2027-2046	2041-2060	2070-2089

Table S2. MPI-ESM1-2-LR large ensemble members used for SSP3-7.0. The column for +4°C is omitted because no model run reaches that warming level.

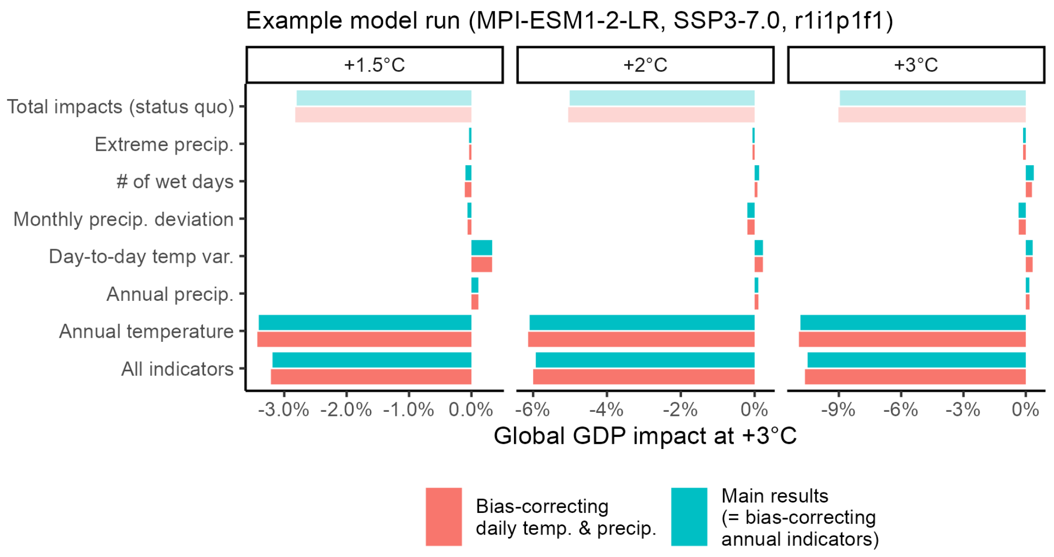

Figure S4. Global GDP impacts for bias-correcting daily temperature and precipitation, instead of annual climate indicators, for an example model run (MPI-ESM1-2-LR, SSP3-7.0, r1i1p1f1). Bars denote the distribution mean excluding dose-response function uncertainty (i.e., using point estimates for dose-response function parameters) to reduce computational requirements.

	GCM	Scenario	Ensemble member	+0.38°C	+0.84°C	+1°C	+1.5°C	+2°C	+3°C	+4°C
1	CESM2-LE	ssp370	r1i1p1f1	1962-2002	1982-2022	1998-2017	2016-2035	2033-2052	2058-2077	2078-2097
2	CESM2-LE	ssp370	r2i1p1f1	1969-2009	1988-2028	2003-2022	2023-2042	2035-2054	2060-2079	2079-2098
3	CESM2-LE	ssp370	r3i1p1f1	1967-2007	1987-2027	2001-2020	2022-2041	2037-2056	2059-2078	2079-2098
4	CESM2-LE	ssp370	r4i1p1f1	1971-2011	1990-2030	2007-2026	2024-2043	2035-2054	2058-2077	2080-2099
5	CESM2-LE	ssp370	r5i1p1f1	1959-1999	1981-2021	1995-2014	2018-2037	2032-2051	2055-2074	2077-2096
6	CESM2-LE	ssp370	r6i1p1f1	1966-2006	1985-2025	1999-2018	2021-2040	2034-2053	2058-2077	2078-2097
7	CESM2-LE	ssp370	r7i1p1f1	1961-2001	1985-2025	2001-2020	2020-2039	2033-2052	2056-2075	2077-2096
8	CESM2-LE	ssp370	r8i1p1f1	1971-2011	1988-2028	2003-2022	2020-2039	2032-2051	2059-2078	2080-2099
9	CESM2-LE	ssp370	r9i1p1f1	1956-1996	1985-2025	2001-2020	2020-2039	2033-2052	2058-2077	2078-2097
10	CESM2-LE	ssp370	r10i1p1f1	1966-2006	1986-2026	1998-2017	2019-2038	2032-2051	2059-2078	2079-2098
11	CESM2-LE	ssp370	r11i1p1f1	1963-2003	1985-2025	2000-2019	2021-2040	2032-2051	2056-2075	2077-2096
12	CESM2-LE	ssp370	r12i1p1f1	1966-2006	1986-2026	2000-2019	2020-2039	2033-2052	2058-2077	2077-2096
13	CESM2-LE	ssp370	r13i1p1f1	1971-2011	1988-2028	2003-2022	2021-2040	2033-2052	2057-2076	2078-2097
14	CESM2-LE	ssp370	r14i1p1f1	1969-2009	1987-2027	2002-2021	2024-2043	2038-2057	2060-2079	2081-2100
15	CESM2-LE	ssp370	r15i1p1f1	1962-2002	1982-2022	1997-2016	2018-2037	2033-2052	2056-2075	2078-2097
16	CESM2-LE	ssp370	r16i1p1f1	1970-2010	1988-2028	2005-2024	2022-2041	2035-2054	2058-2077	2079-2098
17	CESM2-LE	ssp370	r17i1p1f1	1964-2004	1986-2026	2003-2022	2021-2040	2033-2052	2058-2077	2079-2098
18	CESM2-LE	ssp370	r18i1p1f1	1969-2009	1987-2027	2005-2024	2023-2042	2037-2056	2062-2081	2080-2099
19	CESM2-LE	ssp370	r19i1p1f1	1968-2008	1986-2026	2000-2019	2020-2039	2033-2052	2060-2079	2078-2097
20	CESM2-LE	ssp370	r20i1p1f1	1961-2001	1984-2024	2000-2019	2021-2040	2035-2054	2058-2077	2078-2097
21	CESM2-LE	ssp370	r21i1p1f1	1966-2006	1987-2027	2004-2023	2020-2039	2035-2054	2059-2078	2079-2098
22	CESM2-LE	ssp370	r22i1p1f1	1966-2006	1987-2027	2005-2024	2022-2041	2035-2054	2060-2079	2080-2099
23	CESM2-LE	ssp370	r23i1p1f1	1967-2007	1986-2026	2002-2021	2016-2035	2031-2050	2057-2076	2079-2098
24	CESM2-LE	ssp370	r24i1p1f1	1967-2007	1991-2031	2008-2027	2025-2044	2036-2055	2058-2077	2078-2097
25	CESM2-LE	ssp370	r25i1p1f1	1969-2009	1990-2030	2006-2025	2021-2040	2034-2053	2058-2077	2079-2098
26	CESM2-LE	ssp370	r26i1p1f1	1973-2013	1991-2031	2005-2024	2022-2041	2036-2055	2060-2079	2079-2098
27	CESM2-LE	ssp370	r27i1p1f1	1965-2005	1987-2027	2003-2022	2022-2041	2035-2054	2057-2076	2078-2097
28	CESM2-LE	ssp370	r28i1p1f1	1969-2009	1987-2027	2001-2020	2022-2041	2036-2055	2061-2080	2080-2099
29	CESM2-LE	ssp370	r29i1p1f1	1965-2005	1985-2025	2002-2021	2020-2039	2034-2053	2058-2077	2076-2095
30	CESM2-LE	ssp370	r30i1p1f1	1963-2003	1985-2025	2002-2021	2017-2036	2030-2049	2057-2076	2077-2096
31	CESM2-LE	ssp370	r31i1p1f1	1969-2009	1990-2030	2008-2027	2022-2041	2033-2052	2058-2077	2078-2097
32	CESM2-LE	ssp370	r32i1p1f1	1964-2004	1985-2025	2000-2019	2020-2039	2032-2051	2055-2074	2077-2096
33	CESM2-LE	ssp370	r33i1p1f1	1967-2007	1985-2025	1999-2018	2017-2036	2029-2048	2056-2075	2076-2095
34	CESM2-LE	ssp370	r34i1p1f1	1971-2011	1987-2027	2001-2020	2024-2043	2038-2057	2062-2081	2081-2100
35	CESM2-LE	ssp370	r35i1p1f1	1969-2009	1988-2028	2004-2023	2021-2040	2036-2055	2060-2079	2080-2099
36	CESM2-LE	ssp370	r36i1p1f1	1964-2004	1986-2026	2000-2019	2023-2042	2039-2058	2063-2082	2081-2100
37	CESM2-LE	ssp370	r37i1p1f1	1961-2001	1986-2026	2004-2023	2021-2040	2032-2051	2056-2075	2076-2095
38	CESM2-LE	ssp370	r38i1p1f1	1967-2007	1986-2026	2001-2020	2021-2040	2035-2054	2060-2079	2078-2097
39	CESM2-LE	ssp370	r39i1p1f1	1971-2011	1988-2028	2004-2023	2021-2040	2035-2054	2059-2078	2079-2098
40	CESM2-LE	ssp370	r40i1p1f1	1971-2011	1987-2027	2000-2019	2020-2039	2034-2053	2059-2078	2079-2098
41	CESM2-LE	ssp370	r41i1p1f1	1968-2008	1989-2029	2007-2026	2024-2043	2037-2056	2060-2079	2079-2098
42	CESM2-LE	ssp370	r42i1p1f1	1962-2002	1985-2025	2005-2024	2021-2040	2034-2053	2059-2078	2079-2098
43	CESM2-LE	ssp370	r43i1p1f1	1973-2013	1995-2035	2012-2031	2025-2044	2036-2055	2060-2079	2080-2099
44	CESM2-LE	ssp370	r44i1p1f1	1969-2009	1988-2028	2004-2023	2023-2042	2036-2055	2057-2076	2080-2099
45	CESM2-LE	ssp370	r45i1p1f1	1966-2006	1988-2028	2006-2025	2018-2037	2032-2051	2058-2077	2078-2097
46	CESM2-LE	ssp370	r46i1p1f1	1974-2014	1993-2033	2007-2026	2025-2044	2037-2056	2063-2082	
47	CESM2-LE	ssp370	r47i1p1f1	1969-2009	1991-2031	2008-2027	2022-2041	2036-2055	2059-2078	2079-2098
48	CESM2-LE	ssp370	r48i1p1f1	1973-2013	1993-2033	2009-2028	2025-2044	2036-2055	2056-2075	2076-2095
49	CESM2-LE	ssp370	r49i1p1f1	1968-2008	1990-2030	2006-2025	2024-2043	2036-2055	2060-2079	2079-2098
50	CESM2-LE	ssp370	r50i1p1f1	1968-2008	1989-2029	2005-2024	2021-2040	2032-2051	2059-2078	2079-2098
51	CESM2-LE	ssp370	r51i1p1f1	1969-2009	1988-2028	2003-2022	2019-2038	2033-2052	2056-2075	2078-2097
52	CESM2-LE	ssp370	r52i1p1f1	1957-1997	1979-2019	1995-2014	2018-2037	2031-2050	2057-2076	2075-2094
53	CESM2-LE	ssp370	r53i1p1f1	1962-2002	1984-2024	2002-2021	2020-2039	2033-2052	2054-2073	2077-2096
54	CESM2-LE	ssp370	r54i1p1f1	1966-2006	1986-2026	2002-2021	2022-2041	2038-2057	2061-2080	2080-2099
55	CESM2-LE	ssp370	r55i1p1f1	1965-2005	1986-2026	2000-2019	2022-2041	2034-2053	2057-2076	2078-2097
56	CESM2-LE	ssp370	r56i1p1f1	1967-2007	1987-2027	2002-2021	2019-2038	2032-2051	2056-2075	2077-2096
57	CESM2-LE	ssp370	r57i1p1f1	1965-2005	1985-2025	1999-2018	2020-2039	2034-2053	2059-2078	2078-2097
58	CESM2-LE	ssp370	r58i1p1f1	1966-2006	1984-2024	1999-2018	2018-2037	2035-2054	2060-2079	2078-2097
59	CESM2-LE	ssp370	r59i1p1f1	1966-2006	1985-2025	1999-2018	2023-2042	2036-2055	2057-2076	2079-2098
60	CESM2-LE	ssp370	r60i1p1f1	1963-2003	1983-2023	1998-2017	2019-2038	2033-2052	2059-2078	2077-2096
61	CESM2-LE	ssp370	r61i1p1f1	1967-2007	1989-2029	2006-2025	2022-2041	2037-2056	2058-2077	2079-2098
62	CESM2-LE	ssp370	r62i1p1f1	1959-1999	1985-2025	2002-2021	2018-2037	2033-2052	2058-2077	2078-2097
63	CESM2-LE	ssp370	r63i1p1f1	1966-2006	1985-2025	1999-2018	2021-2040	2034-2053	2059-2078	2079-2098
64	CESM2-LE	ssp370	r64i1p1f1	1962-2002	1985-2025	2003-2022	2020-2039	2033-2052	2056-2075	2076-2095
65	CESM2-LE	ssp370	r65i1p1f1	1956-1996	1983-2023	2001-2020	2018-2037	2031-2050	2054-2073	2077-2096
66	CESM2-LE	ssp370	r66i1p1f1	1964-2004	1985-2025	2002-2021	2020-2039	2033-2052	2057-2076	2077-2096
67	CESM2-LE	ssp370	r67i1p1f1	1958-1998	1980-2020	1997-2016	2018-2037	2031-2050	2056-2075	2076-2095
68	CESM2-LE	ssp370	r68i1p1f1	1964-2004	1986-2026	2004-2023	2020-2039	2034-2053	2058-2077	2077-2096
69	CESM2-LE	ssp370	r69i1p1f1	1960-2000	1984-2024	2002-2021	2016-2035	2034-2053	2056-2075	2078-2097
70	CESM2-LE	ssp370	r70i1p1f1	1941-1981	1978-2018	1996-2015	2013-2032	2027-2046	2056-2075	2076-2095
71	CESM2-LE	ssp370	r71i1p1f1	1960-2000	1982-2022	1999-2018	2013-2032	2030-2049	2058-2077	2078-2097
72	CESM2-LE	ssp370	r72i1p1f1	1965-2005	1986-2026	2002-2021	2022-2041	2035-2054	2060-2079	2079-2098
73	CESM2-LE	ssp370	r73i1p1f1	1965-2005	1986-2026	2004-2023	2022-2041	2036-2055	2061-2080	2081-2100
74	CESM2-LE	ssp370	r74i1p1f1	1966-2006	1986-2026	2000-2019	2018-2037	2032-2051	2057-2076	2077-2096
75	CESM2-LE	ssp370	r75i1p1f1	1963-2003	1985-2025	2001-2020	2022-2041	2034-2053	2057-2076	2078-2097
76	CESM2-LE	ssp370	r76i1p1f1	1968-2008	1986-2026	2002-2021	2022-2041	2035-2054	2059-2078	2079-2098
77	CESM2-LE	ssp370	r77i1p1f1	1967-2007	1985-2025	2002-2021	2020-2039	2032-2051	2057-2076	2077-2096
78	CESM2-LE	ssp370	r78i1p1f1	1959-1999	1984-2024	2000-2019	2022-2041	2034-2053	2058-2077	2078-2097
79	CESM2-LE	ssp370	r79i1p1f1	1962-2002	1984-2024	1999-2018	2021-2040	2037-2056	2060-2079	2078-2097
80	CESM2-LE	ssp370	r80i1p1f1	1965-2005	1984-2024	1999-2018	2020-2039	2033-2052	2058-2077	2078-2097
81	CESM2-LE	ssp370	r81i1p1f1	1959-1999	1980-2020	1997-2016	2012-2031	2031-2050	2055-2074	2075-2094
82	CESM2-LE	ssp370	r82i1p1f1	1963-2003	1985-2025	2002-2021	2022-2041	2036-2055	2060-2079	2080-2099
83	CESM2-LE	ssp370	r83i1p1f1	1957-1997	1982-2022	1999-2018	2017-2036	2031-2050	2057-2076	2077-2096
84	CESM2-LE	ssp370	r84i1p1f1	1966-2006	1987-2027	2004-2023	2021-2040	2033-2052	2060-2079	2077-2096
85	CESM2-LE	ssp370	r85i1p1f1	1968-2008	1987-2027	2005-2024	2024-2043	2036-2055	2061-2080	2080-2099
86	CESM2-LE	ssp370	r86i1p1f1	1967-2007	1987-2027	2004-2023	2022-2041	2033-2052	2057-2076	2076-2095
87	CESM2-LE	ssp370	r87i1p1f1	1963-2003	1984-2024	2001-2020	2021-2040	2033-2052	2054-2073	2077-2096
88	CESM2-LE	ssp370	r88i1p1f1	1966-2006	1984-2024	2000-2019	2020-2039	2033-2052	2060-2079	2080-2099
89	CESM2-LE	ssp370	r89i1p1f1	1964-2004	1986-2026	2005-2024	2020-2039	2032-2051	2058-2077	2077-2096
90	CESM2-LE	ssp370	r90i1p1f1	1963-2003	1984-2024	2000-2019	2017-2036	2030-2049	2054-2073	2077-2096
91	CESM2-LE	ssp370	r91i1p1f1	1969-2009	1986-2026	2000-2019	2019-2038	2034-2053	2060-2079	2079-2098
92	CESM2-LE	ssp370	r92i1p1f1	1970-2010	1988-2028	2002-2021	2022-2041	2035-2054	2058-2077	2077-2096
93	CESM2-LE	ssp370	r93i1p1f1	1967-2007	1987-2027	2003-2022	2020-2039	2032-2051	2057-2076	2077-2096
94	CESM2-LE	ssp370	r94i1p1f1	1968-2008	1988-2028	2004-2023	2021-2040	2034-2053	2059-2078	2081-2100
95	CESM2-LE	ssp370	r95i1p1f1	1964-2004	1986-2026	2003-2022	2021-2040	2035-2054	2060-2079	2079-2098
96	CESM2-LE	ssp370	r96i1p1f1	1969-2009	1988-2028	2004-2023	2020-2039	2034-2053	2059-2078	2079-2098
97	CESM2-LE	ssp370	r97i1p1f1	1963-2003	1984-2024	2001-2020	2018-2037	2032-2051	2057-2076	2078-2097
98	CESM2-LE	ssp370	r98i1p1f1	1964-2004	1985-2025	2000-2019	2021-2040	2033-2052	2056-2075	2076-2095
99	CESM2-LE	ssp370	r99i1p1f1	1964-2004	1986-2026	2001-2020	2022-2041	2033-2052	2057-2076	2077-2096
100	CESM2-LE	ssp370	r100i1p1f1	1967-2007	1987-2027	2002-2021	2018-2037	2032-2051	2058-2077	2077-2096

Table S3. CESM2 large ensemble members used for SSP3-7.0

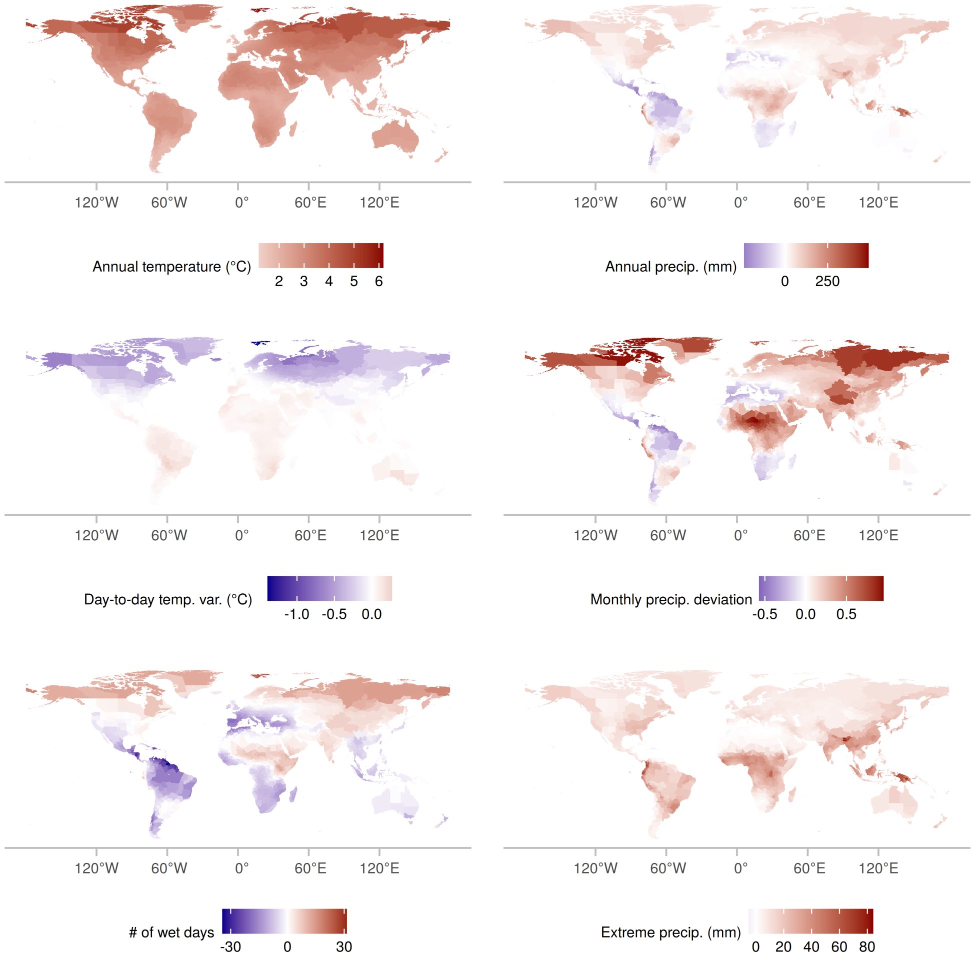

Figure S5. Average ADM1-level shift in climate indicators across CMIP6 models at +3°C of global warming vis-a-vis the respective +0.84°C global warming level window. For the two large ensembles, only the r1i1p1f1 model run is considered. ADM1-level shapefiles come from the GADM database (version 3.6)[38] and are sourced from ref.[39].

	Model	Scenario	Ensemble	Year	GWL	GID_1	Maximum monthly precip. deviation
1	MPI-ESM1-2-LR	ssp370	r24i1p1f1	2089	3°C	OMN.6_1	24.7
2	EC-Earth3-Veg-LR	ssp370	r1i1p1f1	2047	2°C	LBY.16_1	11.6
3	CNRM-ESM2-1	ssp370	r1i1p1f2	2035	1.5°C	SDN.1_1	11.3
4	INM-CM4-8	ssp370	r1i1p1f1	2057	2°C	SDN.10_1	11.3
5	GFDL-ESM4	ssp370	r1i1p1f1	2093	3°C	UMI.3_1	11.2
6	EC-Earth3	ssp126	r1i1p1f1	2040	2°C	ECU.9_1	10.7
7	CESM2	ssp370	r11i1p1f1	1984	0.38°C	SHN.1_1	9.6
8	NorESM2-LM	ssp126	r1i1p1f1	1995	0.38°C	TCD.6_1	9.0
9	BCC-CSM2-MR	ssp370	r1i1p1f1	2037	1.5°C	UMI.3_1	8.7
10	EC-Earth3-Veg	ssp370	r1i1p1f1	2053	3°C	MLI.4_1	8.5
11	UKESM1-0-LL	ssp119	r1i1p1f2	2031	1.5°C	OMN.6_1	8.3
12	CMCC-CM2-SR5	ssp370	r1i1p1f1	2033	1.5°C	UMI.3_1	8.3
13	EC-Earth3-AerChem	ssp370	r1i1p1f1	2073	3°C	ECU.9_1	7.9
14	CNRM-CM6-1	ssp370	r1i1p1f2	2073	3°C	SDN.1_1	7.8
15	CESM2-WACCM	ssp370	r1i1p1f1	2055	3°C	UMI.3_1	6.5
16	NorESM2-MM	ssp370	r1i1p1f1	2089	3°C	ECU.9_1	6.4
17	IPSL-CM6A-LR	ssp370	r1i1p1f1	2047	3°C	TCD.3_1	6.4
18	ACCESS-ESM1-5	ssp370	r1i1p1f1	2077	3°C	UMI.3_1	6.1
19	KACE-1-0-G	ssp370	r1i1p1f1	2046	3°C	PAK.8_1	6.1
20	ACCESS-CM2	ssp370	r1i1p1f1	2061	3°C	PNG.18_1	6.0
21	FGOALS-g3	ssp370	r1i1p1f1	2090	3°C	UMI.3_1	5.8
22	MRI-ESM2-0	ssp370	r1i1p1f1	2074	3°C	UMI.3_1	5.8
23	HadGEM3-GC31-LL	ssp126	r1i1p1f3	2039	2°C	UMI.3_1	5.7
24	CanESM5	ssp370	r1i1p1f1	2050	3°C	UMI.3_1	5.6
25	NESM3	ssp126	r1i1p1f1	2040	2°C	ESH.2_1	5.5
26	MPI-ESM1-2-HR	ssp126	r1i1p1f1	2043	1.5°C	PAK.8_1	5.4
27	HadGEM3-GC31-MM	ssp126	r1i1p1f3	1981	0.38°C	SHN.1_1	4.9
28	MIROC-ES2L	ssp370	r1i1p1f2	2087	3°C	TCD.3_1	4.7
29	MIROC6	ssp370	r1i1p1f1	2066	2°C	ERI.3_1	4.4
30	INM-CM5-0	ssp370	r1i1p1f1	2094	3°C	NER.3_1	4.2
31	TaiESM1	ssp370	r1i1p1f1	2067	3°C	OMN.8_1	3.9
32	KIOST-ESM	ssp126	r1i1p1f1	2028	1.5°C	TCD.6_1	3.3
33	CNRM-CM6-1-HR	ssp126	r1i1p1f2	2018	1.5°C	BTN.17_1	2.5

Table S4. Maximum values for monthly precipitation deviation in raw CMIP6 outputs for global warming up to +3°C. The upper bound for the bias-corrected monthly precipitation deviation is based on the maximum value for EC-Earth3-Veg-LR (i.e., 11.6) because maximum values produced by the MPI-ESM1-2-LR large ensemble before bias correction represent clear outliers across all CMIP6 models under consideration, including the CESM2 large ensemble.

Appendix B - Additional results and information on GDP impact projections

Omitted non-sovereign ADM0-level territories

All country-level results displayed in this paper include only sovereign ADM0-level countries and, therefore, do not include the following ADM0-level territories that are featured in the GADM database (v3.6): Akrotiri and Dhekelia, Åland, American Samoa, Aruba, Bermuda, Bonaire, Sint Eustatius and Saba, British Virgin Islands, Cayman Islands, Faroe Islands, French Guiana, French Polynesia, French Southern Territories, Greenland, Guadeloupe, Guam, Guernsey, Hong Kong, Isle of Man, Jersey, Macao, Martinique, Mayotte, Montserrat, New Caledonia, Northern Cyprus, Northern Mariana Islands, Palestina, Puerto Rico, Reunion, Saint Helena, Saint Pierre and Miquelon, Svalbard and Jan Mayen, Tokelau, Turks and Caicos Islands, United States Minor Outlying Islands, U.S. Virgin Islands, Wallis and Futuna, Western Sahara.

	Impact channel	+1°C	+1.5°C	+2°C	+3°C	+4°C
1	Annual temperature	-0.82% (-2.27 to 0.46%)	-3.32% (-5.46 to -1.41%)	-5.63% (-8.65 to -2.83%)	-10.04% (-14.83 to -5.33%)	-14.23% (-20.64 to -7.75%)
2	Annual precip.	0.02% (-0.17 to 0.21%)	0.08% (-0.12 to 0.31%)	0.13% (-0.09 to 0.4%)	0.21% (-0.05 to 0.53%)	0.34% (0.01 to 0.76%)
3	Day-to-day temp var.	0.03% (-0.43 to 0.49%)	0.08% (-0.37 to 0.53%)	0.09% (-0.33 to 0.55%)	0.11% (-0.35 to 0.58%)	0.09% (-0.47 to 0.66%)
4	Monthly precip. deviation	0.02% (-0.15 to 0.19%)	-0.01% (-0.23 to 0.2%)	-0.05% (-0.31 to 0.2%)	-0.2% (-0.58 to 0.14%)	-0.55% (-1.3 to 0.04%)
5	# of wet days	0.02% (-0.34 to 0.39%)	0.06% (-0.33 to 0.47%)	0.1% (-0.33 to 0.56%)	0.23% (-0.27 to 0.72%)	0.27% (-0.28 to 0.87%)
6	Extreme precip.	-0.01% (-0.11 to 0.08%)	-0.07% (-0.18 to 0.02%)	-0.12% (-0.25 to -0.01%)	-0.24% (-0.43 to -0.08%)	-0.41% (-0.7 to -0.16%)
7	All indicators	-0.76% (-2.2 to 0.51%)	-3.2% (-5.44 to -1.21%)	-5.5% (-8.61 to -2.62%)	-9.96% (-14.89 to -5.12%)	-14.46% (-21.11 to -7.77%)
8	Total impacts (status quo)	-0.66% (-1.83 to 0.34%)	-2.67% (-4.57 to -0.99%)	-4.54% (-7.29 to -2%)	-8.17% (-12.62 to -3.83%)	-11.64% (-17.68 to -5.6%)
9	Difference to status quo	-0.1%	-0.54%	-0.96%	-1.79%	-2.81%

Table S5. Global mean GDP impacts by warming level and climate indicator (upper and lower decile in parentheses)

	Country	Year	Annual GDP growth
1	Syria	2012	-26.3%
2	Greece	2011	-10.15%
3	World	2009	-1.34%
4	World	2020	-3.11%

Table S6. Examples for historical year-to-year contractions in real GDP (in 2015 USD) from the World Bank's World Development Indicators database used in Figure 2

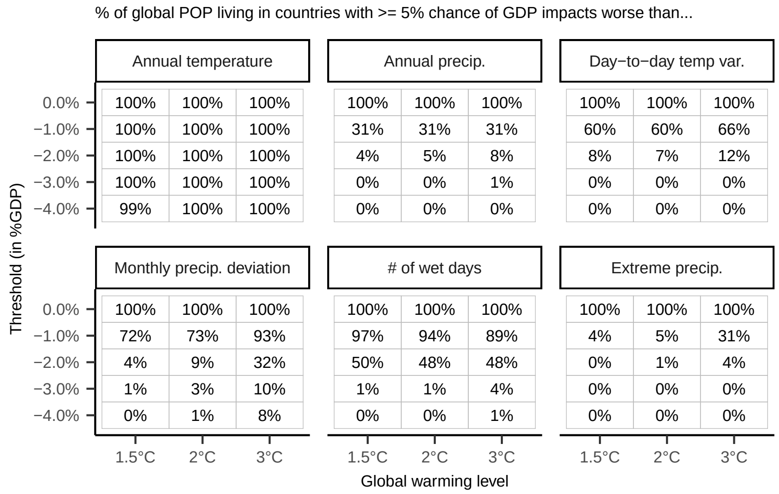

Figure S6. Exposure of global population to tail GDP impacts (95th percentile). Share of the current global population that lives in countries whose projected GDP impacts for the respective climate indicator (panel) and warming level (x-axis) exceed the given threshold (y-axis) for at least 5% of the GDP impact distribution.

Appendix C - Additional results and information on the dose-response functions

Regressions underlying the dose-response functions

Dose-response functions used for our damage projections are derived using the sample and main specification by Kotz et al. (2022), which reads as follows:

$$
\begin{aligned}
g_{i,t} =\ & \underbrace{\beta_1^T(T_{i,t}-T_{i,t-1})+\beta_2^T(T_{i,t-1}-T_{i,t-2})+\beta_3^T(T_{i,t}-T_{i,t-1})T_{i,t}+\beta_4^T(T_{i,t-1}-T_{i,t-2})T_{i,t-1}}_{\text{Annual mean temperature terms}}+\\
& \underbrace{\beta_1^{RA}RA_{i,t}+\beta_2^{RA}RA_{i,t}^2}_{\text{Annual precip. terms}}+\\
& \underbrace{\beta^{\tilde{T}}\tilde{T}+\beta_1^{RD}RD_{x,t}+\beta_2^{RD}RD_{x,t}^2+\beta_1^{RM}RM_{i,t}+\beta_2^{RM}RM_{i,t}^2+\beta_1^{\hat{RD}}\hat{RD}_{x,t}+\beta_2^{\hat{RD}}\hat{RD}_{x,t}T_{i,t}}_{\text{Variability and extremes terms}}+\\
& \underbrace{\alpha_i+\delta_t}_{\text{Fixed effects}}+\varepsilon_{i,t}
\end{aligned}
\tag{S5}
$$

Here, $g_{i,t}$ denotes the first difference of the log-transformed gross regional product per capita of subnational region i in year t (for more information on the economic growth data, see Wenz et al. (2023)[40]). T, $\tilde{T}$, RA, RD, RM, and $\hat{RD}$ denote annual mean temperature, day-to-day temperature variability, annual precipitation, the number of wet days, the monthly precipitation deviation, and extreme precipitation, respectively. α_i, δ_t, and $\varepsilon_{i,t}$ denote region and year fixed effects and the error term, respectively. Regression coefficients and standard errors using country-level clustering are displayed in Table S7 (column 5) and shown in Figure S7.

To simulate a "status quo" approach where the temperature dose-response function is estimated solely by controlling for annual precipitation and only annual temperature impacts are projected, we remove all "Variability and extremes terms" from the specification in Equation (S5) and re-estimate the model using the same sample (see column 1 in Table S7).

Step-by-step mathematical expressions for projecting the dose-response functions

We use the following dose-response functions for the respective climate indicators:

$$h_{i,t}^{RA} = \beta_1^{RA}RA_{i,t}+\beta_2^{RA}RA_{i,t}^2 \qquad \text{(annual precipitation)} \tag{S6}$$

$$h_{i,t}^{\tilde{T}} = \beta^{\tilde{T}}\tilde{T} \qquad \text{(day-to-day temperature variability)} \tag{S7}$$

$$h_{i,t}^{RD} = \beta_1^{RD}RD_{x,t}+\beta_2^{RD}RD_{x,t}^2 \qquad \text{(number of wet days)} \tag{S8}$$

$$h_{i,t}^{RM} = \beta_1^{RM}RM_{i,t}+\beta_2^{RM}RM_{i,t}^2 \qquad \text{(monthly precipitation deviation)} \tag{S9}$$

For the extreme precipitation dose-response function, we interact extreme precipitation with the average annual temperature during the 41-year baseline period K, during which global warming equaled +0.84°C, instead of the current year's annual mean temperature:

$$h_{i,t}^{\hat{RD}} = \beta_1^{\hat{RD}}\hat{RD}_{x,t}+\beta_2^{\hat{RD}}\hat{RD}_{x,t}\frac{1}{41}\sum_{k\in K}T_{i,k} \qquad \text{(extreme precipitation)} \tag{S10}$$

As we explain in the **Methods** section, doing so ensures that for all indicators alike, the implicit assumption is that adaptive capacity remains constant at historically observed levels. Since $\beta_2^{\hat{RD}}$ is positive (see Table S7), if we were to use current annual mean temperatures in the interaction term, temperature increases due to global warming would reduce the projected impacts of extreme precipitation increases. As noted in the **Methods** section, such positive feedback of global temperature rise on extreme precipitation has no empirical foundation, particularly given that Kotz et al. (2022)[2] report similar explanatory power for a

model that uses a country's latitude for the interaction term instead of annual mean temperature (see R2 and Adjusted R2 in their Table S4).

Notably, the main specification by Kotz et al. (2022)[2] combines temperature variability and precipitation terms in absolute levels with annual temperature terms in first differences (interacted with absolute temperature levels). The functional form for the annual temperature terms is based on the empirical work on the GDP-temperature relationship by Kalkuhl and Wenz (2020)[26]. The authors turn the regression coefficients into GDP damage projections by calculating cumulative impacts across the first differences over time (see their Supplementary Materials section S4), which we follow here:

$$h_{i,t}^{T}(T_{i,t},...) = \sum_{j=k_0}^{t}\left(\underbrace{\beta_1^T(T_{i,j}-T_{i,j-1})}_{\text{First diff.}} + \underbrace{\beta_2^T(T_{i,j-1}-T_{i,j-2})}_{\text{First diff. (lag)}} + \underbrace{\beta_3^T(T_{i,j}-T_{i,j-1})T_{i,j} + \beta_4^T(T_{i,j-1}-T_{i,j-2})T_{i,j-1}}_{\text{Interactions with temperature levels}}\right) \quad \text{(S11)}$$

Here, k_0 denotes the first year considered, which in our case is the first year of the 41-year baseline period.

While this expression is mathematically different from the dose-response functions above, they are logically consistent: If annual precipitation RA increases sharply in one year and remains constant at this increased level after that, so will the result of the dose-response function h^{RA}, such that these higher precipitation levels will continue to affect the economy, positively or negatively. Now consider a similar example where annual mean temperature T in a warm region rises by an additional +1°C in one year and remains constant after that. If Equation (S11) did not feature cumulative impacts (i.e., if the sum were to start and end in year t), then the dose-response function would indicate a GDP impact only in the first and second year and no GDP impacts in all subsequent years (for which the first difference and its lag would equal zero), although temperature levels are permanently higher than before. By contrast, calculating cumulative impacts ensures that h^T will increase over the first two years and then *remain constant at the increased level*, consistent with the behavior of a dose-response function that features climate indicators in levels instead of first differences. At the same time, this approach introduces adverse impacts of inter-annual temperature variability. If a +1°C temperature increase from an initial temperature level T_0 is immediately followed by a -1°C decrease in the next year (back to the initial T_0), the β_3^T and β_4^T terms interact the +1°C increase with T_0+1 but the subsequent decrease only with T_0. As $\beta_3^T, \beta_4^T < 0$, this means that the cumulative sum over the two years produces negative impacts that persist. To our knowledge, this behavior of the methodology by Kalkuhl & Wenz (2020)[26] is not documented in the literature. We also note that the sign of these variability-related impacts can flip if temperature first differences are interacted with lagged instead of current temperatures. Removing the variability-related part of the temperature damages would reduce impacts in high-latitude countries particularly but have no major implication for the main conclusions in this study.

To calculate GDP impacts from shifts in climate indicators relative to the 41-year baseline period K, we calculate the change in the dose-response function value relative to the average value during the baseline period for any climate indicator C as follows:

$$\delta_{i,t,log}^{C} = h_{i,t}^{C} - \frac{1}{41}\sum_{k\in K} h_{i,k}^{C} \quad \text{(S12)}$$

Since the dependent variable of the regression model is the first difference of the log-transformed gross regional product per capita[40], all dose-response functions h^C return values in logarithmic changes and hence $\delta_{i,t,log}^{C}$ denotes the GDP impact of climatic shifts in climate indicator C in region i and year t relative to the baseline period, expressed as a logarithmic change. Notably, the terms in the model specification in Equation (S5) are additive, such that we can add GDP impacts in logarithmic changes from different climate indicators to calculate their combined impact:

$$\delta_{i,t,log}^{Total} = \sum_{C}\left(h_{i,t}^{C} - \frac{1}{41}\sum_{k\in K} h_{i,k}^{C}\right) \quad \text{(S13)}$$

Similarly, we can calculate the combined impacts of the four variability and extremes indicators alone by excluding the dose-response function for annual temperature and precipitation:

$$\delta_{i,t,log}^{VarExtr} = \sum_{C\in\{\tilde{T},RD,\hat{RD},RM\}}\left(h_{i,t}^{C} - \frac{1}{41}\sum_{k\in K} h_{i,k}^{C}\right) \quad \text{(S14)}$$

Lastly, we can convert GDP impacts (by individual climate indicators or by a combination of indicators, such as $\delta^{Total}_{i,t,log}$ or $\delta^{VarExtr}_{i,t,log}$) from the logarithmic changes of the underlying regression model to % of GDP as follows:

$$\delta^{C}_{i,t} = exp(\delta^{C}_{i,t,log}) - 1 \tag{S15}$$

To confirm this, let y^{log} denote the logarithmic change between GDP in one year GDP_1 and in the following year GDP_2, so $g^{log} = ln(GDP_2) - ln(GDP_1) = ln\left(\frac{GDP_2}{GDP_1}\right)$. Consider that GDP changed by y percent between the two years, so $GDP_2 = (1+y) \times GDP_1$. Then

$$exp(y^{log}) = \frac{GDP_2}{GDP_1} = 1 + y$$

and subtracting one yields

$$exp(y^{log}) - 1 = y$$

Notably, this result is independent of GDP_1 and GDP_2, so we can calculate relative GDP losses of subnational regions for each year without using GDP levels. For small changes, $\delta^{C}_{i,t}$ and $\delta^{C}_{i,t,log}$ are approximately identical, but they increasingly diverge for larger GDP impacts. For instance, a logarithmic change of $\delta^{C}_{i,t,log} = -0.1$ is equivalent to a loss of approximately $\delta^{C}_{i,t} = -9.52\%$ of GDP. Unlike impacts expressed in logarithmic changes ($\delta^{C}_{i,t,log}$), impacts expressed in % of GDP ($\delta^{C}_{i,t}$) are no longer additive across climate indicators, which is why we add impacts of different indicators and average impacts across baseline years *before* transforming logarithmic changes to % of GDP.

All GDP impact projections are carried out at the subnational region level, consistent with the resolution of the underlying regression model[2]. For each year t, we then aggregate relative GDP impacts in % of GDP to the country level by averaging across all regions located in a given country J, using the region's initial share in the respective country's GDP w_i as a weight (for more information on the GDP weights deployed, see **Methods**):

$$\sum_{i \in J} w_i \delta^{C}_{i,t} \tag{S16}$$

where $\sum_{i \in J} w_i = 1$. To confirm this, note that the regional GDP net of the climate change impacts of climate indicator C is defined as

$$GDP^{CC}_{i} = GDP_i \times (1 + \delta^{C}_{i,t})$$

where GDP_i is regional GDP in the absence of climate change impacts. Since regional GDP sums up to country-level GDP, the relative GDP impact on country J (in % of GDP) is

$$\frac{\sum_{i \in J} GDP^{CC}_{i}}{\sum_{i \in J} GDP_i} - 1$$

Inserting the definition of GDP^{CC}_{i} and simplifying then yields

$$\frac{\sum_{i \in J} \delta^{C}_{i,t} \times GDP_i}{\sum_{i \in J} GDP_i}$$

which is the weighted average of region-level impacts in % of GDP $\delta^{C}_{i,t}$ weighted by their share in the country-level GDP $w_i = \frac{GDP_i}{\sum_{i \in J} GDP_i}$.

Similarly, the derived country-level GDP impacts are further aggregated to the global level by weighting each country with its share in the global economy per the respective SSP. Therefore, differences between SSPs affect our results regarding relative GDP losses only insofar as they change the weight of countries in the GDP loss of the global economy.

For the regression coefficients (betas) in Equations S6–S11, we draw $N = 1{,}000$ Monte Carlo draws from the multivariate Gaussian distribution based on the point estimates and the variance-covariance matrix of the regression model in Table S7, column 5 (using standard errors clustered at the country level). Performing all steps outlined above separately for each Monte Carlo draw then provides us with 1,000 different GDP impact projections $\delta^{C}_{i,t}$ for each model-scenario-realization-year and territory i (which can be either a subnational region, a country, or the global economy). Notably, one multivariate Monte Carlo

draw features values for *all* betas in Equations S6–S11, so our Monte Carlo draws account for the correlations between the regression coefficients of different climate indicators. For some complementary analyses in the Supplementary Information, however, we only use the point estimates in Table S7, column 5, instead of all 1,000 Monte Carlo draws to reduce the computational requirements.

Damage persistence (level effects versus growth effects)

A key assumption when projecting GDP impacts of climate change is whether the contemporaneous impacts of climatic indicators on growth are temporary such that the economy rebounds in the following year (i.e., a so-called "level effect") or whether the impact persists over time, changing the long-run growth trajectory (i.e., a so-called "growth effect" or "persistent damage"). Importantly, this assumption is not pre-determined by whether the dependent variable in the underlying regression model is expressed in levels or, as is the case in Equation (S5), as a growth rate. For instance, both Yumashev et al. (2019)[41] and Burke et al. (2015)[25] use the same dose-response function estimated by Burke et al. (2015)[25], which is based on a regression model with output per capita growth as the dependent variable. However, while Burke et al. (2015)[25] assume damage persistence when projecting future damages, Yumashev et al. (2019)[41] use the more conservative "level effects" approach without persistence.

To illustrate the difference between these two approaches in the context of our study, note that we calculate the total GDP impact for a region i in year t as indicated in Equation (8) in our **Methods** section:

$$\delta_{i,t} = exp\left(\sum_C h^C(C_{i,t}) - \frac{1}{41}\sum_{k\in K}\sum_C h^C(C_{i,k})\right) - 1$$

After aggregating regional impacts to the country level via GDP weighting, we could then calculate the absolute GDP *level* of country j in year t including the effects of climate change similar to Yumashev et al. (2019)[41] as follows:

$$GDP^{CC}_{j,t} = GDP^{SSP}_{j,t} \times (1+\delta_{j,t}) \tag{S17}$$

where $GDP^{SSP}_{j,t}$ denotes absolute GDP in the absence of climate change impacts per the respective SSP. The value of $\delta_{j,t}$ in year t does not affect GDP in year $t+1$, so there is no damage persistence or path dependency implied by this implementation of $\delta_{j,t}$.

By contrast, an implementation assuming full persistence, such as Burke et al. (2015)[25], would calculate the GDP of country j in year t in the presence of climate change as follows:

$$GDP^{CC}_{j,t} = GDP_{j,0} \times \prod_{s=1}^{t}\left(1+g^{SSP}_{j,s}+\delta_{j,t}\right) \tag{S18}$$

Here, $GDP_{j,0}$ is the initial GDP level, and $g^{SSP}_{j,s}$ is the year-to-year growth rate in the absence of climate change implied by the respective SSP.

As a result, a shock to output in year t would persistently affect the growth trajectory in all years after t, even if the climate were to return to baseline levels at some point. The relative GDP change due to climate change can then be calculated as

$$\frac{GDP^{CC}_{j,t}}{GDP^{SSP}_{j,t}} - 1 \tag{S19}$$

which under an implementation without damage persistence would equal $\delta_{j,t}$ but in an implementation with damage persistence can reach considerably higher magnitudes for both positive and negative impacts[25, 42].

Therefore, the same estimate for $\delta_{j,t}$ can, in principle, be implemented with different assumptions regarding no persistence[41], full persistence[25], or partial persistence[42, 43]. To inform such assumptions, extant studies have used different statistical procedures. Most commonly, this is done by including lags of climate indicators in the regression model and testing if lags have a significant impact on economic growth and if the sum of impacts across all lags is zero (indicating no persistence) or significantly different from zero (indicating partial or full persistence)[44, 25, 26, 28, 2]. Other approaches applied involve long differences[45, 26] and lower-frequency variability[46]. While the impacts of persistence assumptions on projected damages are substantial[47, 42, 48], the empirical literature has not reached a clear consensus yet[44, 25, 49, 50, 26, 46, 28, 2].

For the dose-response functions deployed here, the study by Kalkuhl and Wenz (2020)[26], from which the annual temperature terms in Equation (S5) are derived, report no evidence for persistent growth effects. Consistently, they use an approach for

projecting future damages that, results-wise, is more closely related to the implementation without damage persistence given above (for more details, see below). Kotz et al. (2021)[28], who study day-to-day temperature variability, find some evidence for damage persistence for at least some years (see their Table S10). Regarding the precipitation-based terms in Equation (S5), Kotz et al. (2022)[2] find "no evidence for rebound effects in the short term, instead identifying some persistence in the effect of the annual total and number of wet days" (see their Table S16). Given the lack of a clear empirical consensus and established methods to consistently implement heterogeneous damage persistence for our multiple climate indicators, our paper applies a conservative projection without persistence, similar to Yumashev et al. (2019)[41]. Notably, this assumption implies that the relative GDP change due to climate change in Equation (S19) equals $\delta_{j,t}$, so that we do not actually need to calculate GDP levels via Equation (S17) to derive relative GDP losses, our key outcome of interest. However, we strongly recommend future work exploring the impacts of empirically grounded, indicator-specific, and consistently implemented damage persistence assumptions, noting that future studies supporting damage persistence might well render our implementation too conservative.

As shown in Equation (S11), our projection approach for annual mean temperature impacts following Kalkuhl and Wenz (2020)[26] adds up impacts from temperature first-differences of past years. Results-wise, however, this approach is more closely related to the "level effects" implementation by Yumashev et al. (2019)[41] than the "growth effects" implementation by Burke et al. (2015)[25] assuming full persistence[26]. A simple first intuition for this is that using a dose-response function expressed in absolute levels of an indicator (e.g., annual mean temperature) in Equation (S12) assumes that annual GDP impacts in year t are driven by the difference in temperature levels between the year t and the baseline period. Notably, this difference equals the *accumulation* of year-to-year temperature changes since the baseline period. For instance, Burke et al. (2015)[25] estimate a quadratic dose-response function of GDP per capita depending on annual temperature levels $h(T_t)$ and then calculate annual impacts $\delta_{t,log}$ (as logarithmic changes) based on a baseline period's average temperature level T_{base} as follows:

$$\delta_{t,log} = h(T_t) - h(T_{base}) \tag{S20}$$

Note that $h(T_t) - h(T_{base})$ can be rewritten as

$$[h(T_t) - h(T_{t-1})] + [h(T_{t-1}) - h(T_{t-2})] + ... + [h(T_1) - h(T_{base})]$$

which is the accumulation of year-to-year changes in the dose-response function since the baseline period. If $h(\cdot)$ were linear, this would reduce to

$$h(\Delta T_t) + h(\Delta T_{t-1}) + ...$$

which is the cumulative impact of temperature first differences. If $h(\cdot)$ is non-linear (the function estimated by Burke et al., 2015[25] is quadratic and concave), then for small temperature increments ΔT, the year-to-year changes in the dose-response function can still be linearly approximated as follows:

$$[h(T_t) - h(T_{t-1})] + [h(T_{t-1}) - h(T_{t-2})] + ... + [h(T_1) - h(T_{base})] \approx b_t \Delta T_t + b_{t-1} \Delta T_{t-1} + ... + b_1 \Delta T_1 \tag{S21}$$

where b_t denotes a position-specific slope parameter to approximate changes in $h(\cdot)$ between T_t and T_{t-1}. Note that such a linear approximation is similar to the dose-response function given in Equation (S5), which interacts the temperature first difference with absolute temperature levels, akin to the position-specific slopes given above. Therefore, one can think of the annual temperature dose-response function in Equation (S5) (which depends on temperature first differences) as a piece-wise linear approximation of a quadratic dose-response function that depends on absolute temperature levels. This provides some further intuition why calculating cumulative impacts over past temperature first differences (i.e., adding the pieces of the approximation) is *not* equivalent to the "growth effect" implementation by Burke et al. (2015)[25] assuming full persistence, as Kalkuhl and Wenz (2020)[26] show in their Supplementary Information (section S4). Indeed, Kalkuhl and Wenz (2020)[26] estimate a dose-response function with higher marginal impacts of temperature increases compared to Burke et al. (2015)[25] but project considerably *lower* global GDP losses. However, as explained above, interacting temperature first differences with time-varying temperature levels can introduce some persistent impacts driven by inter-annual temperature variability, which are implicit in the methodology by Kalkuhl & Wenz (2020)[26] employed here.

Marginal effects of annual mean temperature on income

We calculate the marginal effect of a +1°C increase in annual mean temperature on income growth (in logarithmic changes) following Kalkuhl and Wenz (2020)[26] as follows:

$$\beta_1^T + \beta_2^T + T_0(\beta_3^T + \beta_4^T) \tag{S22}$$

where the notation for the regression coefficients is taken from Equation (9) in the **Methods** section and T_0 denotes a given territory's initial temperature prior to the +1°C increase. The marginal effects in Figure 4b are based on the regression models in Table S7, columns 1, 2, and 5. To convert marginal effects from logarithmic changes to % of GDP, we exponentiate and subtract one.

Inflation adjustment in the dependent variable

Recently, a new study reviewing estimates for climate change damages[51] has criticized Kalkuhl and Wenz (2020)[26] due to the lack of inflation adjustment in the dependent variable (nominal income per capita growth). Notably, Kalkuhl and Wenz (2020)[26] have used the same dependent variable as Kotz et al. (2022)[2], our source of dose-response functions. In contrast, Callahan and Mankin (2022)[22] use the same sample but adjust for inflation using GDP deflator data for the US (as all subnational income values in the underlying sample are expressed in nominal USD). Therefore, we convert the sample's nominal per capita income values by Kotz et al. (2022)[2] into real terms based on World Bank data for the US GDP deflator, and then re-calculate the dependent variable by taking first differences of the log-transformed real per capita income. When re-estimating the model for the main specification by Kotz et al. (2022)[2], results are virtually identical (see Table S8). This is because all income per capita values are already expressed in USD such that all values from the same year are multiplied by the same inflation adjustment factor, which in log scale is absorbed by the year fixed effect. Therefore, we conclude that inflation adjustment following Callahan and Mankin (2022)[22] would not alter the findings presented in the main manuscript.

Table S7. Regression models used to estimate dose-response functions. Note that we display columns 3 and 4 for the sake of completeness, but we do not use these specifications for any of our results, and we use the results from column 2 only for Figure 4b in the main manuscript.

	Dependent variable:				
	Subnational income per capita growth				
	(1)	(2)	(3)	(4)	(5)
	"status quo"				Kotz et al., 2022
D.MeanTemp	6e-03	1.8e-03	1.6e-03	1.3e-03	9.6e-04
	(4.7e-03)	(4.8e-03)	(4.8e-03)	(4.8e-03)	(4.8e-03)
L.D.MeanTemp	-4.3e-04	-2.4e-03	-2.2e-03	-2.1e-03	-2.3e-03
	(5.8e-03)	(6e-03)	(6e-03)	(5.9e-03)	(5.9e-03)
D.MeanTemp:MeanTemp	-1e-03**	-1e-03*	-1.1e-03**	-1.1e-03**	-1.1e-03**
	(4.9e-04)	(5.2e-04)	(4.9e-04)	(5e-04)	(5.1e-04)
L.D.MeanTemp:L.MeanTemp	-6e-04	-5.5e-04	-6.7e-04	-6.6e-04	-6.5e-04
	(5.2e-04)	(5.3e-04)	(5.2e-04)	(5.2e-04)	(5.2e-04)
Annual precip.	1.5e-05	1e-05	6.3e-05**	4.3e-05*	5.8e-05**
	(2.3e-05)	(2.3e-05)	(2.7e-05)	(2.6e-05)	(2.7e-05)
Annual precip. sq.	-4e-10	1e-10	-3.9e-09**	-2.8e-09*	-3.8e-09**
	(1.9e-09)	(1.9e-09)	(1.8e-09)	(1.6e-09)	(1.7e-09)
Day-to-day temp. var.		-5.3e-02***	-5.8e-02***	-5.7e-02***	-5.8e-02***
		(0.017)	(0.017)	(0.016)	(0.016)
No. of wet days			-9.9e-04**	-1.3e-03**	-1.3e-03**
			(4.7e-04)	(5.2e-04)	(5.3e-04)
No. of wet days sq			7.3e-07	1.2e-06	1.1e-06
			(1.2e-06)	(1.2e-06)	(1.2e-06)
Monthly precip. deviation				0.014	0.017*
				(0.01)	(0.01)
Monthly precip. deviation sq.				-2.9e-02***	-2.8e-02***
				(9.8e-03)	(9.9e-03)
Extreme precip.					-3.7e-04***
					(8.7e-05)
Extreme precip.:MeanTemp					1.3e-05***
					(3.7e-06)
Observations	30121	30121	30121	30121	30121
R^2	2.9e-03	8.2e-03	0.011	0.013	0.014
Adjusted R^2	-5.3e-02	-4.7e-02	-4.5e-02	-4.3e-02	-4.1e-02
Note:	St. err. clustered at the country level. *p<0.1; **p<0.05; ***p<0.01				

Table S8. Kotz et al. (2022) main specification with and without inflation adjustment via US GDP deflator

	Dependent variable:	
	Growth in subnational income per capita	
	(1)	(2)
	Kotz et al., 2022	Inflation-adjusted
D.MeanTemp	9.6e-04	9.6e-04
	(4.8e-03)	(4.8e-03)
L.D.MeanTemp	-2.3e-03	-2.3e-03
	(5.9e-03)	(5.9e-03)
D.MeanTemp:MeanTemp	-1.1e-03**	-1.1e-03**
	(5.1e-04)	(5.1e-04)
L.D.MeanTemp:L.MeanTemp	-6.5e-04	-6.5e-04
	(5.2e-04)	(5.2e-04)
Annual precip.	5.8e-05**	5.8e-05**
	(2.7e-05)	(2.7e-05)
Annual precip. sq.	-3.8e-09**	-3.8e-09**
	(1.7e-09)	(1.7e-09)
Day-to-day temp. var.	-5.8e-02***	-5.8e-02***
	(0.016)	(0.016)
Monthly precip. deviation	0.017*	0.017*
	(0.01)	(0.01)
Monthly precip. deviation sq.	-2.8e-02***	-2.8e-02***
	(9.9e-03)	(9.9e-03)
No. of wet days	-1.3e-03**	-1.3e-03**
	(5.3e-04)	(5.3e-04)
No. of wet days sq.	1.1e-06	1.1e-06
	(1.2e-06)	(1.2e-06)
Extreme precip.	-3.7e-04***	-3.7e-04***
	(8.7e-05)	(8.7e-05)
Extreme precip.:MeanTemp	1.3e-05***	1.3e-05***
	(3.7e-06)	(3.7e-06)
Region fixed effects	Yes	Yes
Year fixed effects	Yes	Yes
Observations	30121	30121
R^2	0.014	0.014
Adjusted R^2	-4.1e-02	-4.1e-02
Note: St. err. clustered at the country level	*p<0.1; **p<0.05; ***p<0.01	

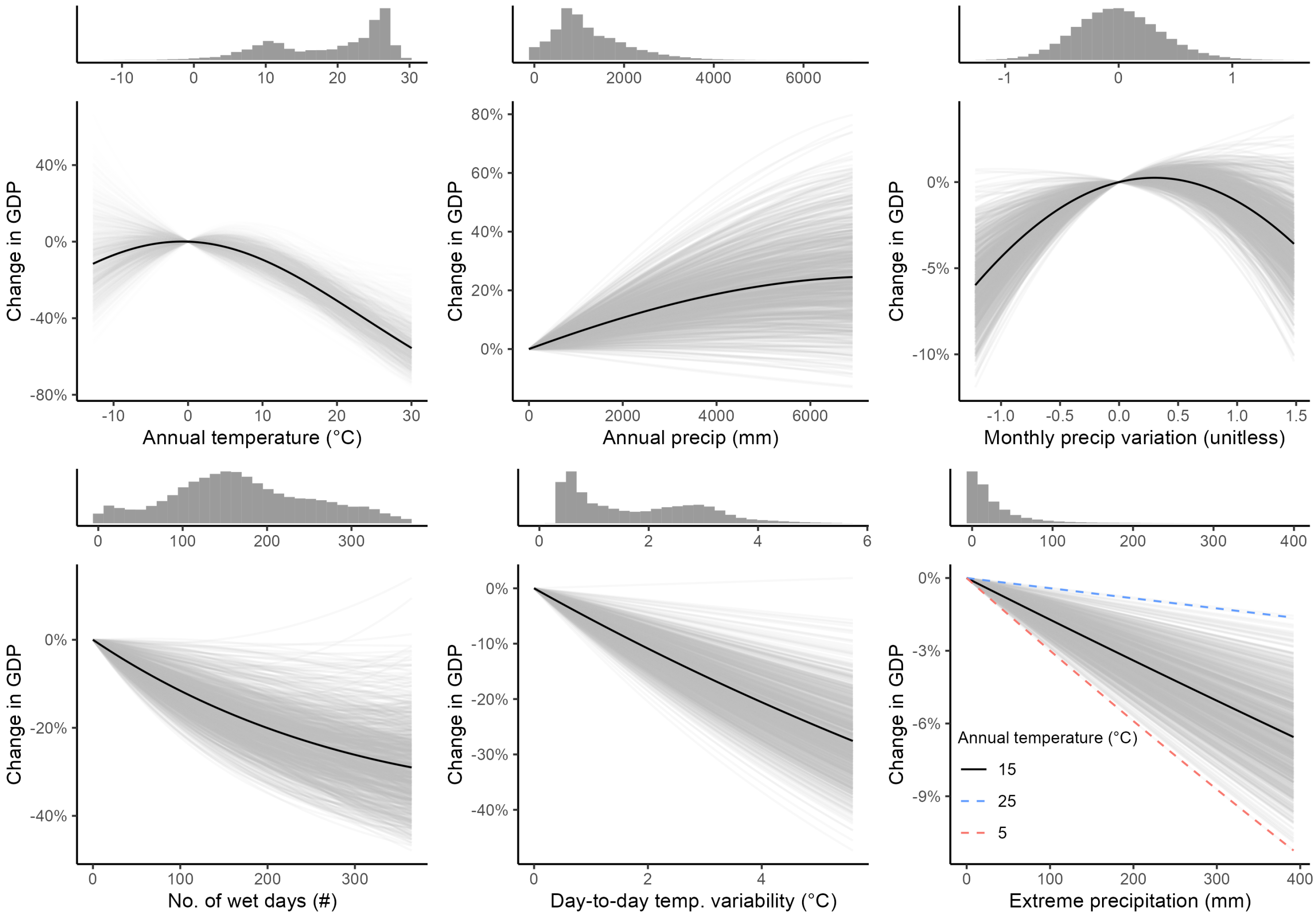

Figure S7. Dose-response functions for all six climate indicators used for damage projections, with ADM1-level distributions during the +0.84°C baseline period for all CMIP6 models. The black line indicates the dose-response function based on point estimates by Kotz et al. (2022)[2] (Table S7, column 5), while grey lines denote $N = 1,000$ draws from the multivariate Gaussian distribution based on standard errors clustered at the country level. Marginal histograms display the distribution of ADM1 regions for the respective climate indicator across all CMIP6 models during the +0.84°C baseline period. To facilitate readability, output values of the dose-response functions are transformed from the original changes in the first difference of log-transformed output to % of GDP by exponentiating and subtracting one (see **Methods**). As a result, functions that are linear when displaying logarithmic changes (day-to-day temperature variability and extreme precipitation) appear slightly non-linear. Since the dose-response function for extreme precipitation interacts with the annual mean temperature, we show parameter draws and the black line for an example value of 15°C and illustrate how the black line would shift for different temperature levels via the dashed lines. As the dose-response function for annual mean temperature given in Equation (S11) depends on temperature first differences instead of temperature levels, the function displayed here is derived by integrating the formula for the marginal effect of annual mean temperature given in Equation (S22) and serves illustrative purposes. Value ranges displayed for each climate indicator range from the 0.1th to the 99.9th percentile in the baseline period across all CMIP6 models, omitting extreme outliers for visual purposes. For the two large ensembles, only the r1i1p1f1 model run is displayed to prevent the large ensembles from dominating the distribution shown and to reduce computational requirements.

Appendix D - Additional results and information on variance decompositions

Mathematical expressions for variance components

Here, we complement the verbal description of our variance decomposition methodology (see **Methods** in the main manuscript) with mathematical expressions. Let $\delta^C_{t,m,r,s,b}$ denote the global GDP impacts (in % of GDP) due to climate indicator C in year t based on the realization r of CMIP6 model m under RCP-SSP scenario s using dose-response function parameter draw b. Note that δ^C can also denote the joint impacts of multiple climate indicators, such as the impact of all variability and extremes or the total impacts of all indicators (see Appendix C). Let GWL be the set of all model-realization-scenario-year combinations that imply a given global warming level. Then, the variance of GDP impacts conditional on a given warming level GWL can be expressed as

$$Var(\delta^C_{t,m,r,s,b}|GWL)$$

By using only the point estimates of dose-response function parameters $\bar{b}$ (i.e., the coefficients in Table S7, column 5), the variance of GDP impacts excluding dose-response function uncertainty can be expressed as

$$Var(\delta^C_{t,m,r,s,b}|GWL, b=\bar{b})$$

Using the law of total variance, we can decompose this expression as follows:

$$Var(\delta^C_{t,m,r,s,b}|GWL, b=\bar{b}) = Var_m\left(E[\delta^C_{t,m,r,s,b}|GWL, b=\bar{b}, m]\right) + E_m[Var(\delta^C_{t,m,r,s,b}|GWL, b=\bar{b}, m)] \quad \text{(S23)}$$

Following Schwarzwald and Lenssen (2022)[23], we use the first term (i.e., the variance *between* different climate models) to quantify *climate model uncertainty* and the second term (i.e., the variance *within* climate models) to quantify *internal variability*.

Lastly, *dose-response function uncertainty* is quantified by using the full distribution of global GDP impacts, including different Monte Carlo draws for dose-response function parameters, and calculating the variance between parameter draws:

$$Var_b\left(E[\delta^C_{t,m,r,s,b}|GWL, b]\right) \quad \text{(S24)}$$

This approach is similar to Schwarzwald and Lenssen (2022)[23], who calculate model uncertainty and internal variability using only results for RCP8.5 and then calculate scenario uncertainty as the variance between RCP scenarios.

Absolute variance components and coefficients of variation

Variance shares in Figure 2c–d inform about the relative importance of uncertainty drivers but not about the magnitude of uncertainty (which is instead captured by the distribution and error bars in Figure 2a–b). Therefore, Figure S8 displays the absolute variance (expressed in basis points squared to facilitate readability). Colored bars represent the amount of variance attributed to each uncertainty driver. The grey diamond denotes the total variance, which can be slightly higher in case of interactions between variance components not captured by our main approach for variance decomposition. Omitted interaction terms can arise because the first decomposition step (into model uncertainty and internal variability) is carried out for a distribution that includes no dose-response function uncertainty (see the previous section on mathematical expressions). Figure S8 shows that for higher warming levels, the variance of GDP impacts increases for all climate indicators except for day-to-day temperature variability at +2°C. Therefore, declining *relative* variance shares in Figure 2 do not imply an *absolute* reduction in uncertainty. In addition, we note that differences between attributed variance (the combined bars) and total variance (grey diamond) are negligible for total GDP impacts and annual temperature damages, indicating that omitted interactions of uncertainty drivers play a less important role here. In contrast, such interactions are sizable for (some) variability and extremes.

While the absolute variance of GDP impacts is highest for annual mean temperature damages, this primarily reflects that these damages have a much higher magnitude than the direct impacts of variability and extremes. To account for this, Figure S9 shows the coefficient of variation of GDP impacts, calculated as the ratio of the standard deviation to the absolute value of the mean, for each global warming level and climate indicator. Relative to the mean impact, the effects of annual precipitation and variability and extremes are much more uncertain than annual mean temperature damages. However, uncertainty relative to mean impacts decreases with higher warming levels as mean impacts grow. Notably, the very high coefficients of variation for variability and extremes and monthly precipitation deviation are partially driven by the fact that average global GDP impacts (i.e., the denominator of the coefficient of variation) are near-zero for certain warming levels.

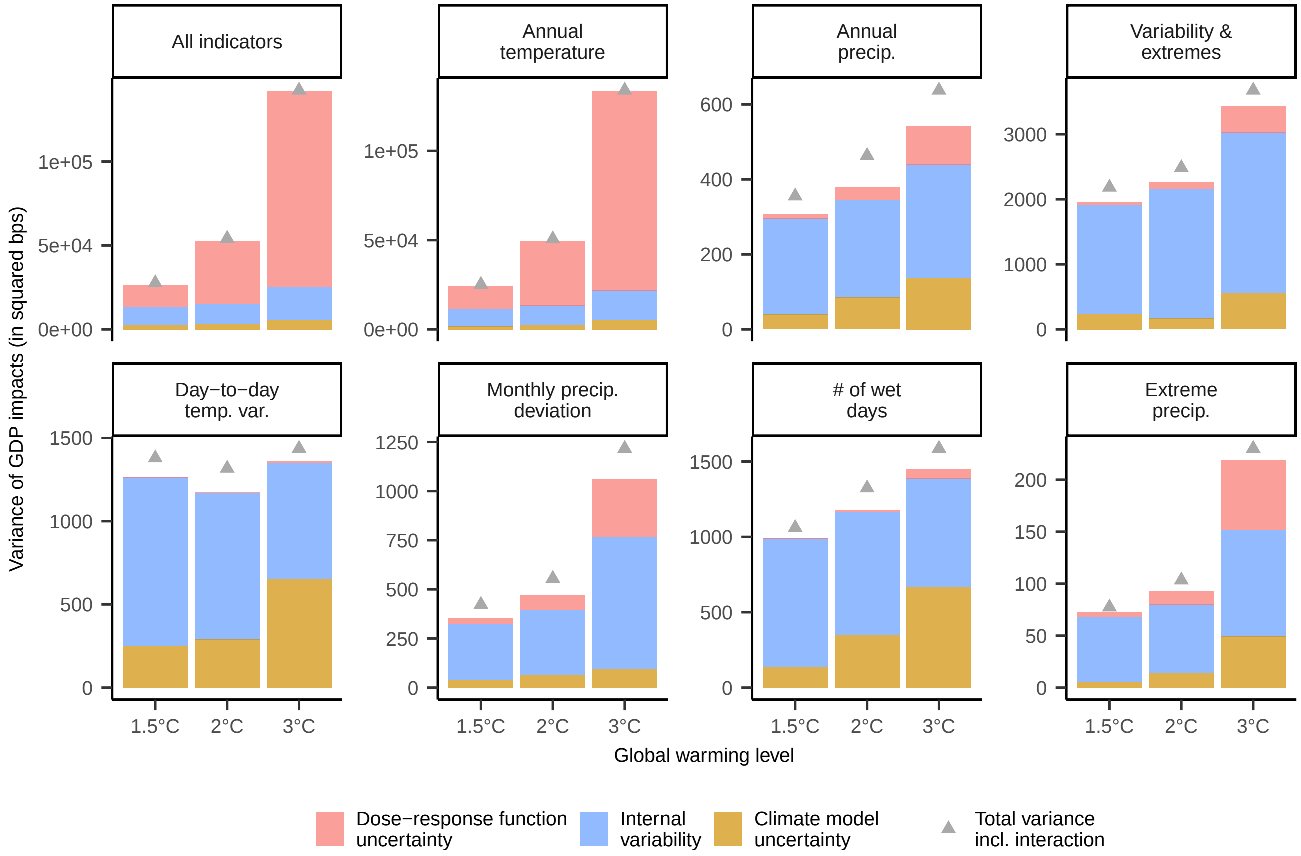

Figure S8. Variance decomposition of global GDP impacts in absolute terms based on our main approach

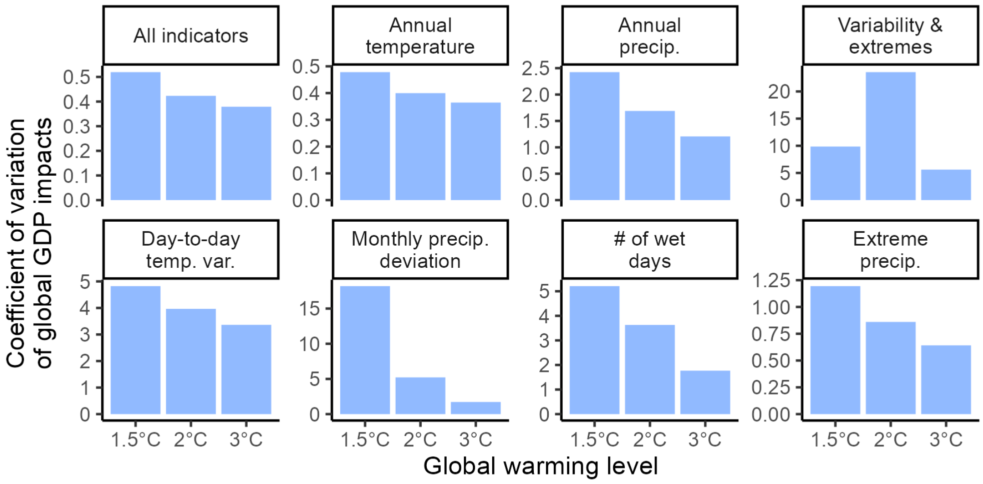

Figure S9. Coefficient of variation of global GDP impacts by global warming level and climate indicator

Using an alternative approach to decompose variance in GDP impacts

As Figure S8 shows above, our main approach for variance decomposition can omit interactions and implicitly assumes that uncertainty drivers are orthogonal[52]. In addition, results can potentially depend on the order in which variance drivers are considered. Therefore, we use an alternative approach for variance decomposition by Hsiang et al. (2017)[24] as a robustness check, which makes interaction terms explicit and is order-independent. The basic principle is to fix all uncertainty drivers except for one at central, median-like values and attribute the remaining marginal variance to the uncertainty driver that is not held constant. Using the notation introduced above, we can decompose the variance in global GDP impacts as follows:

$$\begin{aligned} Var(\delta^C_{t,m,r,s,b}|GWL) =& Var(\delta^C_{t,m,r,s,b}|GWL, m = m_0, t = t_0, s = s_0, r = r_0) + Var(\delta^C_{t,m,r,s,b}|GWL, m = m_0, b = b_0) + \\ & Var(\delta^C_{t,m,r,s,b}|GWL, t = t_0, s = s_0, r = r_0, b = b_0) + \Lambda \end{aligned} \tag{S25}$$

where all variables with a zero subscript are central, median-like values, and the first, second, and third terms capture the marginal variance due to dose-response function parameter draws (across values of b), internal variability (across values of r, t and s), and CMIP6 models (across values of m), respectively. Note that by conditioning on global warming levels, different scenarios still produce similar climatic outcomes, and hence, we subsume variation across s in internal variability. Λ is calculated as the residual between the sum of marginal variances and the total variance and hence accounts for the interaction of the different uncertainty drivers considered. Depending on how uncertainty drivers interact and the central values are chosen, Λ can be positive, negative, or zero.

We choose the central, median-like values for the variance decomposition as follows: b_0 is the dose-response function parameter draw that across all CMIP6 models and scenario-(realization-)years produces the median GDP impact for a given climate indicator C at +3°C of global warming. For each CMIP6 model, we set s_0 and t_0 (and, for large ensembles, r_0) to the scenario-(realization-)year that yields the median GDP impact for a given global warming level and climate indicator. Note that scenario-(realization-)years vary across warming levels such that we cannot choose s_0, r_0, and t_0 solely based on the +3°C warming level.

A particular challenge is to select the median-like model because the GDP impacts of multiple climate indicators and warming levels are considered. Figure S10 shows the mean GDP impact by CMIP6 model at +3°C of global warming for impacts from all climate indicators, as well as for separate impacts from each climate indicator. For instance, the CMCC-CM2-SR5 model is near-median for total GDP impacts at +3°C, but it is rather atypical with respect to variability and extremes. Therefore, we show results for four different central models: i) KACE-1-0-G (Figure S14) as it produces median damages for all indicators combined and near-median results for variability and extremes indicators overall (despite relatively high damages for extreme precipitation); ii) EC-Earth3-Veg (Figure S13) because it produces median damages for annual temperature damages and near-median outcomes for variability and extremes; and iii) the two large ensembles included in our analysis, MPI-ESM1-2-LR (Figure S12) and CESM2 (Figure S11), because using them as the central model means that variations across ensemble members are also reflected in our estimates of internal variability. Note that varying the central, median-like model m_0 does not alter the third term in Equation (S25), which is why the share of climate model uncertainty does not vary across Figures S11–S14.

Across the results in Figure 2 in the main manuscript and these four figures, dose-response function uncertainty consistently accounts for the highest variance share for total and annual temperature damages, whereas internal variability accounts for the highest share for annual precipitation impacts and the combined impacts of variability and extremes. In addition, climate model uncertainty accounts for a comparable or larger variance share than dose-response function for all variability and extremes—except for monthly precipitation deviation and for extreme precipitation at +3°C. Across all decomposition results, the share of climate model uncertainty decreases in the global warming level for annual temperature damages, whereas it increases for all indicators of variability and extremes. However, we note that the approach following Hsiang et al. (2017)[24] can produce extensive interaction terms with signs that vary between different central models, particularly for monthly precipitation deviation and extreme precipitation.

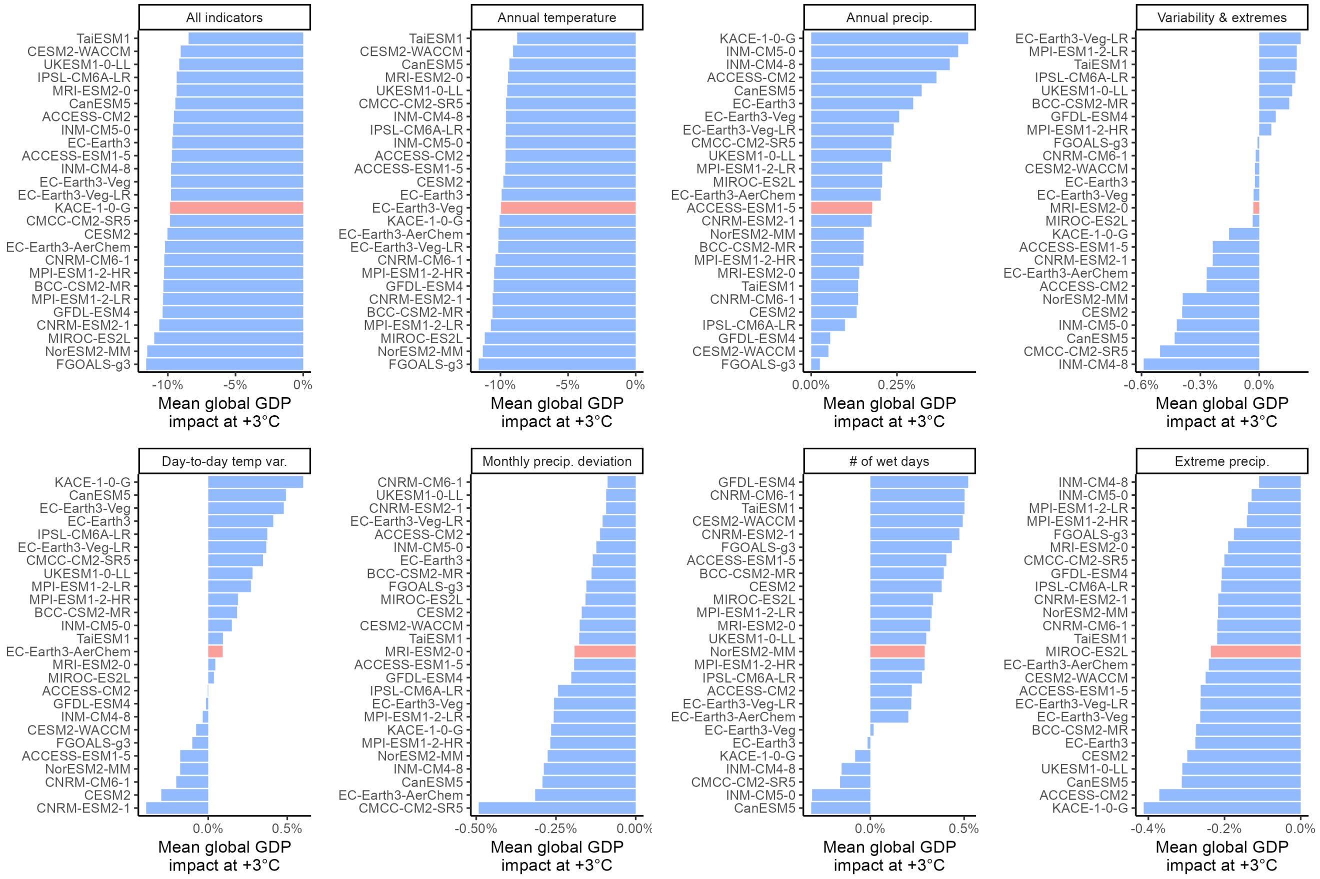

Figure S10. Average global GDP impact by CMIP6 model for different climate indicators at +3°C global warming (median model in red)

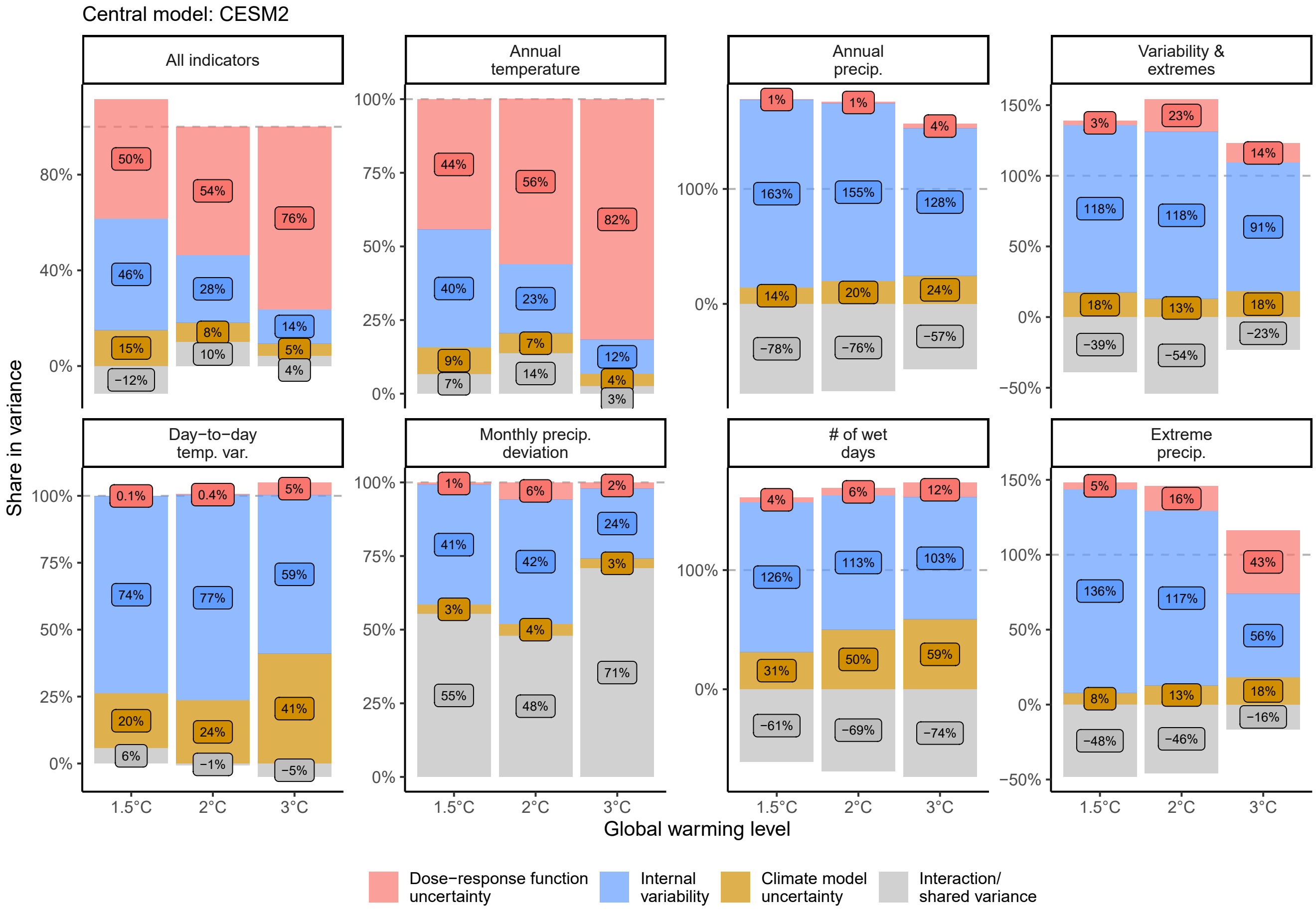

Figure S11. Variance decomposition of global GDP impacts following Hsiang et al. (2017)[24], using the CESM2 large ensemble as the central model

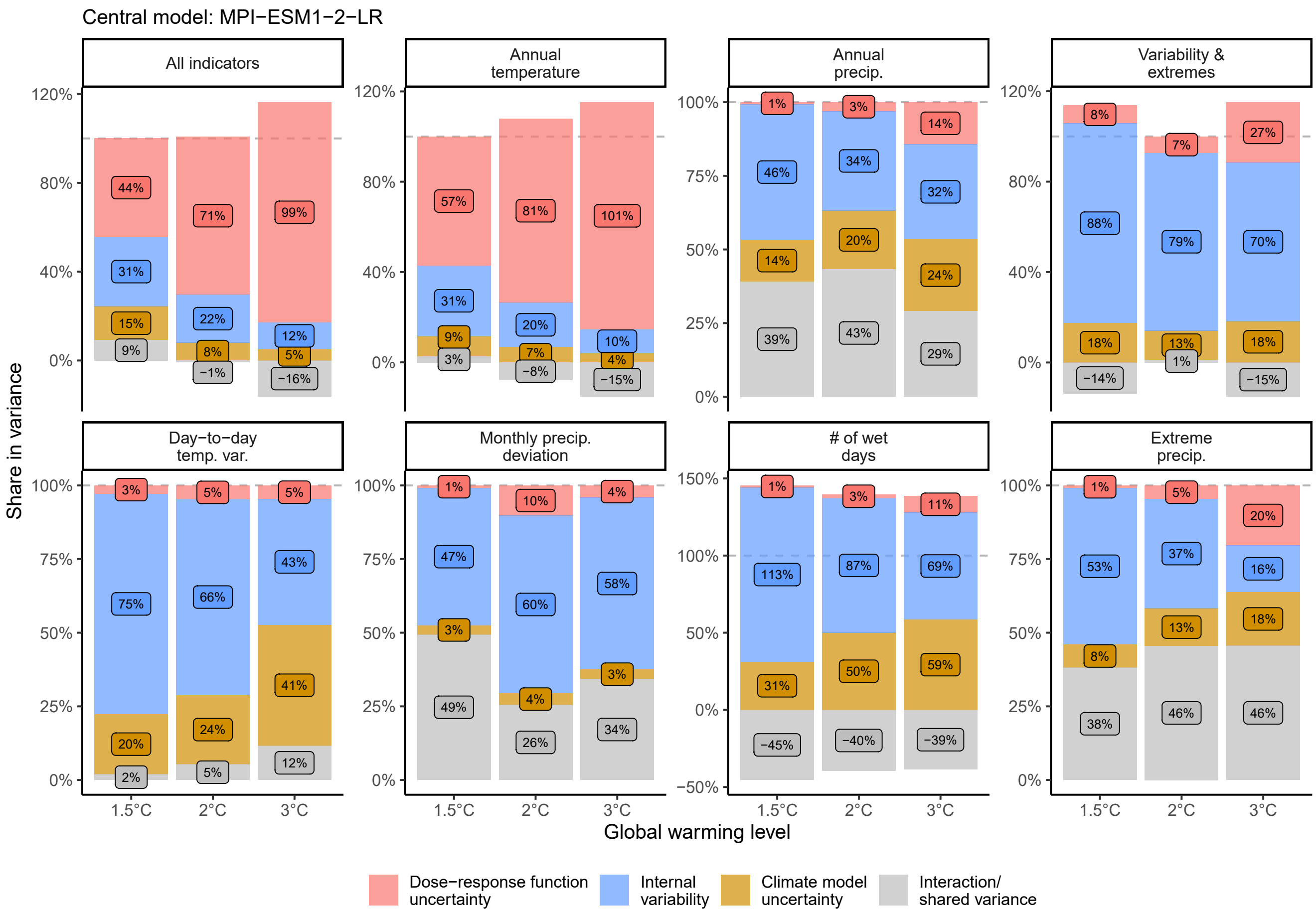

Figure S12. Variance decomposition of global GDP impacts following Hsiang et al. (2017)[24], using the MPI-ESM1-2-LR large ensemble as the central model

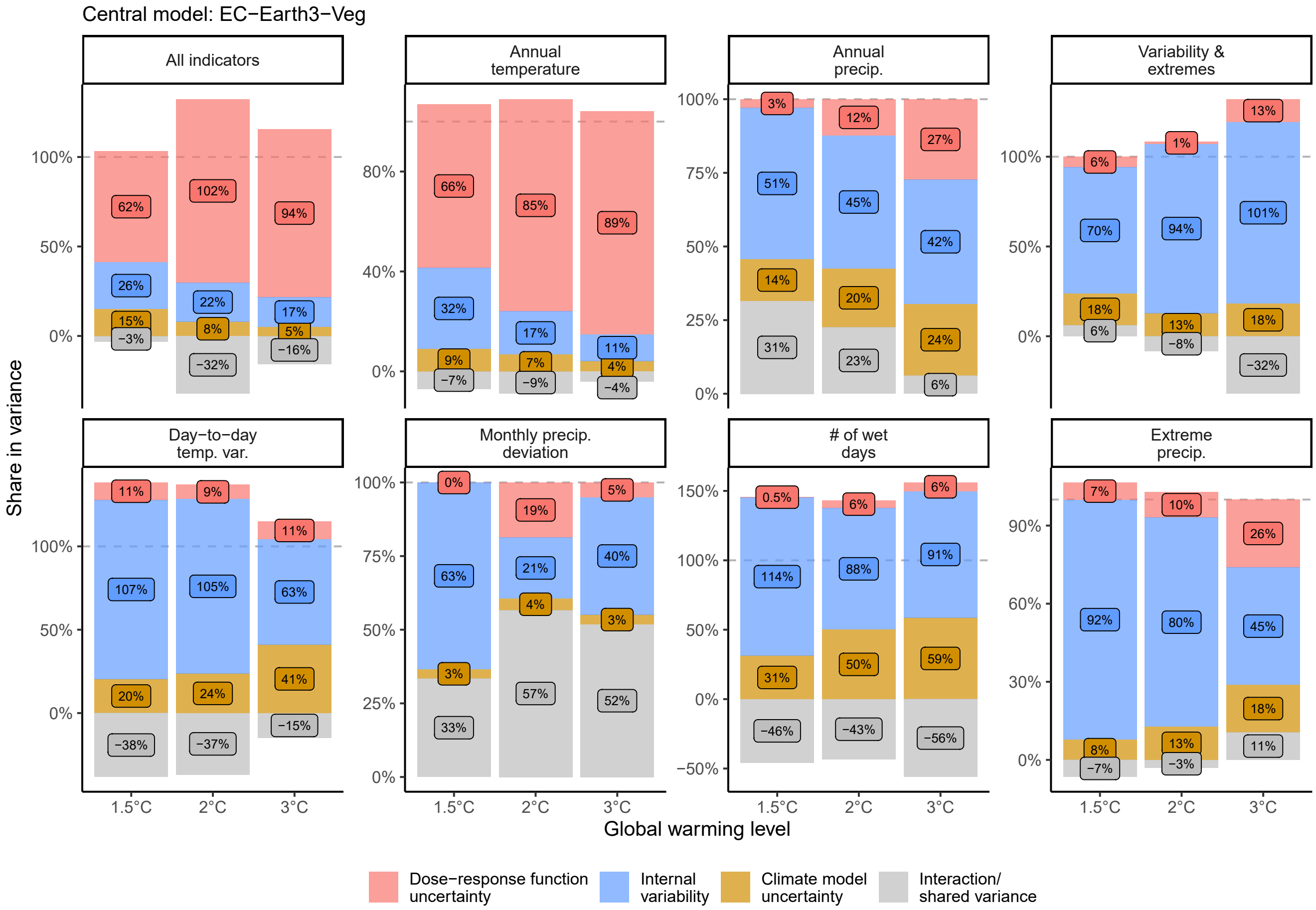

Figure S13. Variance decomposition of global GDP impacts following Hsiang et al. (2017)[24], using EC-Earth3-Veg as the central model

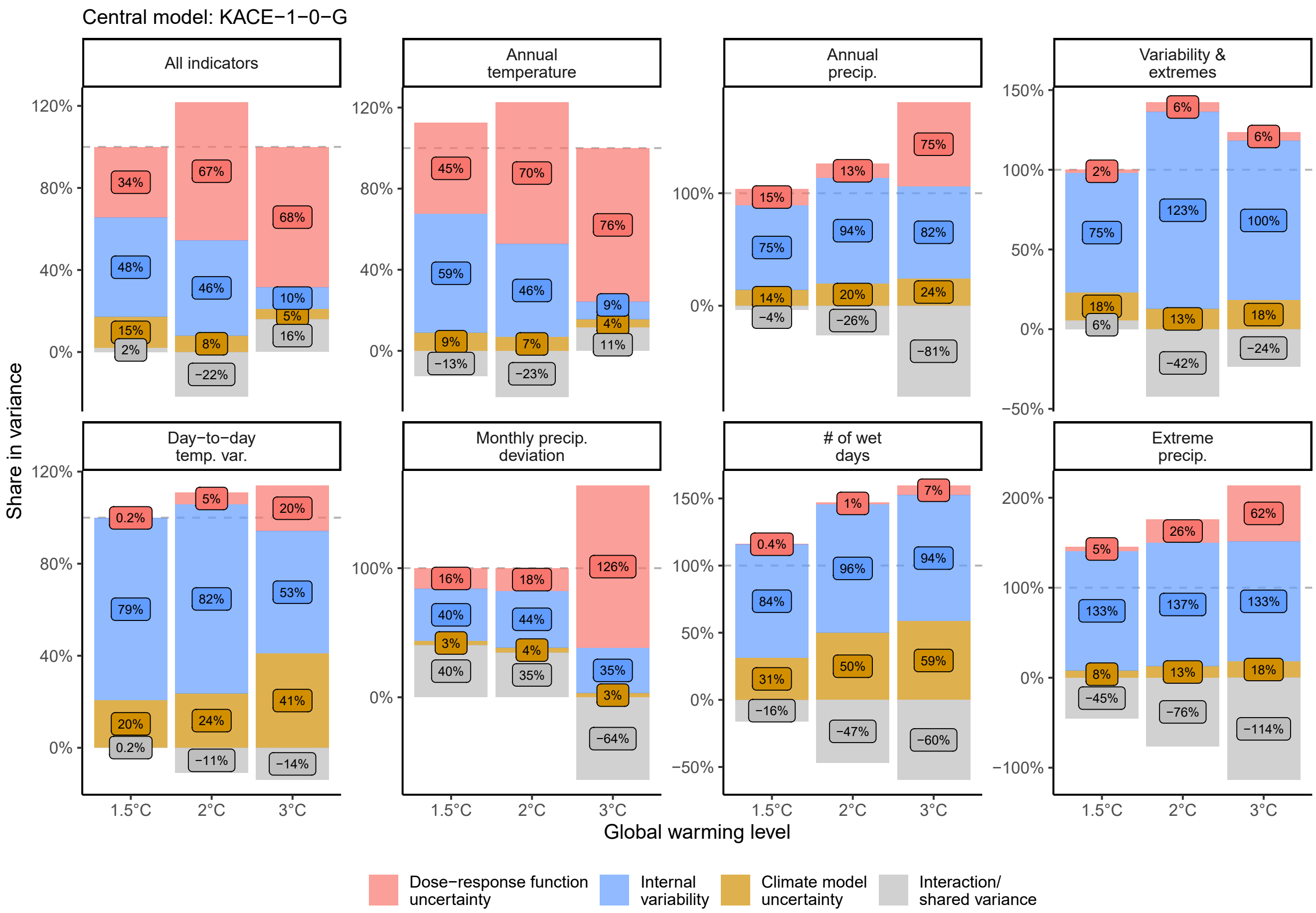

Figure S14. Variance decomposition of global GDP impacts following Hsiang et al. (2017)[24], using KACE-1-0-G as the central model

Appendix E - Additional results on the inclusion of large ensembles

While our analysis covers a wide range of climate models, a potentially important limitation is that due to computational limitations, we use only one model run for all models in our sample, with two notable exceptions. For CESM2, we use the entire large ensemble under SSP3-7.0, and for MPI-ESM1-2-LR, we use the first 30 realizations of the large ensemble under SSP3-7.0. We use large ensembles for these two models because CESM2 is a much-used model with a longstanding history of large ensemble analysis, while MPI-ESM1-2-LR exhibits a relatively good model performance[53]. However, when calculating summary statistics of our GDP impact distribution, we weight each climate model inversely such that all models have the same sampling probability. As a result, all model runs of a large ensemble combined have the same weight in our overall results as a single run from any of the remaining climate models. This serves to prevent the two large ensembles from dominating our overall results. However, two potential concerns concerning our main results are that by not including large ensembles for more models, we may underestimate internal variability and rare tail risk events that only occur for a few runs of a given model.

To explore the first concern, we use the two large ensembles included in our analysis to compare the internal variability of individual ensemble members, conditional on a given global warming level, with the internal variability of the entire ensemble. To do so, we use point estimates for the dose-response function (thus removing dose-response function uncertainty) and consider each large ensemble individually (thus removing climate model uncertainty), such that the remaining variance in GDP impacts is consistent with our definition of internal variability given in the **Methods** section of our main manuscript. Figure S15 illustrates the results where each grey dot represents the internal variability (in squared basis points) of an individual ensemble member, and the red dot and boxplot denote the mean as well as the median and upper/lower quartiles. By contrast, the blue triangle represents the internal variability calculated for the entire large ensemble. The first takeaway is that, somewhat trivially, some ensemble members exhibit considerable inter-annual variability, which exceeds both the average across ensemble members and the internal variability of the entire ensemble. Conversely, some ensemble members have an exceptionally low inter-annual variability. Therefore, if a single run is drawn randomly from a large ensemble, one might overestimate or underestimate the true internal variability of the model, potentially by a considerable amount. On average, however, the internal variability of a single ensemble member is nearly identical for annual temperature damages and only marginally smaller for variability and extremes. Decomposing the entire ensemble's internal variability into variance between and within ensemble members confirms that the latter (i.e., inter-annual variability) dominates internal variability (see Figure S16). Assuming that the two large ensembles considered here are not systematically different from the ones not included in our analysis, this means that if one repeats these single random draws for many different models, such random discrepancies should cancel out to some extent. For variability and extremes, internal variability is likely underestimated even then. However, based on Figure S15, the discrepancy should not be large enough to change the conclusions in our main manuscript—particularly because we already identify internal variability as the key uncertainty driver for variability and extremes.

To explore a potential underestimation of tail risks, we compare the distribution of global GDP impacts for a single run of a large ensemble with the distribution of the entire ensemble and with the (hypothetical) "All models (single runs)" distribution that features only single runs from the large ensembles and all other models. Since values for the large ensembles are only projected for SSP3-7.0, we also limit this hypothetical distribution to SSP3-7.0 runs to make results more comparable, which reduces the total number of climate models from 33 to 28. In addition, we show results only for point estimates of dose-response functions for the sake of readability and to focus on *climatic* tail risks. By focusing on one scenario and dose-response function parameter draw, a single climate model run produces exactly 20 projections for global GDP impacts per global warming level (since global warming level windows cover 20 years). Figure S17 shows the resulting distribution of global GDP impacts at +3°C by climate indicator for three distributions: i) the one obtained from a single run of the CESM2 large ensemble (r1i1p1f1); ii) the one obtained from the entire CESM2 large ensemble; and iii) the "All models (single runs)" distribution obtained by using only a single run for all climate models incl. the large ensembles. While the distributional range of annual temperature damages (and hence total damages) of the CESM2 large ensemble is covered by the "All models (Single runs)" distribution, tails for annual precipitation and variability and extremes are more pronounced in the large ensemble. However, this is primarily because CESM2 systematically produces much more pessimistic results regarding the global impact of day-to-day temperature variability than most other climate models in our sample (see Figure S10). By contrast, distribution tails for the remaining variability and extremes indicators at +3°C are roughly comparable between the "All models (Single runs)" distribution and the CESM2 large ensemble. When repeating the same analysis for the MPI-ESM1-2-LR large ensemble (see Figure S18), we again find that the "All models (Single runs)" distribution broadly covers tails of annual temperature damages. In addition, the lower tails for variability and extremes based on the large ensemble are only slightly more pronounced than the tails of the "All models (Single runs)" distribution in the case of monthly precipitation deviation, for which MPI-ESM1-2-LR can produce relatively large values compared to other climate models.

Taken together, Figures S17–S18 suggest that including large ensembles increases distribution tails at most by approximately 1%-pt (for CESM2 and day-to-day temperature variability; see Figure S17). However, we note that aggregating results from subnational regions to the global economy can mask more substantial changes in tail risks at the country or region level. Indeed,

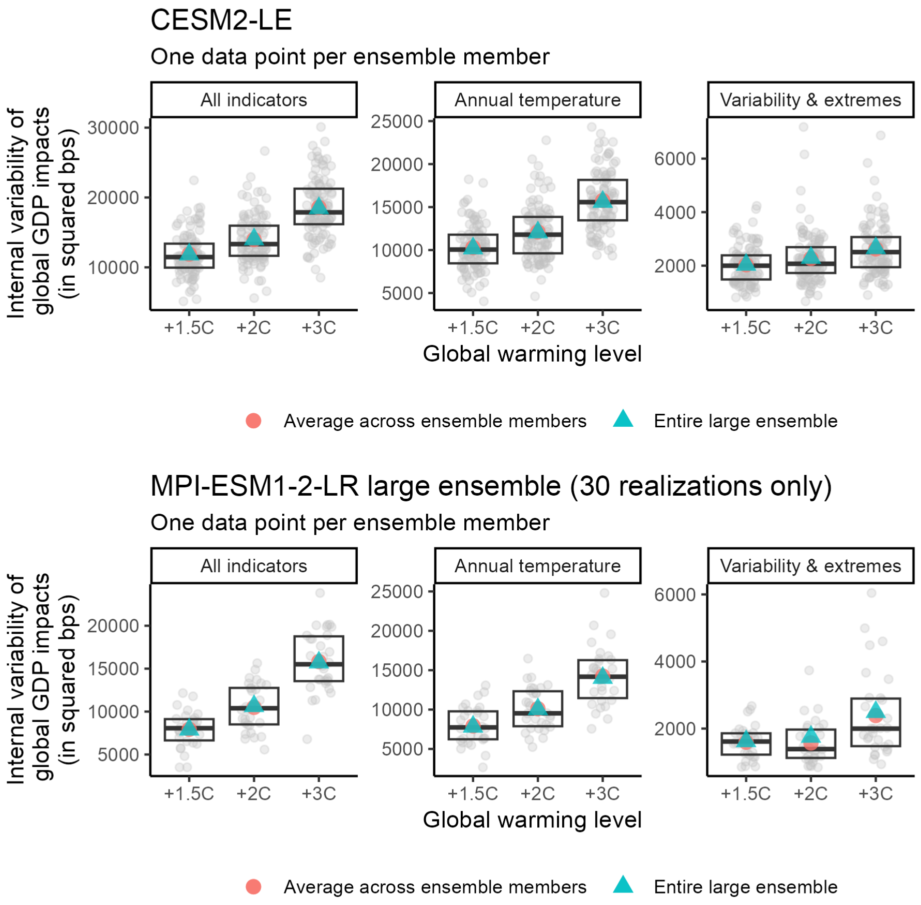

Figure S15. Internal variability of global GDP impacts of large ensemble members. Boxplot hinges denote the upper and lower quartile

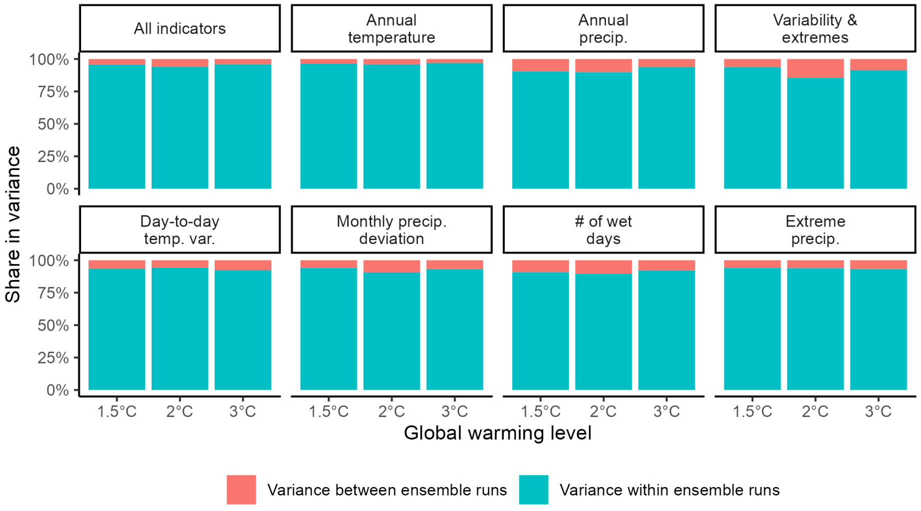

Figure S16. Variance of global GDP impacts at +3°C between and within MPI-ESM1-2-LR ensemble members. Calculated equivalent to the decomposition of variance into model uncertainty and internal variability by calculating variance between and within ensemble members based on point estimates for dose-response function parameters (i.e., abstracting from dose-response function uncertainty).

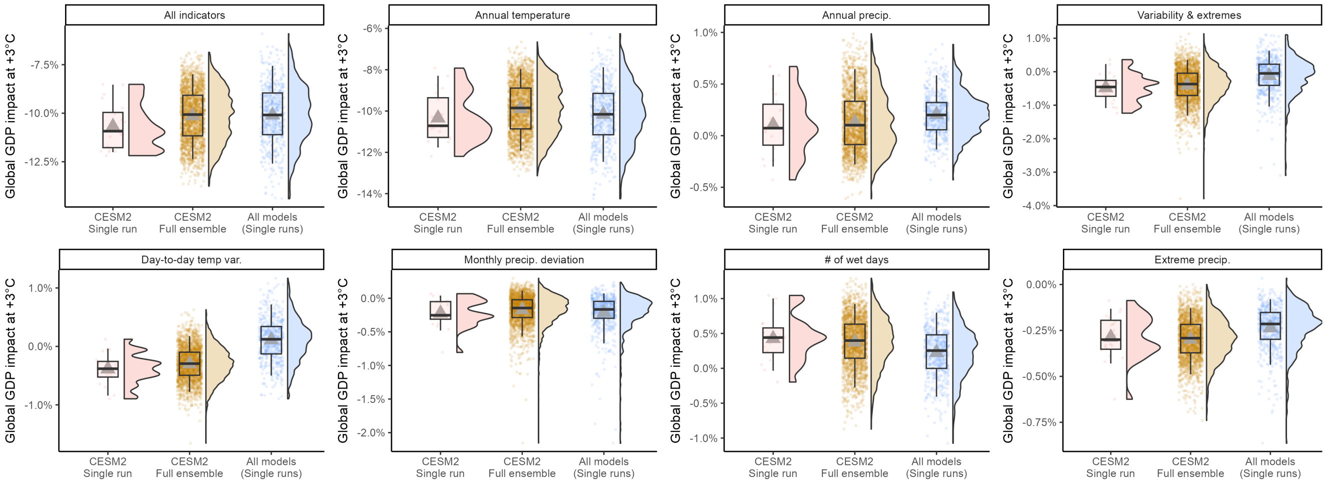

Figure S17. Distribution of global GDP impacts at +3°C of global warming for the CESM2 large ensemble compared to a distribution using all climate models in our sample with only one run per model. Grey triangles denote distribution means, boxplot hinges and whiskers denote upper/lower quartiles and 5th/95th percentiles, respectively.

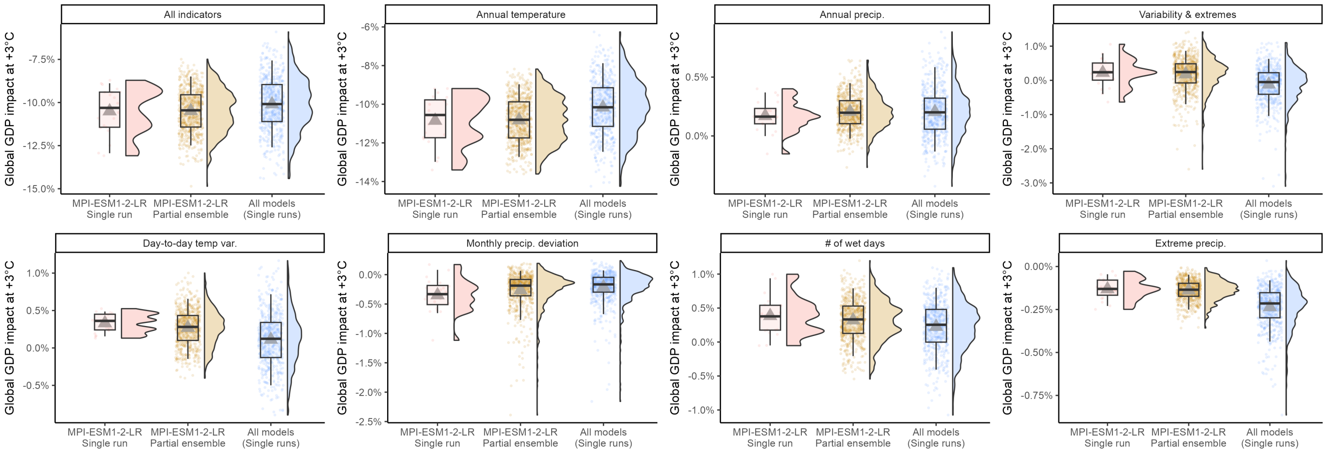

Figure S18. Distribution of global GDP impacts at +3°C of global warming for the 30 realizations from the MPI-ESM1-2-LR large ensemble compared to a distribution using all climate models in our sample with only one run per model. Grey triangles denote distribution means, boxplot hinges and whiskers denote upper/lower quartiles and 5th/95th percentiles, respectively.

Figure 1c in the main manuscript (for extreme precipitation in the United States) demonstrates that the distribution tails for the two large ensembles (CESM2 and MPI-ESM1-2-LR) are much more pronounced than for other models, while no similar anomaly can be observed for the 90th percentile of GDP impacts. These results illustrate that using large ensembles is important for correctly identifying tail risks when it comes to the GDP impacts of variability and extremes in particular and even more so for capturing risks related to rare compound events[54] not represented by our dose-response functions, which consider each impact channel separately. Therefore, introducing more large ensembles into our analysis would likely increase the tail risks identified in the main manuscript for these climate indicators, particularly in region- or country-specific analyses.

Nevertheless, if researchers face computational constraints and hence a trade-off between including more ensemble members and adding more climate models, our results suggest that using a large number of different climate models can be an (imperfect) substitute to explore tail risks and, in addition, provides a better understanding of model uncertainties, which play an important role for variability and extremes (see Figure 2 in the main manuscript).

Appendix F - Indicative results on the inclusion of heat and drought measures

Notably, our main results do not explicitly project the economic impacts of other climatic extremes, such as heat or droughts. The reason for this is that so far, individual studies have focused on particular sets of climate indicators such as annual precipitation[55, 56], intra-annual precipitation patterns[2], temperature variability[28, 57], or extreme heat[22]. However, a peer-reviewed regression-based analysis that simultaneously accounts for all these indicators is missing. Due to the strong correlations between different climate indicators, controlling for one indicator when estimating another one's dose-response function can affect results substantially[58], as Figure 4 illustrates for the temperature dose-response function. Therefore, piecing together dose-response functions from different studies risks introducing considerable bias and potential double-counting.

To avoid this, our main results rely on the main specification by Kotz et al. (2022)[2] (hereafter KLW), which combines six climate indicators but does not explicitly feature extreme heat or droughts. Assessing the likely impact of this potential omission, we first note that the robustness checks by KLW cover two measures of heat extremes: the number of days exceeding the 99th or 99.9th percentile of the location-specific historical distribution (in their Table S7). For warmer regions, they find a significant negative effect on income growth for both measures when interacted with annual mean temperature, which in turn reduces the absolute magnitude and the significance of the coefficients related to annual mean temperature. However, these two heat measures are motivated primarily as a robustness check for the identified effects of intra-annual precipitation patterns rather than as a well-grounded measure of heat wave impacts. By contrast, Callahan and Mankin (2022)[22] (hereafter CM) specifically investigate the effects of heat, measured as a given year's average daily maximum temperature throughout the hottest five-day period ("Tx5d"), on subnational income. In their regression, they control for annual mean temperature, temperature variability, the average annual cycle of temperature, and annual precipitation. The analysis also features a comprehensive battery of robustness checks, including alternative measures of heat such as extreme degree days or the average temperature of the year's hottest month. Notably, the income per capita data and the temporal and geographical resolution used in CM to estimate their heat dose-response function are the same as in KLW. While CM, unlike KLW, adjust income per capita growth for inflation, we explain in Appendix **C** that this step does not affect results in a regression that uses the first difference of log-transformed output as a dependent variable and features year-fixed effects.

Therefore, we calculate Tx5d based on ERA5 for each grid cell between 1979–2019 and aggregate it to ADM1 regions with the same area weights as for our main climate indicators to obtain indicative results on whether including heat measures in the regression model by KLW would change the results we present in the main manuscript. However, this approach still suffers from two important caveats. First, while the main specifications from CM and KLW each have passed peer review and various robustness checks, this does not hold for their *ad hoc* combination. Replicating all of these robustness checks (e.g., using alternative climate data sets and measurement approaches, assessing spatial correlations, or exploring seasonal and sectoral heterogeneities) would go beyond the scope of our study. Second, our analysis uses area-weighted aggregation approaches consistent with KLW, whereas CM deploy population-weighted climate indicators. As a result, the Tx5d measure we calculate is very similar ($r = 0.93$) but not identical to the one used by CM and, therefore, produces (slightly) different regression coefficient estimates. For these two reasons, we see the inclusion of Tx5d as a complementary and more preliminary analysis with a substantially weaker foundation than our main results.

Table S9 shows regression results for the following specifications: i) the main specification by KLW underlying our main results; ii) adding Tx5d as a control variable; iii) adding Tx5d incl. an interaction term with annual temperature, following the implementation in the main specification by CM; iv) an alternative specification where we interact Tx5d with a region's long-term temperature average. We do not find a significant relationship between Tx5d and income per capita growth in our specification if it is added as a simple control variable (column 2) and the point estimate is, in fact, positive. This is consistent with a result reported by Kotz et al. (2021)[28] who explore the impacts of day-to-day temperature variability in the same sample as KLW. As part of their robustness checks (see their Table S1), they include heat stress days as a linear term using absolute thresholds at 25°C and 30 °C and report positive and non-significant coefficients. However, we obtain significant results and the negative growth impact for warmer countries reported by CM when we interact Tx5d with annual mean temperature (column 3). The coefficients' sign and significance are consistent with the main specification by CM, although our coefficient magnitudes are slightly lower (0.0073 and -0.00049 instead of the 0.0087 and -0.00063 reported by CM; see their Table S1). The results are also consistent with the robustness check by KLW, who report a positive coefficient for their respective heat measure and a negative coefficient for the interaction with annual mean temperature (in their Table S7). As reported by CM (in their Table S2), interacting Tx5d with the long-term average of annual mean temperature instead of the current year's temperature does not change these findings (column 4), addressing potential concerns that the significant impact of the interaction term stems primarily from annual temperatures and not from Tx5d.

However, interacting Tx5d and (annual or long-term) mean temperature renders the relevant annual temperature terms insignificant and alters the magnitudes of their coefficients. Therefore, CM conclude that at least some of the damages conventionally attributed to annual mean temperatures are, in fact, driven by heat extremes. The results in Table S9 align with this interpretation and show that the substantial overlap between the annual temperature and Tx5d dose-response function

Table S9. Including heat measured by Tx5d following Callahan & Mankin (2022)

	Dependent variable:			
	Subnational income per capita growth			
	(1)	(2)	(3)	(4)
D.MeanTemp	9.6e-04	6.2e-04	6.4e-03	-2.4e-03
	(4.8e-03)	(4.8e-03)	(5.8e-03)	(4.9e-03)
L.D.MeanTemp	-2.3e-03	-2.3e-03	6.2e-04	-3.8e-03
	(5.9e-03)	(5.9e-03)	(6e-03)	(6.1e-03)
D.MeanTemp:MeanTemp	-1.1e-03**	-1.1e-03**	-7e-04	-7e-04
	(5.1e-04)	(5.1e-04)	(5e-04)	(5.1e-04)
L.D.MeanTemp:L.MeanTemp	-6.5e-04	-6.7e-04	-4.4e-04	-4.5e-04
	(5.2e-04)	(5.3e-04)	(5.4e-04)	(5.6e-04)
Day-to-day temp. var.	-5.8e-02***	-5.9e-02***	-6.5e-02***	-6e-02***
	(0.016)	(0.017)	(0.017)	(0.016)
Annual precip.	5.8e-05**	5.8e-05**	5.5e-05**	5e-05*
	(2.7e-05)	(2.7e-05)	(2.8e-05)	(2.9e-05)
Annual precip. sq.	-3.8e-09**	-3.8e-09**	-3.7e-09**	-3.4e-09**
	(1.7e-09)	(1.7e-09)	(1.7e-09)	(1.7e-09)
Monthly precip. deviation	0.017*	0.017*	0.019*	0.021*
	(0.01)	(9.9e-03)	(0.01)	(0.011)
Monthly precip. deviation sq.	-2.8e-02***	-2.8e-02***	-2.7e-02***	-2.8e-02***
	(9.9e-03)	(9.9e-03)	(9.7e-03)	(0.01)
No. of wet days	-1.3e-03**	-1.3e-03**	-1.3e-03**	-1.3e-03**
	(5.3e-04)	(5.5e-04)	(5.4e-04)	(5.5e-04)
No. of wet days sq.	1.1e-06	1.1e-06	9e-07	7.4e-07
	(1.2e-06)	(1.2e-06)	(1.2e-06)	(1.2e-06)
Extreme precip.	-3.7e-04***	-3.7e-04***	-3.9e-04***	-3.8e-04***
	(8.7e-05)	(8.8e-05)	(8.4e-05)	(8.7e-05)
Extreme precip.:MeanTemp	1.3e-05***	1.3e-05***	1.4e-05***	1.4e-05***
	(3.7e-06)	(3.7e-06)	(3.7e-06)	(3.7e-06)
Tx5d		1.1e-03	7.3e-03**	8.5e-03***
		(2.1e-03)	(3.2e-03)	(2.9e-03)
Tx5d:MeanTemp			-4.9e-04**	
			(2.4e-04)	
Tx5d:MeanTempLongTerm				-7.3e-04**
				(2.9e-04)
Observations	30121	30121	30121	30121
R^2	0.014	0.014	0.016	0.016
Adjusted R^2	-4.1e-02	-4.1e-02	-4e-02	-4e-02
Note:	St.err. clustered at country level. *p<0.1; **p<0.05; ***p<0.01			

remains when one additionally controls for extreme precipitation and other rainfall patterns. This is perhaps unsurprising given the strong positive correlation of the two indicators in the KLW sample ($r = 0.58$) and the fact that the hottest 5-day period of the year, due to its outlier characteristic, by definition, has a disproportionate effect on the annual average.

If annual mean temperature damages track heat impacts to some extent, then projecting GDP losses due to heat separately should lead to a corresponding decrease in annual temperature damages. To confirm this, we calculate Tx5d for all of our climate models and project GDP impacts using the point estimates from column 3 in Table S9 (i.e., the implementation corresponding to the main specification by CM) for all climate indicators. Note that since calculating results for all model-realization-scenario pairings and Monte Carlo draws is very expensive computationally, we do not use Monte Carlo draws for the dose-response functions, and we only use the first realization of the MPI-ESM1-2-LR large ensemble. Moreover, the daily maximum temperature is not available in the CMIP6 repository used here for the CESM2 large ensemble and for CESM2-WACCM and CMCC-CM2-SR5, so we exclude these models. Results under +3°C of global warming are presented in Figure S19 and confirm that accounting for extreme heat re-attributes almost half of the annual mean temperature damages to heat extremes, such that total damages by all climate indicators are now driven by the sum of heat and annual temperature damages rather than just the latter. Therefore, our results align with the conclusion by CM that heat impacts at least partially drive damages conventionally attributed to annual temperature. At the same time, global losses from the remaining climate indicators remain remarkably similar, such that our conclusions in the main manuscript concerning the smaller magnitude of temperature variability and precipitation patterns and the sign of impacts (negative for extreme precipitation and monthly precipitation deviation, positive for day-to-day temperature variability and number of wet days) are robust to including Tx5d. However, complementary research is needed to disentangle the effects of heat wave events and broader temperature shifts better.

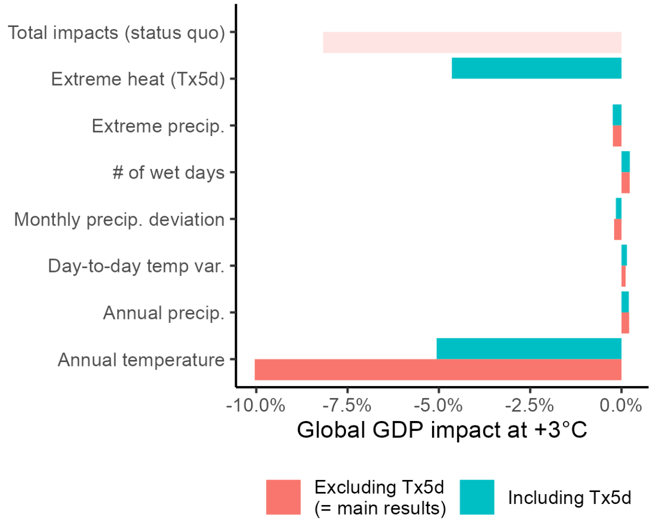

Figure S19. Mean global GDP impact by impact channel using the dose-response point estimates from Table S9, column 3 (blue) and according to our main results (red). Unlike our main results, results for including Tx5d are based on a subset of model runs excl. CESM-LE, CESM2-WACCM, CMCC-CM2-SR5 and all ensemble members of MPI-ESM1-2-LR except r1i1p1f1 and use point estimates for dose-response functions to reduce computational requirements, such that red and blue bars are not fully comparable.

Regarding droughts, we first note that KLW interpret their findings that negative monthly precipitation deviations away from historical means reduce growth as indicative of drought damages. Furthermore, a pronounced drought will lower annual precipitation, translating to economic losses according to our main dose-response function (see Figure S7). Therefore, the dose-response functions for these two indicators may capture some drought impacts, similar to how annual temperature impacts seemingly capture heat wave effects at least partially. For a preliminary assessment of including droughts explicitly, we take the Palmer Drought Severity Index (PDSI) data from CM, which they use to investigate if droughts act as moderators of heat extremes, and include it into the main specification by KLW. Notably, this procedure is somewhat inconsistent because, unlike KLW, CM use population weighting. Hence, the PDSI values for subnational regions rely on different weighting schemes than the remaining climate indicators. In addition, PDSI values are not available for about 2,000 region-year observations in the KLW sample, thus reducing the sample size when included. Results are shown in Table S10 and illustrate that the PDSI has no significant relationship with subnational income per capita growth as a linear term (column 2) or when being interacted with either annual temperature (column 3) or long-term average temperature (column 4). To ensure that inconsistent

weighting schemes do not primarily drive this finding, we merge CM's original, population-weighted Tx5d measure and re-run the specification in Table S9, column 3 again. The resulting coefficients (Table S10, column 5) are almost identical to the ones derived from our area-weighted Tx5d measure, suggesting that differences in weighting schemes have only a moderate effect on results.

At the same time, the lack of a significant relationship between PDSI and income per capita growth might simply speak to the shortcomings of PDSI as a comprehensive drought measure for economic impact assessments, for instance, due to its limited comparability across regions[59]. It might also be caused by some key heterogeneity not captured in our regression specification—for instance, if droughts reduce mainly agricultural output and hence play a much more critical role for economies with high agricultural intensity[56]—or by the lower suitability of top-down damage function estimations for this particular indicator since drought events are rare and have more dire impacts in poorest and hottest regions that are underrepresented in the subnational income data used by KLW and CM. Therefore, Table S10 should certainly not be taken as a definitive answer to the role of droughts for top-down damage estimates, and further research on their economic impacts is needed.

Table S10. Including drought measured by the Palmer Drought Severity Index (PDSI)

	Dependent variable:				
	Subnational income per capita growth				
	(1)	(2)	(3)	(4)	(5)
D.MeanTemp	9.6e-04	9.2e-04	3.6e-04	4.8e-04	6.1e-03
	(4.8e-03)	(4.5e-03)	(4.5e-03)	(4.5e-03)	(5.4e-03)
L.D.MeanTemp	-2.3e-03	-2.7e-03	-3.1e-03	-3e-03	2.5e-04
	(5.9e-03)	(5.7e-03)	(5.6e-03)	(5.6e-03)	(5.7e-03)
D.MeanTemp:MeanTemp	-1.1e-03**	-1e-03**	-9.5e-04*	-9.6e-04*	-6.9e-04
	(5.1e-04)	(4.9e-04)	(5e-04)	(5e-04)	(5e-04)
L.D.MeanTemp:L.MeanTemp	-6.5e-04	-5.7e-04	-5.2e-04	-5.3e-04	-4.3e-04
	(5.2e-04)	(5.4e-04)	(5.2e-04)	(5.2e-04)	(5.5e-04)
Day-to-day temp. var.	-5.8e-02***	-5.6e-02***	-5.7e-02***	-5.7e-02***	-6.5e-02***
	(0.016)	(0.016)	(0.016)	(0.016)	(0.017)
Annual precip.	5.8e-05**	5.8e-05**	5.3e-05**	5.4e-05**	5.4e-05*
	(2.7e-05)	(2.8e-05)	(2.7e-05)	(2.6e-05)	(2.8e-05)
Annual precip. sq.	-3.8e-09**	-3.7e-09**	-3.5e-09**	-3.6e-09**	-3.7e-09**
	(1.7e-09)	(1.7e-09)	(1.6e-09)	(1.6e-09)	(1.7e-09)
Monthly precip. deviation	0.017*	0.014	0.015	0.015	0.019*
	(0.01)	(9.7e-03)	(0.01)	(0.01)	(9.9e-03)
Monthly precip. deviation sq.	-2.8e-02***	-2.6e-02**	-2.6e-02**	-2.6e-02**	-2.5e-02**
	(9.9e-03)	(0.01)	(0.01)	(0.01)	(0.01)
No. of wet days	-1.3e-03**	-1.4e-03**	-1.4e-03**	-1.4e-03**	-1.2e-03**
	(5.3e-04)	(5.7e-04)	(5.8e-04)	(5.8e-04)	(5.4e-04)
No. of wet days sq.	1.1e-06	1.1e-06	1.1e-06	1.1e-06	7.4e-07
	(1.2e-06)	(1.2e-06)	(1.2e-06)	(1.3e-06)	(1.2e-06)
Extreme precip.	-3.7e-04***	-3.9e-04***	-3.8e-04***	-3.9e-04***	-4e-04***
	(8.7e-05)	(9.3e-05)	(9.7e-05)	(9.8e-05)	(8.5e-05)
Extreme precip.:MeanTemp	1.3e-05***	1.4e-05***	1.4e-05***	1.4e-05***	1.4e-05***
	(3.7e-06)	(4e-06)	(4.2e-06)	(4.2e-06)	(3.6e-06)
PDSI (CM)		2.4e-03	5e-04	9.2e-04	
		(1.7e-03)	(3.4e-03)	(3.6e-03)	
PDSI (CM):MeanTemp			1.3e-04		
			(1.5e-04)		
PDSI (CM):MeanTempLongTerm				1e-04	
				(1.6e-04)	
Tx5d (CM)					7.6e-03***
					(2.8e-03)
Tx5d (CM):Tmean					-4.9e-04**
					(2e-04)
Observations	30121	28109	28109	28109	28109
R^2	0.014	0.014	0.014	0.014	0.016
Adjusted R^2	-4.1e-02	-4e-02	-4e-02	-4e-02	-3.9e-02
Note:	St.err. clustered at country level. *p<0.1; **p<0.05; ***p<0.01				

References

1. Batibeniz, F., Hauser, M., & Seneviratne, S. I. Countries most exposed to individual and concurrent extremes and near-permanent extreme conditions at different global warming levels. *Earth System Dynamics* **14**, 485–505 (2023). DOI: 10.5194/esd-14-485-2023.
2. Kotz, M., Levermann, A., & Wenz, L. The effect of rainfall changes on economic production. *Nature* **601**, 223–227 (2022). DOI: 10.1038/s41586-021-04283-8.
3. Masson-Delmotte, V. et al., eds. Climate change 2021: The physical science basis: Contribution of Working Group I to the Sixth Assessment Report of the Intergovernmental Panel on Climate Change. Cambridge, UK and New York, NY, USA: Cambridge University Press, (2021). DOI: 10.1017/9781009157896.
4. Zhang, X., Hegerl, G., Zwiers, F. W., & Kenyon, J. Avoiding inhomogeneity in percentile-based indices of temperature extremes. *Journal of Climate* **18**, 1641–1651 (2005). DOI: 10.1175/JCLI3366.1.
5. Zhang, X. et al. Indices for monitoring changes in extremes based on daily temperature and precipitation data. *WIREs Climate Change* **2**, 851–870 (2011). DOI: 10.1002/wcc.147.
6. Seneviratne, S. I. et al. Weather and climate extreme events in a changing climate. Ed. by Masson-Delmotte, V. et al. *Climate Change 2021: The Physical Science Basis*, 1513–1766. Cambridge, UK and New York, NY, USA: Cambridge University Press, (2021). DOI: 10.1017/9781009157896.013.
7. Coppola, E. et al. Climate hazard indices projections based on CORDEX-CORE, CMIP5 and CMIP6 ensemble. *Climate Dynamics* **57**, 1293–1383 (2021). DOI: 10.1007/s00382-021-05640-z.
8. Outten, S. & Sobolowski, S. Extreme wind projections over Europe from the Euro-CORDEX regional climate models. *Weather and Climate Extremes* **33**, 100363 (2021). DOI: 10.1016/j.wace.2021.100363.
9. Reale, M. et al. Future projections of Mediterranean cyclone characteristics using the Med-CORDEX ensemble of coupled regional climate system models. *Climate Dynamics* **58**, 2501–2524 (2022). DOI: 10.1007/s00382-021-06018-x.
10. Stocchi, P. et al. Non-hydrostatic Regcm4 (Regcm4-NH): Evaluation of precipitation statistics at the convection-permitting scale over different domains. *Atmosphere* **13**, 861 (2022). DOI: 10.3390/atmos13060861.
11. Kim, Y H., Min, S K., Zhang, X., Sillmann, J., & Sandstad, M. Evaluation of the CMIP6 multi-model ensemble for climate extreme indices. *Weather and Climate Extremes* **29**, 100269 (2020). DOI: 10.1016/j.wace.2020.100269.
12. Schwarzwald, K., Goddard, L., Seager, R., Ting, M., & Marvel, K. Understanding CMIP6 biases in the representation of the Greater Horn of Africa long and short rains. *Climate Dynamics* **61**, 1229–1255 (2023). DOI: 10.1007/s00382-022-06622-5.
13. Rossow, W. B., Mekonnen, A., Pearl, C., & Goncalves, W. Tropical precipitation extremes. *Journal of Climate* **26**, 1457–1466 (2013). DOI: 10.1175/JCLI-D-11-00725.1.
14. Pfahl, S., O'Gorman, P. A., & Fischer, E. M. Understanding the regional pattern of projected future changes in extreme precipitation. *Nature Climate Change* **7**, 423–427 (2017). DOI: 10.1038/nclimate3287.
15. Orlowsky, B. & Seneviratne, S. I. Elusive drought: Uncertainty in observed trends and short- and long-term CMIP5 projections. *Hydrology and Earth System Sciences* **17**, 1765–1781 (2013). DOI: 10.5194/hessd-9-13773-2012.
16. Mueller, B. & Seneviratne, S. I. Systematic land climate and evapotranspiration biases in CMIP5 simulations. *Geophysical Research Letters* **41**, 128–134 (2014). DOI: 10.1002/2013GL058055.
17. Zhu, H. et al. Does CMIP6 inspire more confidence in simulating climate extremes over China? *Advances in Atmospheric Sciences* **37**, 1119–1132 (2020). DOI: 10.1007/s00376-020-9289-1.
18. Zhu, Y Y. & Yang, S. Evaluation of CMIP6 for historical temperature and precipitation over the Tibetan Plateau and its comparison with CMIP5. *Advances in Climate Change Research* **11**, 239–251 (2020). DOI: 10.1016/j.accre.2020.08.001.
19. Hauser, M., Orth, R., & Seneviratne, S. I. Role of soil moisture versus recent climate change for the 2010 heat wave in western Russia. *Geophysical Research Letters* **43**, 2819–2826 (2016). DOI: 10.1002/2016GL068036.
20. Rasmijn, L. M. et al. Future equivalent of 2010 Russian heatwave intensified by weakening soil moisture constraints. *Nature Climate Change* **8**, 381–385 (2018). DOI: 10.1038/s41558-018-0114-0.
21. Vries, I. E. de, Sippel, S., Pendergrass, A. G., & Knutti, R. Robust global detection of forced changes in mean and extreme precipitation despite observational disagreement on the magnitude of change. *Earth System Dynamics* **14**, 81–100 (2023). DOI: 10.5194/esd-14-81-2023.
22. Callahan, C. W. & Mankin, J. S. Globally unequal effect of extreme heat on economic growth. *Science Advances* **8**, eadd3726 (2022). DOI: 10.1126/sciadv.add3726.

23. Schwarzwald, K. & Lenssen, N. The importance of internal climate variability in climate impact projections. *Proceedings of the National Academy of Sciences of the United States of America* **119**, e2208095119 (2022). DOI: 10.1073/pnas.2208095119.

24. Hsiang, S. et al. Estimating economic damage from climate change in the United States. *Science* **356**, 1362–1369 (2017). DOI: 10.1126/science.aal4369.

25. Burke, M., Hsiang, S. M., & Miguel, E. Global non-linear effect of temperature on economic production. *Nature* **527**, 235–239 (2015). DOI: 10.1038/nature15725.

26. Kalkuhl, M. & Wenz, L. The impact of climate conditions on economic production: Evidence from a global panel of regions. *Journal of Environmental Economics and Management* **103**, 102360 (2020). DOI: 10.1016/j.jeem.2020.102360.

27. Ricke, K., Drouet, L., Caldeira, K., & Tavoni, M. Country-level social cost of carbon. *Nature Climate Change* **8**, 895–900 (2018). DOI: 10.1038/s41558-018-0282-y.

28. Kotz, M., Wenz, L., Stechemesser, A., Kalkuhl, M., & Levermann, A. Day-to-day temperature variability reduces economic growth. *Nature Climate Change* **11**, 319–325 (2021). DOI: 10.1038/s41558-020-00985-5.

29. Choudhury, D., Ji, F., Nishant, N., & Di Virgilio, G. Evaluation of ERA5-simulated temperature and its extremes for Australia. *Atmosphere* **14**, 913 (2023). DOI: 10.3390/atmos14060913.

30. Xu, W. et al. How well does the ERA5 reanalysis capture the extreme climate events over China? Part II: Extreme temperature. *Frontiers in Environmental Science* **10**, 921659 (2022). DOI: 10.3389/fenvs.2022.921659.

31. Arshad, M. et al. Performance evaluation of ERA-5, JRA-55, MERRA-2, and CFS-2 reanalysis datasets, over diverse climate regions of Pakistan. *Weather and Climate Extremes* **33**, 100373 (2021). DOI: 10.1016/j.wace.2021.100373.

32. Beck, H. E. et al. Daily evaluation of 26 precipitation datasets using Stage-IV gauge-radar data for the CONUS. *Hydrology and Earth System Sciences* **23**, 207–224 (2019). DOI: 10.5194/hess-23-207-2019.

33. Gbode, I. E., Babalola, T. E., Diro, G. T., & Intsiful, J. D. Assessment of ERA5 and ERA-Interim in reproducing mean and extreme climates over West Africa. *Advances in Atmospheric Sciences* **40**, 570–586 (2023). DOI: 10.1007/s00376-022-2161-8.

34. Lei, X. et al. How well does the ERA5 reanalysis capture the extreme climate events over China? Part I: Extreme precipitation. *Frontiers in Environmental Science* **10** (2022). DOI: 10.3389/fenvs.2022.921658.

35. Fisher, A. C., Hanemann, W. M., Roberts, M. J., & Schlenker, W. The economic impacts of climate change: Evidence from agricultural output and random fluctuations in weather: Comment. *American Economic Review* **102**, 3749–3760 (2012). DOI: 10.1257/aer.102.7.3749.

36. Beck, H. E. et al. Global-scale evaluation of 22 precipitation datasets using gauge observations and hydrological modeling. *Hydrology and Earth System Sciences* **21**, 6201–6217 (2017). DOI: 10.5194/hess-21-6201-2017.

37. Hersbach, H. et al. The ERA5 global reanalysis. *Quarterly Journal of the Royal Meteorological Society* **146**, 1999–2049 (2020). DOI: 10.1002/qj.3803.

38. GADM. GADM data. URL: https://gadm.org/data.html.

39. Kotz, M., Levermann, A., & Wenz, L. Data and code for the publication "The effect of rainfall changes on economic production", Zenodo. (2021). DOI: 10.5281/zenodo.5657457.

40. Wenz, L., Carr, R. D., Kögel, N., Kotz, M., & Kalkuhl, M. DOSE - Global data set of reported sub-national economic output. *Scientific Data* **10**, 425 (2023). DOI: 10.1038/s41597-023-02323-8.

41. Yumashev, D. et al. Climate policy implications of nonlinear decline of Arctic land permafrost and other cryosphere elements. *Nature Communications* **10**, 1900 (2019). DOI: 10.1038/s41467-019-09863-x.

42. Kikstra, J. S. et al. The social cost of carbon dioxide under climate-economy feedbacks and temperature variability. *Environmental Research Letters* **16**, 094037 (2021). DOI: 10.1088/1748-9326/ac1d0b.

43. Dietz, S., Rising, J., Stoerk, T., & Wagner, G. Economic impacts of tipping points in the climate system. *Proceedings of the National Academy of Sciences of the United States of America* **118**, e2103081118 (2021). DOI: 10.1073/pnas.2103081118.

44. Dell, M., Jones, B. F., & Olken, B. A. Temperature shocks and economic growth: Evidence from the last half century. *American Economic Journal: Macroeconomics* **4**, 66–95 (2012). DOI: 10.1257/mac.4.3.66.

45. Burke, M. & Emerick, K. Adaptation to climate change: Evidence from US agriculture. *American Economic Journal: Economic Policy* **8**, 106–140 (2016). DOI: 10.1257/pol.20130025.

46. Bastien-Olvera, B. A., Granella, F., & Moore, F. C. Persistent effect of temperature on GDP identified from lower frequency temperature variability. *Environmental Research Letters* **17**, 084038 (2022). DOI: 10.1088/1748-9326/ac82c2.

47. Piontek, F. et al. Integrated perspective on translating biophysical to economic impacts of climate change. *Nature Climate Change* **11**, 563–572 (2021). DOI: 10.1038/s41558-021-01065-y.

48. Moore, F. C. & Diaz, D. B. Temperature impacts on economic growth warrant stringent mitigation policy. *Nature Climate Change* **5**, 127–131 (2015). DOI: 10.1038/nclimate2481.

49. Kahn, M. E. et al. Long-term macroeconomic effects of climate change: A cross-country analysis. *Energy Economics* **104**, 105624 (2021). DOI: 10.1016/j.eneco.2021.105624.

50. Newell, R. G., Prest, B. C., & Sexton, S. E. The GDP-temperature relationship: Implications for climate change damages. *Journal of Environmental Economics and Management* **108**, 102445 (2021). DOI: 10.1016/j.jeem.2021.102445.

51. Barrage, L. & Nordhaus, W. D. Policies, projections, and the social cost of carbon: Results from the DICE-2023 model. NBER Working Paper Series 31112 (2023). DOI: 10.3386/w31112.

52. Lehner, F. et al. Partitioning climate projection uncertainty with multiple large ensembles and CMIP5/6. *Earth System Dynamics* **11**, 491–508 (2020). DOI: 10.3929/ethz-b-000418969.

53. Beusch, L., Gudmundsson, L., & Seneviratne, S. I. Crossbreeding CMIP6 earth system models with an emulator for regionally optimized land temperature projections. *Geophysical Research Letters* **47**, e2019GL086812 (2020). DOI: 10.1029/2019GL086812.

54. Zscheischler, J. et al. Future climate risk from compound events. *Nature Climate Change* **8**, 469–477 (2018). DOI: 10.1038/s41558-018-0156-3.

55. Damania, R., Desbureaux, S., & Zaveri, E. Does rainfall matter for economic growth? Evidence from global sub-national data (1990–2014). *Journal of Environmental Economics and Management* **102**, 102335 (2020). DOI: 10.1016/j.jeem.2020.102335.

56. Palagi, E., Coronese, M., Lamperti, F., & Roventini, A. Climate change and the nonlinear impact of precipitation anomalies on income inequality. *Proceedings of the National Academy of Sciences of the United States of America* **119**, e2203595119 (2022). DOI: 10.1073/pnas.2203595119.

57. Linsenmeier, M. Temperature variability and long-run economic development. *Journal of Environmental Economics and Management* **121**, 102840 (2023). DOI: 10.1016/j.jeem.2023.102840.

58. Auffhammer, M., Hsiang, S. M., Schlenker, W., & Sobel, A. Using weather data and climate model output in economic analyses of climate change. *Review of Environmental Economics and Policy* **7**, 181–198 (2013). DOI: 10.1093/reep/ret016.

59. Dai, A. & National Center for Atmospheric Research Staff. The Climate Data Guide: Palmer Drought Severity Index (PDSI). Retrieved from https://climatedataguide.ucar.edu/climate-data/palmer-drought-severity-index-pdsi on 2023-11-20. (2023).
