# Supplementary material for: Climate damage projections beyond annual temperature
Source: Nat Clim Chang. 2024 Apr 17;14(6):592–9. doi: 10.1038/s41558-024-01990-8 (PMC11446829; doi:10.1038/s41558-024-01990-8)
Supplement: Supplementary file 2 — Reporting Summary [file 41558_2024_1990_MOESM2_ESM.pdf]

nature portfolio

Corresponding author(s): Paul Waidelich

Last updated by author(s): Mar 17, 2024

Reporting Summary

Nature Portfolio wishes to improve the reproducibility of the work that we publish. This form provides structure for consistency and transparency in reporting. For further information on Nature Portfolio policies, see our Editorial Policies and the Editorial Policy Checklist.

Statistics

For all statistical analyses, confirm that the following items are present in the figure legend, table legend, main text, or Methods section.

n/a	Confirmed	
☒	☐	The exact sample size (*n*) for each experimental group/condition, given as a discrete number and unit of measurement
☒	☐	A statement on whether measurements were taken from distinct samples or whether the same sample was measured repeatedly
☒	☐	The statistical test(s) used AND whether they are one- or two-sided<br>*Only common tests should be described solely by name; describe more complex techniques in the Methods section.*
☐	☒	A description of all covariates tested
☒	☐	A description of any assumptions or corrections, such as tests of normality and adjustment for multiple comparisons
☐	☒	A full description of the statistical parameters including central tendency (e.g. means) or other basic estimates (e.g. regression coefficient) AND variation (e.g. standard deviation) or associated estimates of uncertainty (e.g. confidence intervals)
☒	☐	For null hypothesis testing, the test statistic (e.g. *F*, *t*, *r*) with confidence intervals, effect sizes, degrees of freedom and *P* value noted<br>*Give P values as exact values whenever suitable.*
☒	☐	For Bayesian analysis, information on the choice of priors and Markov chain Monte Carlo settings
☒	☐	For hierarchical and complex designs, identification of the appropriate level for tests and full reporting of outcomes
☒	☐	Estimates of effect sizes (e.g. Cohen's *d*, Pearson's *r*), indicating how they were calculated

Our web collection on statistics for biologists contains articles on many of the points above.

Software and code

Policy information about availability of computer code

Data collection	RStudio Server (version 2022.07.2) based on R (version 4.2.2) and JupyterLab (version 3.6.2) based on Python (version 3.9.10) was used.
Data analysis	RStudio Server (version 2022.07.2) based on R (version 4.2.2) and JupyterLab (version 3.6.2) based on Python (version 3.9.10) was used.

For manuscripts utilizing custom algorithms or software that are central to the research but not yet described in published literature, software must be made available to editors and reviewers. We strongly encourage code deposition in a community repository (e.g. GitHub). See the Nature Portfolio guidelines for submitting code & software for further information.

Data

Policy information about availability of data

All manuscripts must include a data availability statement. This statement should provide the following information, where applicable:

- Accession codes, unique identifiers, or web links for publicly available datasets
- A description of any restrictions on data availability
- For clinical datasets or third party data, please ensure that the statement adheres to our policy

CMIP6 temperature and precipitation indicators are available on the ETH Zurich CMIP6 repository (https://doi.org/10.5281/ZENODO.3734128; Brunner et al., 2020). CESM2 large ensemble outputs are available at https://www.earthsystemgrid.org/dataset/ucar.cgd.cesm2le.output.html. Tx5d and PDSI values from Callahan & Mankin (2022) (used in Appendix F) are available at https://github.com/ccallahan45/CallahanMankin_ExtremeHeatEconomics_2022. Scripts to estimate the dose-response functions deployed here and the underlying climate and economic data from Kotz et al. (2022) are available at https://zenodo.org/record/5657457 (Kotz

et al., 2021). ERA5 reanalysis data is available at https://www.ecmwf.int/en/forecasts/datasets/reanalysis-datasets/era5. Source data are provided with this paper. All additional data are publicly available at https://doi.org/10.5281/zenodo.10825603 (Waidelich et al., 2024).

Human research participants

Policy information about studies involving human research participants and Sex and Gender in Research.

Reporting on sex and gender	N/A - No human research participants
Population characteristics	N/A - No human research participants
Recruitment	N/A - No human research participants
Ethics oversight	N/A - No human research participants

Note that full information on the approval of the study protocol must also be provided in the manuscript.

Field-specific reporting

Please select the one below that is the best fit for your research. If you are not sure, read the appropriate sections before making your selection.

☐ Life sciences ☐ Behavioural & social sciences ☒ Ecological, evolutionary & environmental sciences

For a reference copy of the document with all sections, see nature.com/documents/nr-reporting-summary-flat.pdf

Ecological, evolutionary & environmental sciences study design

All studies must disclose on these points even when the disclosure is negative.

Study description	Projections of economic income impacts of six different climate indicators (annual mean temperature, annual total precipitation, day-to-day temperature variability, extreme precipitation, monthly precipitation deviation, number of wet days) under different global warming levels until 2100.
Research sample	Based on daily temperature and precipitation information between 1850-2100 from 33 CMIP6 models, including two large ensembles, and 3 RCP-SSP pairs (SSP1-1.9, SSP1-2.6, and SSP3-7.0), resulting in N=199 model-scenario combinations.
Sampling strategy	All CMIP6 models for which the six climate indicators of interest could be computed for the same ensemble realization in the ETH Zurich CMIP6 next-generation archive were used. To account for statistical uncertainty about the dose-response function parameters, a Monte Carlo sample size of N=1,000 was chosen following the extant literature on top-down damage function projections.
Data collection	We calculate annual climate indicators at the grid cell level for each model-scenario pair, aggregate them to ADM1-level subnational regions, and then feed them through indicator-specific dose-response functions, using a different draw from the multivariate distribution of dose-response function parameters for each Monte Carlo run. The resulting annual economic damage projections are then further aggregated to the country or global level and mapped to global warming levels based on the global mean surface temperature time series for the underlying model-realization-scenario pairing. Economic damage projections are calculated for each climate indicator separately and for combinations of indicators.
Timing and spatial scale	We retrieved daily temperature and precipitation data from each model and employed conservative remapping (Jones, 1999) to regrid data onto a common 2.5°×2.5° longitude–latitude grid. This regridding enables comparison across different models. Subsequently, we calculated annual temperature and precipitation indicators for the period 1850-2100. To ensure compatibility with ERA5 reanalysis, we further regridded the annual temperature and precipitation indicators from the original 2.5° resolution to a finer 0.25° grid resolution.
Data exclusions	No observations were excluded from the data analysis. However, the study's global and country-level figures and results only feature sovereign ADM0-level territories since historical data on economic income and future projections are often unavailable for non-sovereign territories. The omitted non-sovereign ADM0-level territories are listed in the Supplementary Information.
Reproducibility	N/A - No experiments performed
Randomization	N/A - No experiments performed
Blinding	N/A - No experiments performed

Did the study involve field work? ☐ Yes ☒ No

Reporting for specific materials, systems and methods

We require information from authors about some types of materials, experimental systems and methods used in many studies. Here, indicate whether each material, system or method listed is relevant to your study. If you are not sure if a list item applies to your research, read the appropriate section before selecting a response.

Materials & experimental systems

n/a	Involved in the study
☒	☐ Antibodies
☒	☐ Eukaryotic cell lines
☒	☐ Palaeontology and archaeology
☒	☐ Animals and other organisms
☒	☐ Clinical data
☒	☐ Dual use research of concern

Methods

n/a	Involved in the study
☒	☐ ChIP-seq
☒	☐ Flow cytometry
☒	☐ MRI-based neuroimaging
